# Supplementary material for: Total wash elimination for solid phase peptide synthesis
Source: Nat Commun. 2023 Dec 9;14:8168. doi: 10.1038/s41467-023-44074-5 (PMC10710472; doi:10.1038/s41467-023-44074-5)
Supplement: Supplementary file 1 — Supplementary Information [file 41467_2023_44074_MOESM1_ESM.pdf]

Supplementary Information

Total Wash Elimination for Solid Phase Peptide Synthesis

Jonathan M. Collins*, Sandeep K. Singh, Travis A. White, Drew J. Cesta, Colin L. Simpson, Levi J. Tubb & Christopher L. Houser

Peptide Synthesis Research, CEM Corporation, 3100 Smith Farm Rd, Matthews, NC 28104, USA

Table of Contents

1. Materials — Page 2
2. Supplementary Fig. 1 Microwave SPPS reaction vessel schematic — Page 3
3. Supplementary Table 1 Results from wash-free and wash based Fmoc SPPS — Page 4
4. Supplementary Table 2 Total synthesis time and total waste volume from automated microwave peptide/protein synthesis using wash-free methodology — Page 4
5. Purity Reports and UPLC-MS Chromatograms for Table 1 — Page 5-26
6. Purity Reports and UPLC-MS Chromatograms for Supplementary Table 1 — Page 27-37
7. C.A.T. GmbH Epimerization Data for 25 mmol Liraglutide Production Run — Page 38-54
8. Supplementary Table 3 Stepwise method details for wash-free 0.1 mmol research scale synthesis — Page 55
9. Supplementary Table 4 Stepwise method details for wash-free 25 mmol production scale synthesis — Page 56
10. Supplementary Table 5 Stepwise method details for residual pyrrolidine quantification by GC-FID — Page 57
11. GC-FID calibration curve — Page 59
12. GC-FID chromatograms — Page 60-61

Materials

The following Fmoc amino acids were obtained from CEM Corporation and contain the indicated side chain protecting groups: Ala, Asn(Trt), Arg(Pbf), Asp(OMpe), Cys(Trt), Gln(Trt), Glu(OtBu), Gly, His(Boc), Ile, Leu, Lys(Boc), Phe, Pro, Met, Ser(tBu), Thr(tBu), Trp(Boc), Tyr(tBu), and Val. Fmoc-Lys(palmitoyl-Glu-OtBu)-OH, Oxyma, Rink Amide ProTide and preloaded Wang ProTide resins were also obtained from CEM Corporation. Fmoc-Rink Amide MBHA PS resin was obtained from MilliporeSigma. *N,N'*-Diisopropylcarbodiimide (DIC), pyrrolidine, piperidine, trifluoroacetic acid (TFA), 3,6-dioxa-1,8-octanedithiol (DODT), and triisopropylsilane (TIS) were obtained from Sigma-Aldrich. Dichloromethane (DCM), *N,N*-Dimethylformamide (DMF), N-methylpyrrolidone (NMP), anhydrous diethyl ether (Et_2O), and acetic acid were obtained from VWR. LC-MS grade water (H_2O) and LC-MS grade acetonitrile (MeCN) were obtained from Fisher Scientific. N-butylpyrrolidine (NBP) was obtained from CEM Corporation.

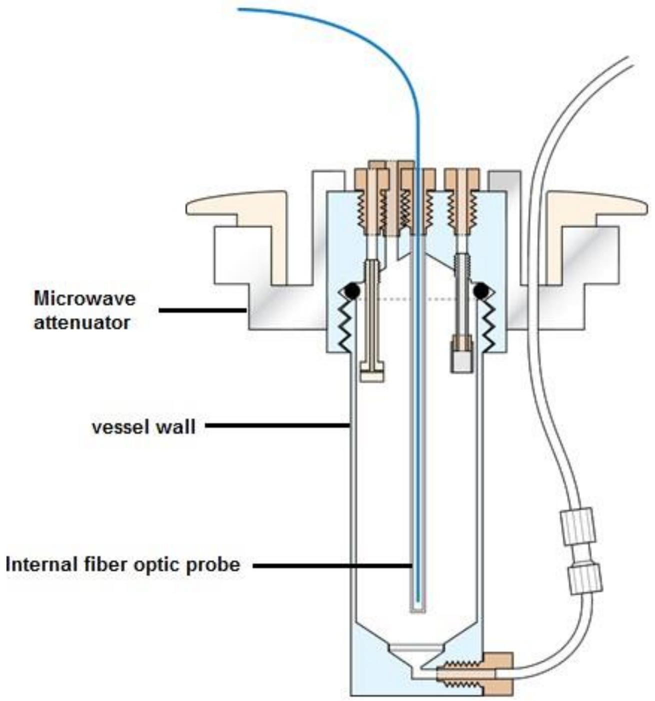

Supplementary Fig. 1 Microwave SPPS reaction vessel schematic.

Supplementary Table 1 Results from wash-free and wash based Fmoc SPPS.

Entry	Peptide	Deprotection Temp.	Deprotection Time	Crude Purity (Wash-free)	Crude Purity (Wash based)
**1**	$^{65\text{-}74}$ACP	110 °C	80 sec	91%	91%
**2**	Liraglutide	110 °C	80 sec	73%	74%
**3**	Semaglutide	110 °C	80 sec	71%	74%
**4**	$^{1\text{-}42}$β-amyloid	110 °C	80 sec	69%	69%
**5**	Liraglutide	80 °C	8 min	72%	--
**6***	Liraglutide	90 °C	10 min	77%	78%#

*Synthesized at 25 mmol scale; #80 °C/4 min Deprotection method was used

Supplementary Table 2 Total synthesis time and total waste volume from automated microwave peptide/protein synthesis using wash-free methodology.

Entry	Peptide/Protein	Total Synthesis Time	Total Waste Volume
**1**	JR	38 min	43 mL
**2**	$^{65\text{-}74}$ACP	35 min	39 mL
**3**	Liraglutide	1 h 52 min	135 mL
**4**	$^{1\text{-}42}$β-Amyloid	2 h 29 min	179 mL
**5**	Proinsulin 86-mer	10 h 40 min	687 mL
**6**	Barstar 89-mer	10 h 56 min	700 mL
**7**	Semaglutide	1 h 51 min	135 mL

JR-Table 1, entry 1

Chromatogram and Results

Injection Details

Injection Name:	TW_JR WASH, no HS clean 0.1mmol PRIME_lyoph_40s 25% depro		Run Time (min):	6.50
Vial Number:	B:D1		Injection Volume:	10.00
Injection Type:	Unknown		Channel:	UV_VIS_1
Calibration Level:			Wavelength:	210
Instrument Method:	ACN10-70_0.500uL_40C	Waters Acquity BEH C8 1.7um. 2.1 x 100mm Column	Bandwidth:	4
Processing Method:	Quantitative		Dilution Factor:	1.0000
Injection Date/Time:	12/Dec/22 09:32		Sample Weight:	1.0000

Chromatogram

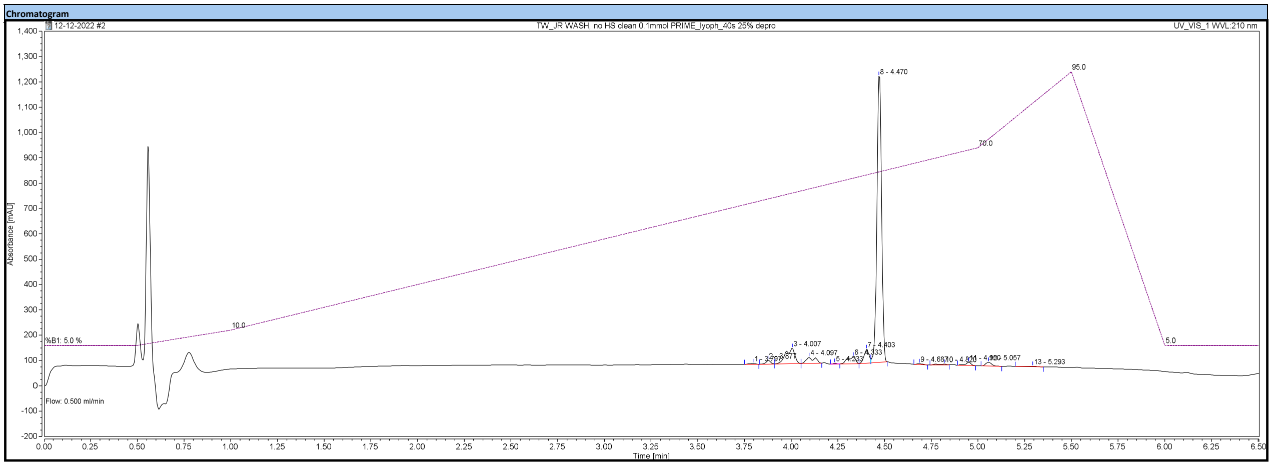

No.	Retention Time	Area	Height	Relative Area	Relative Height
	min	mAU*min	mAU	%	%
UV_VIS_1	UV_VIS_1	UV_VIS_1	UV_VIS_1	UV_VIS_1	UV_VIS_1
1	3.797	0.118	2.924	0.28	0.22
2	3.877	0.395	14.209	0.92	1.05
3	4.007	3.443	60.637	8.07	4.49
4	4.097	1.286	22.981	3.01	1.70
5	4.233	0.061	2.725	0.14	0.20
6	4.333	1.422	27.105	3.33	2.01
7	4.403	1.647	53.861	3.86	3.99
8	4.470	32.859	1129.249	77.03	83.70
9	4.687	0.106	3.182	0.25	0.24
10	4.820	0.180	2.933	0.42	0.22
11	4.950	0.444	12.486	1.04	0.93
12	5.057	0.548	14.919	1.29	1.11
13	5.293	0.150	1.959	0.35	0.15
Total:		42.657	1349.170	100.00	100.00

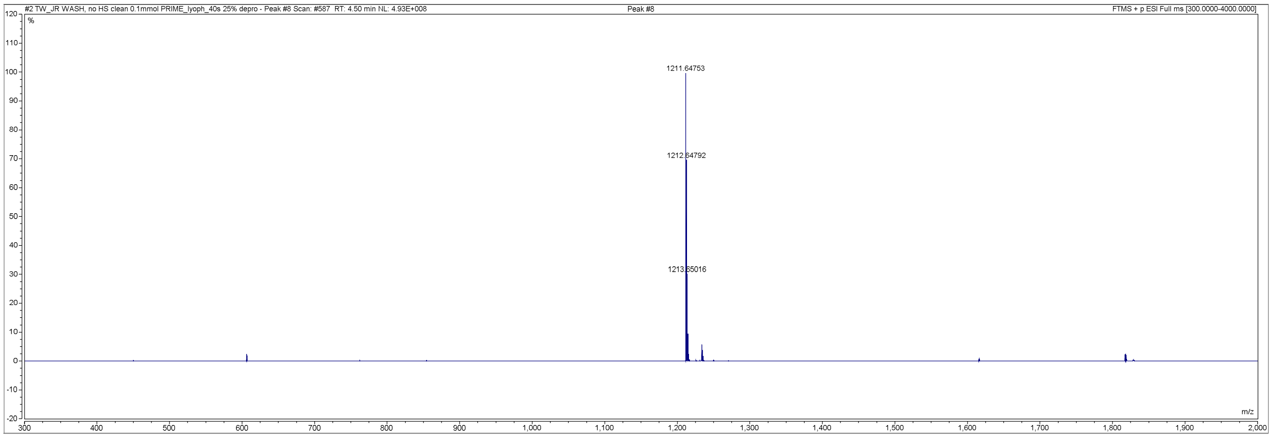

JR-Table 1, entry 2

Chromatogram and Results

Injection Details

Injection Name:	*TW_JR WASH, 0.1mmol, PRIME, DMF_lyoph_40s, 25% depro (NH4OH)-dil*		*Run Time (min):*	*6.50*
Vial Number:	*B:B5*		*Injection Volume:*	*20.00*
Injection Type:	*Unknown*		*Channel:*	*UV_VIS_1*
Calibration Level:			*Wavelength:*	*210*
Instrument Method:	*ACN10-70_0.500uL_40C*	*Waters Acquity BEH C8 1.7um. 2.1 x 100mm Column*	*Bandwidth:*	*4*
Processing Method:	*Quantitative*		*Dilution Factor:*	*1.0000*
Injection Date/Time:	*03/Jan/23 15:44*		*Sample Weight:*	*1.0000*

Chromatogram

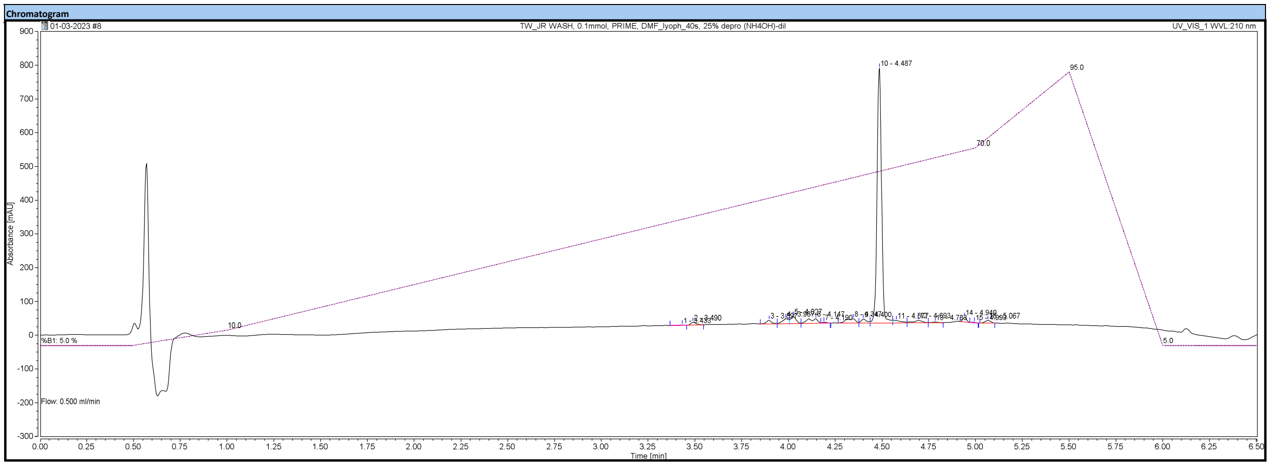

No.	Retention Time	Area	Height	Relative Area	Relative Height
	min	mAU*min	mAU	%	%
UV_VIS_1	UV_VIS_1	UV_VIS_1	UV_VIS_1	UV_VIS_1	UV_VIS_1
1	3.433	0.024	0.669	0.09	0.08
2	3.490	0.267	7.849	1.01	0.88
3	3.897	0.357	10.464	1.35	1.18
4	3.987	0.580	14.690	2.19	1.65
5	4.027	0.706	21.431	2.66	2.41
6	4.147	0.860	13.361	3.25	1.50
7	4.190	0.048	1.980	0.18	0.22
8	4.347	0.753	12.838	2.84	1.44
9	4.400	0.405	11.956	1.53	1.34
10	4.487	20.914	755.001	78.96	84.82
11	4.577	0.394	7.146	1.49	0.80
12	4.693	0.423	7.586	1.60	0.85
13	4.783	0.101	2.284	0.38	0.26
14	4.940	0.331	13.867	1.25	1.56
15	4.993	0.033	1.386	0.12	0.16
16	5.067	0.290	7.658	1.09	0.86
Total:		26.486	890.167	100.00	100.00

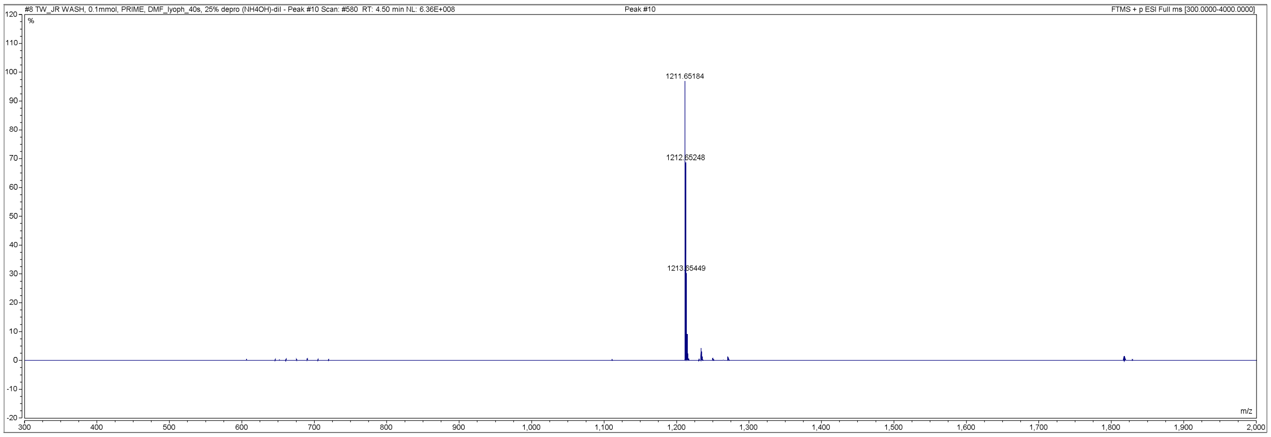

JR-Table 1, entry 3

Chromatogram and Results

Injection Details

Injection Name:	*TW_JR no wash, no HS clean 0.1mmol PRIME_lyoph_40s 25% depro*		*Run Time (min):*	*6.50*
Vial Number:	*B:D3*		*Injection Volume:*	*10.00*
Injection Type:	*Unknown*		*Channel:*	*UV_VIS_1*
Calibration Level:			*Wavelength:*	*210*
Instrument Method:	*ACN10-70_0.500uL_40C*	*Waters Acquity BEH C8 1.7um. 2.1 x 100mm Column*	*Bandwidth:*	*4*
Processing Method:	*Quantitative*		*Dilution Factor:*	*1.0000*
Injection Date/Time:	*12/Dec/22 09:47*		*Sample Weight:*	*1.0000*

Chromatogram

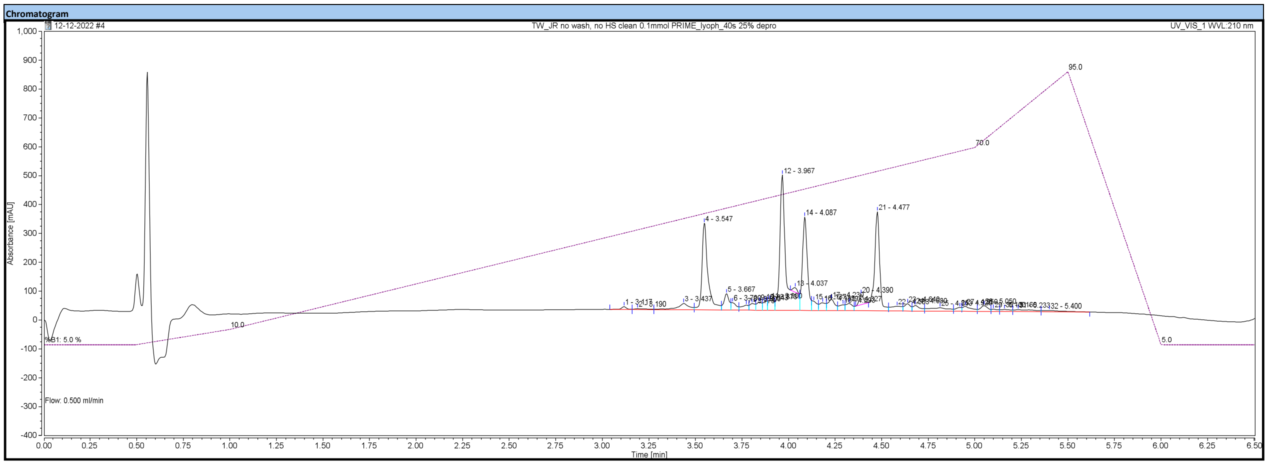

No.	Retention Time	Area	Height	Relative Area	Relative Height
	min	mAU*min	mAU	%	%
UV_VIS_1	UV_VIS_1	UV_VIS_1	UV_VIS_1	UV_VIS_1	UV_VIS_1
1	3.117	0.337	10.044	0.44	0.49
2	3.190	0.248	2.977	0.32	0.15
3	3.437	1.684	22.265	2.17	1.10
4	3.547	11.102	300.340	14.33	14.79
5	3.667	1.788	56.581	2.31	2.79
6	3.700	0.839	25.857	1.08	1.27
7	3.773	0.751	17.442	0.97	0.86
8	3.803	0.764	23.219	0.99	1.14
9	3.843	0.926	28.302	1.20	1.39
10	3.873	0.781	30.235	1.01	1.49
11	3.900	1.312	35.091	1.69	1.73
12	3.967	18.189	468.501	23.47	23.07
13	4.037	0.405	15.766	0.52	0.78
14	4.087	10.052	323.366	12.97	15.92
15	4.137	1.046	32.102	1.35	1.58
16	4.183	1.076	26.822	1.39	1.32
17	4.230	1.683	39.530	2.17	1.95
18	4.293	0.742	20.237	0.96	1.00
19	4.327	1.052	25.238	1.36	1.24
20	4.390	0.972	35.216	1.25	1.73
21	4.477	12.797	343.269	16.51	16.90
22	4.583	1.126	16.066	1.45	0.79
23	4.640	0.973	25.468	1.26	1.25
24	4.680	0.886	18.993	1.14	0.94
25	4.813	1.468	12.021	1.89	0.59
26	4.920	0.471	13.216	0.61	0.65
27	4.950	0.959	17.043	1.24	0.84
28	5.050	0.931	19.980	1.20	0.98
29	5.100	0.321	7.918	0.41	0.39
30	5.160	0.425	6.790	0.55	0.33
31	5.233	0.830	6.757	1.07	0.33
32	5.400	0.560	4.471	0.72	0.22
Total:		77.497	2031.120	100.00	100.00

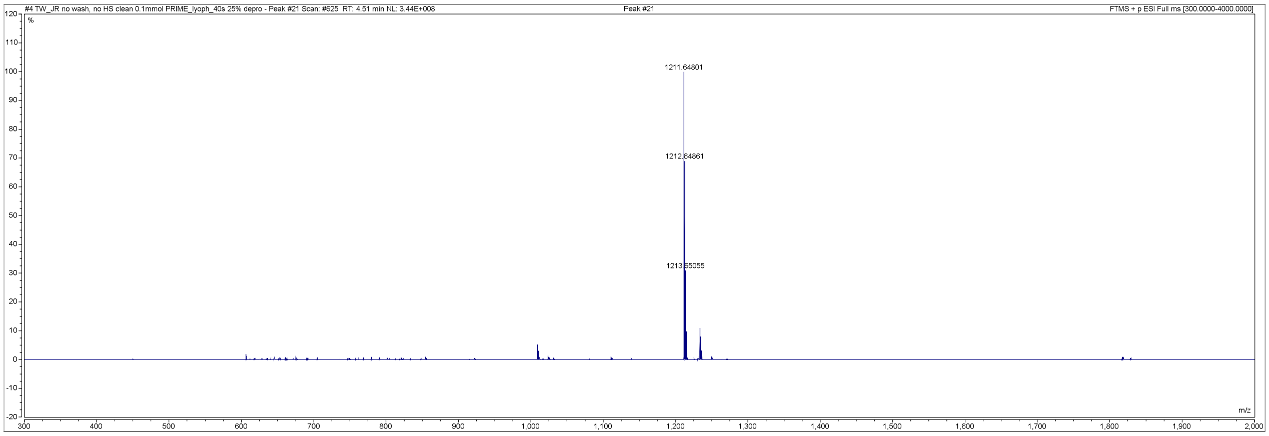

JR-Table 1, entry 4

Chromatogram and Results

Injection Details

Injection Name:	*TW_JR complete no wash_lyophilized_40s depro, 25% depro (8/15 compare)*	*Run Time (min):*	*6.50*
Vial Number:	*Y:A6*	*Injection Volume:*	*2.50*
Injection Type:	*Unknown*	*Channel:*	*UV_VIS_1*
Calibration Level:		*Wavelength:*	*210*
Instrument Method:	*ACN10-70_0.500uL_40C Waters Acquity BEH C8 1.7um. 2.1 x 100mm Column*	*Bandwidth:*	*4*
Processing Method:	*Quantitative*	*Dilution Factor:*	*1.0000*
Injection Date/Time:	*02/Sep/22 10:11*	*Sample Weight:*	*1.0000*

Chromatogram

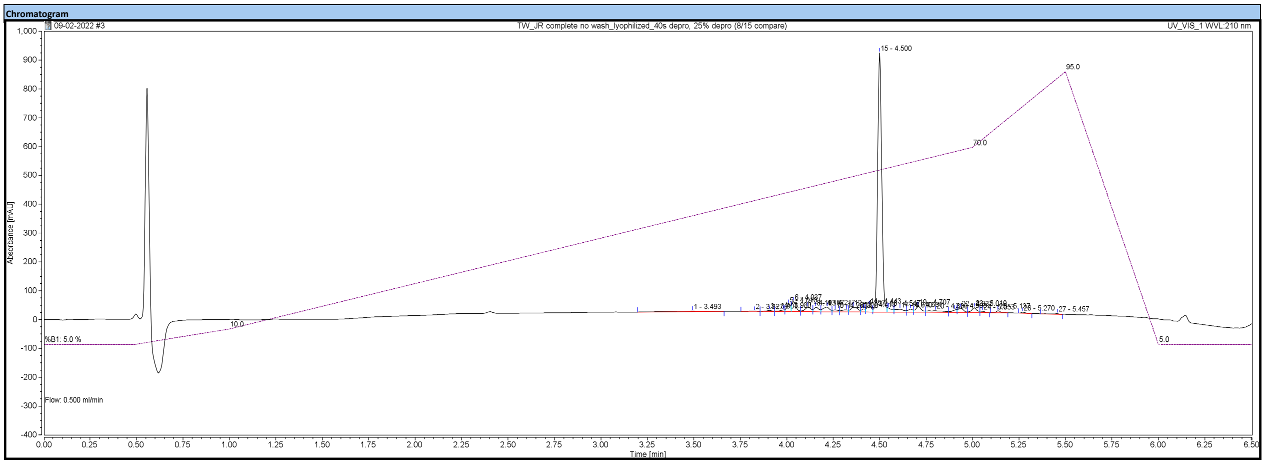

No.	Retention Time	Area	Height	Relative Area	Relative Height
	min	mAU*min	mAU	%	%
UV_VIS_1	UV_VIS_1	UV_VIS_1	UV_VIS_1	UV_VIS_1	UV_VIS_1
1	3.493	0.408	2.292	1.23	0.19
2	3.827	0.071	1.408	0.21	0.12
3	3.907	0.143	3.753	0.43	0.31
4	3.980	0.180	5.611	0.54	0.47
5	4.010	0.595	24.863	1.79	2.07
6	4.037	0.921	34.323	2.77	2.86
7	4.113	0.589	15.617	1.77	1.30
8	4.157	0.476	15.807	1.43	1.32
9	4.217	0.665	16.154	2.00	1.35
10	4.260	0.216	6.504	0.65	0.54
11	4.320	0.299	7.295	0.90	0.61
12	4.357	0.719	16.237	2.16	1.35
13	4.413	0.232	10.362	0.70	0.86
14	4.443	0.637	22.768	1.91	1.90
15	4.500	22.752	899.216	68.36	74.91
16	4.547	0.427	14.447	1.28	1.20
17	4.610	0.552	10.274	1.66	0.86
18	4.667	0.332	10.976	1.00	0.91
19	4.707	0.724	20.483	2.18	1.71
20	4.800	0.559	5.903	1.68	0.49
21	4.903	0.276	8.438	0.83	0.70
22	4.937	0.506	14.968	1.52	1.25
23	5.010	0.544	16.711	1.64	1.39
24	5.053	0.136	4.634	0.41	0.39
25	5.137	0.155	6.172	0.47	0.51
26	5.270	0.083	3.072	0.25	0.26
27	5.457	0.088	2.109	0.26	0.18
Total:		33.285	1200.396	100.00	100.00

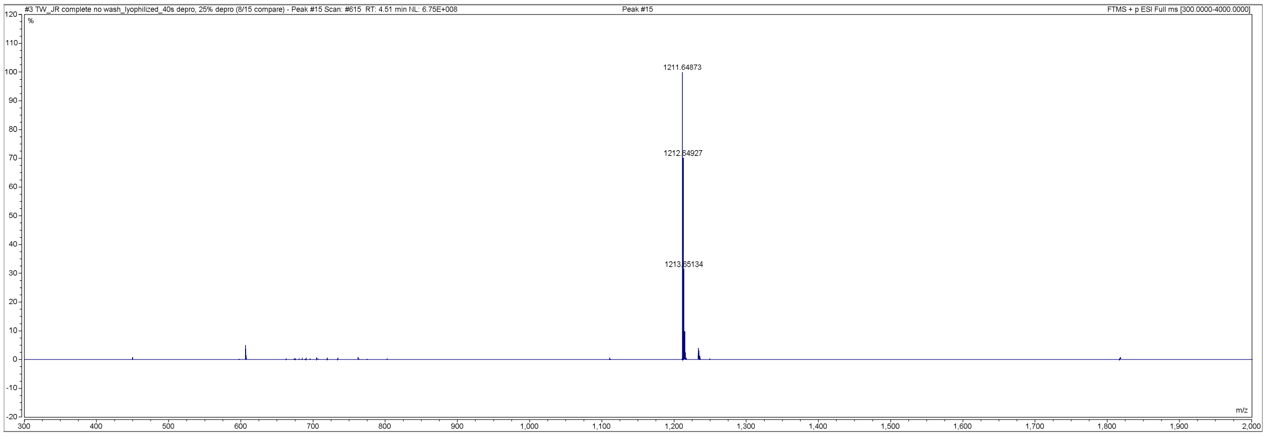

Chromeleon (c) Dionex
Version 7.2.10.23925

JR-Table 1, entry 5

Chromatogram and Results

Injection Details

Injection Name:	TW_JR no HS clean, no wash, std cond_lyophilized_25% depro, 80s depro, 10s drain	Run Time (min):	6.50
Vial Number:	Y:C9	Injection Volume:	20.00
Injection Type:	Unknown	Channel:	UV_VIS_1
Calibration Level:		Wavelength:	210
Instrument Method:	ACN10-70_0.500uL_40C Waters Acquity BEH C8 1.7um. 2.1 x 100mm Column	Bandwidth:	4
Processing Method:	Quantitative	Dilution Factor:	1.0000
Injection Date/Time:	18/Aug/22 09:05	Sample Weight:	1.0000

Chromatogram

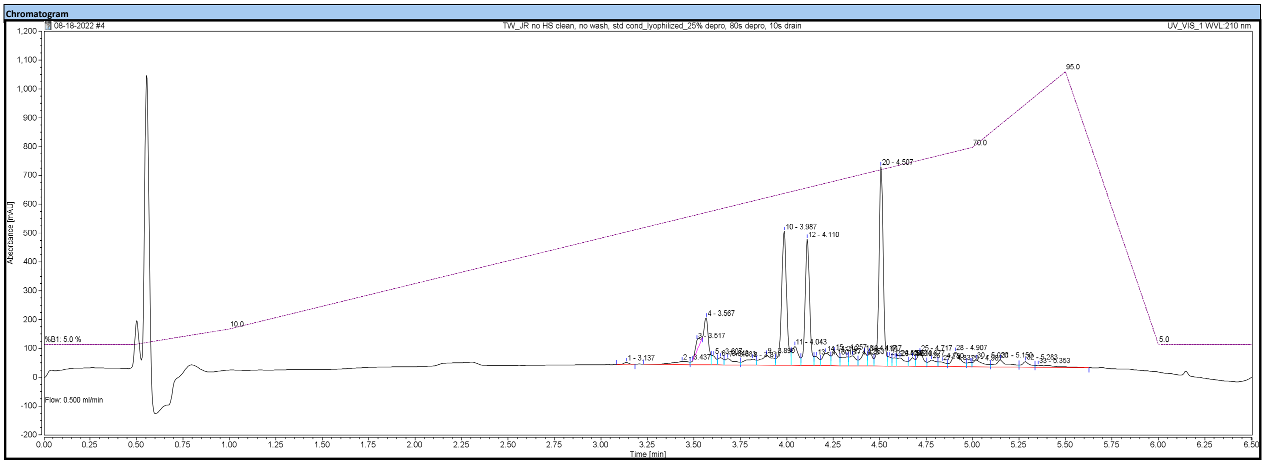

No.	Retention Time	Area	Height	Relative Area	Relative Height
	min	mAU*min	mAU	%	%
UV_VIS_1	UV_VIS_1	UV_VIS_1	UV_VIS_1	UV_VIS_1	UV_VIS_1
1	3.137	0.213	6.345	0.23	0.24
2	3.437	1.115	10.422	1.22	0.40
3	3.517	0.905	36.427	0.99	1.38
4	3.567	7.963	162.319	8.69	6.16
5	3.607	0.916	31.394	1.00	1.19
6	3.643	0.807	24.796	0.88	0.94
7	3.683	1.280	21.685	1.40	0.82
8	3.817	1.438	20.604	1.57	0.78
9	3.890	2.582	34.562	2.82	1.31
10	3.987	14.817	464.996	16.18	17.64
11	4.043	2.581	66.011	2.82	2.50
12	4.110	12.206	438.890	13.33	16.65
13	4.160	0.931	30.968	1.02	1.18
14	4.210	2.003	43.164	2.19	1.64
15	4.257	1.923	48.998	2.10	1.86
16	4.323	1.394	31.065	1.52	1.18
17	4.353	1.446	34.800	1.58	1.32
18	4.417	1.695	46.118	1.85	1.75
19	4.447	1.358	46.028	1.48	1.75
20	4.507	18.439	691.641	20.13	26.25
21	4.553	0.725	32.158	0.79	1.22
22	4.583	0.662	28.825	0.72	1.09
23	4.610	1.455	28.518	1.59	1.08
24	4.677	0.963	29.953	1.05	1.14
25	4.717	1.837	46.895	2.01	1.78
26	4.780	1.085	21.859	1.18	0.83
27	4.837	0.755	15.594	0.82	0.59
28	4.907	2.671	49.980	2.92	1.90
29	4.987	0.417	14.721	0.46	0.56
30	5.020	1.441	25.030	1.57	0.95
31	5.150	1.772	25.152	1.93	0.95
32	5.283	0.929	18.931	1.01	0.72
33	5.353	0.878	6.449	0.96	0.24
Total:		91.601	2635.299	100.00	100.00

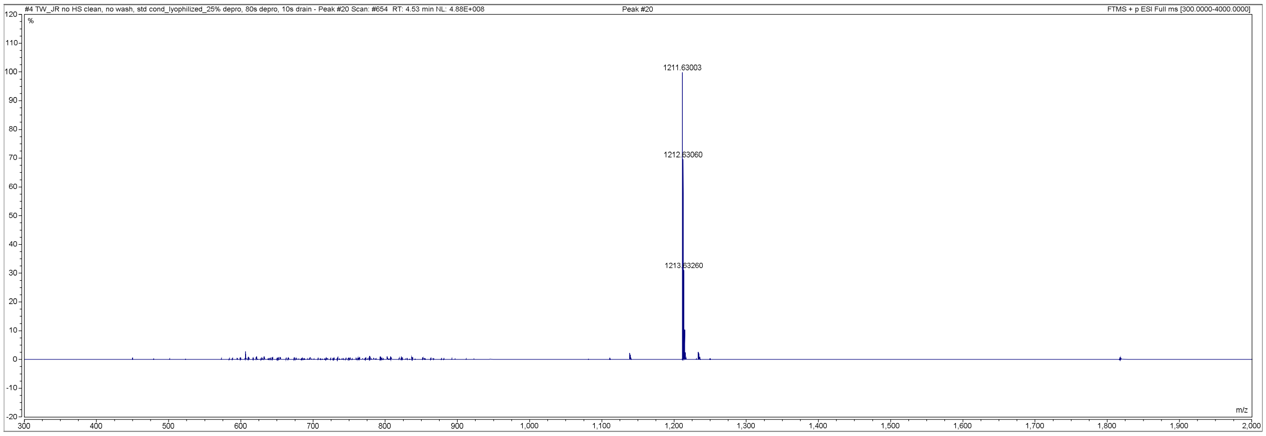

JR-Table 1, entry 6

Chromatogram and Results

Injection Details

Injection Name:	*TW_JR no wash, std cond, RA ProTide resin_lyoph_25% depro, 80s depro, 10s drain*	*Run Time (min):*	*6.50*
Vial Number:	*Y:B6*	*Injection Volume:*	*5.00*
Injection Type:	*Unknown*	*Channel:*	*UV_VIS_1*
Calibration Level:		*Wavelength:*	*210*
Instrument Method:	*ACN10-70_0.500uL_40C* *Waters Acquity BEH C8 1.7um. 2.1 x 100mm Column*	*Bandwidth:*	*4*
Processing Method:	*Quantitative*	*Dilution Factor:*	*1.0000*
Injection Date/Time:	*15/Aug/22 14:41*	*Sample Weight:*	*1.0000*

Chromatogram

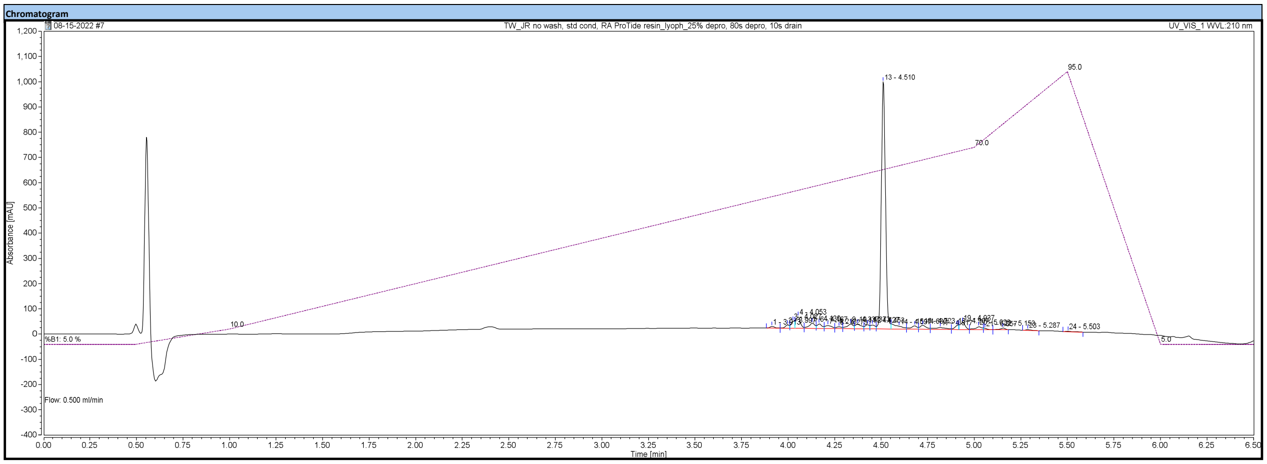

No.	Retention Time	Area	Height	Relative Area	Relative Height
	min	mAU*min	mAU	%	%
UV_VIS_1	UV_VIS_1	UV_VIS_1	UV_VIS_1	UV_VIS_1	UV_VIS_1
1	3.913	0.177	6.607	0.48	0.50
2	3.997	0.326	12.984	0.89	0.98
3	4.027	0.587	27.980	1.59	2.12
4	4.053	1.287	46.640	3.50	3.53
5	4.130	0.745	21.080	2.03	1.59
6	4.167	0.547	18.320	1.49	1.38
7	4.213	0.474	11.702	1.29	0.88
8	4.273	0.240	6.998	0.65	0.53
9	4.337	0.717	18.967	1.95	1.43
10	4.373	0.682	18.737	1.85	1.42
11	4.427	0.440	16.598	1.20	1.25
12	4.453	0.430	15.887	1.17	1.20
13	4.510	25.180	978.844	68.45	73.99
14	4.597	0.941	11.069	2.56	0.84
15	4.680	0.518	13.444	1.41	1.02
16	4.723	0.596	15.712	1.62	1.19
17	4.817	0.555	7.522	1.51	0.57
18	4.907	0.398	16.295	1.08	1.23
19	4.937	0.943	28.440	2.56	2.15
20	5.023	0.497	11.888	1.35	0.90
21	5.057	0.133	5.983	0.36	0.45
22	5.153	0.200	6.584	0.54	0.50
23	5.287	0.085	2.808	0.23	0.21
24	5.503	0.085	1.806	0.23	0.14
Total:		36.785	1322.894	100.00	100.00

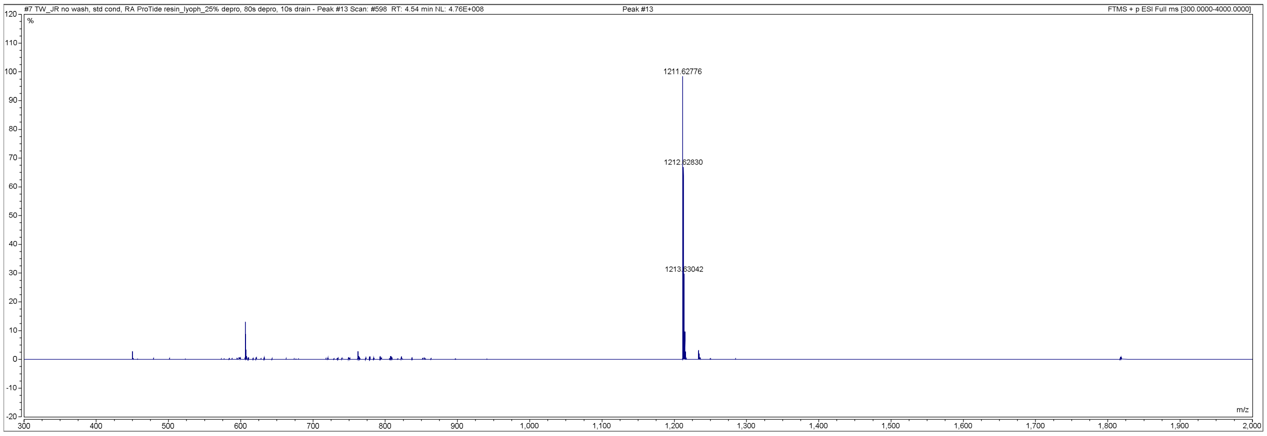

JR-Table 1, entry 7

Chromatogram and Results

Injection Details

Injection Name:	*TW_JR no wash, no HS clean 0.1mmol PRIME_lyoph_80s 20% depro*		*Run Time (min):*	*6.50*
Vial Number:	*B:D4*		*Injection Volume:*	*10.00*
Injection Type:	*Unknown*		*Channel:*	*UV_VIS_1*
Calibration Level:			*Wavelength:*	*210*
Instrument Method:	*ACN10-70_0.500uL_40C*	*Waters Acquity BEH C8 1.7um. 2.1 x 100mm Column*	*Bandwidth:*	*4*
Processing Method:	*Quantitative*		*Dilution Factor:*	*1.0000*
Injection Date/Time:	*12/Dec/22 09:54*		*Sample Weight:*	*1.0000*

Chromatogram

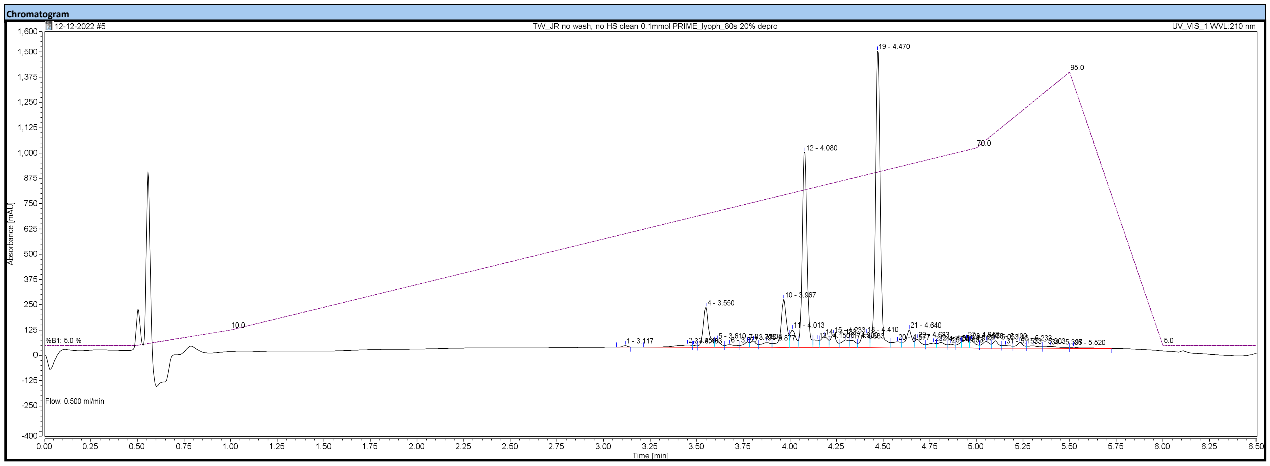

No.	Retention Time	Area	Height	Relative Area	Relative Height
	min	mAU*min	mAU	%	%
UV_VIS_1	UV_VIS_1	UV_VIS_1	UV_VIS_1	UV_VIS_1	UV_VIS_1
1	3.117	0.201	7.631	0.15	0.20
2	3.450	1.090	11.292	0.83	0.29
3	3.483	0.212	8.648	0.16	0.22
4	3.550	7.095	196.035	5.40	5.05
5	3.610	1.170	34.487	0.89	0.89
6	3.677	0.814	13.188	0.62	0.34
7	3.773	0.967	26.790	0.74	0.69
8	3.803	1.113	31.889	0.85	0.82
9	3.877	1.405	23.829	1.07	0.61
10	3.967	8.384	236.790	6.38	6.11
11	4.013	3.020	86.659	2.30	2.23
12	4.080	28.467	964.337	21.66	24.87
13	4.150	1.353	36.846	1.03	0.95
14	4.183	2.276	53.701	1.73	1.38
15	4.233	2.338	65.156	1.78	1.68
16	4.300	1.652	38.044	1.26	0.98
17	4.333	1.453	36.469	1.11	0.94
18	4.410	3.444	67.517	2.62	1.74
19	4.470	44.199	1466.113	33.64	37.80
20	4.577	1.759	29.854	1.34	0.77
21	4.640	3.329	88.427	2.53	2.28
22	4.683	1.748	42.565	1.33	1.10
23	4.770	1.245	23.551	0.95	0.61
24	4.810	1.265	27.535	0.96	0.71
25	4.863	0.721	18.143	0.55	0.47
26	4.917	0.667	27.109	0.51	0.70
27	4.947	1.601	43.057	1.22	1.11
28	4.973	1.354	37.562	1.03	0.97
29	5.053	1.518	31.835	1.16	0.82
30	5.100	1.330	36.184	1.01	0.93
31	5.157	0.693	12.313	0.53	0.32
32	5.233	1.216	28.500	0.93	0.73
33	5.303	0.822	11.289	0.63	0.29
34	5.397	0.955	9.038	0.73	0.23
35	5.520	0.525	5.906	0.40	0.15
Total:		131.398	3878.288	100.00	100.00

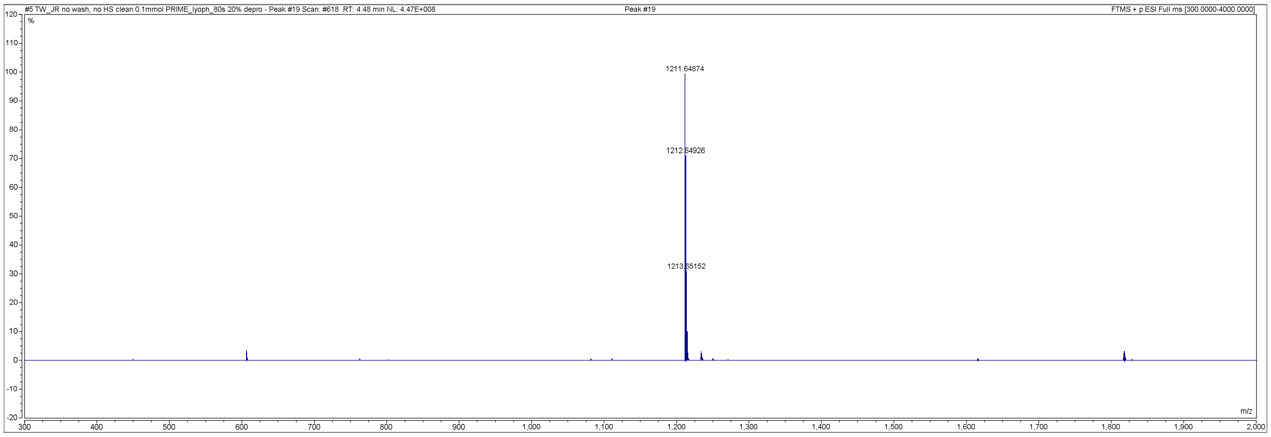

Chromeleon (c) Dionex
Version 7.2.10.23925

JR-Table 1, entry 8

Chromatogram and Results

Injection Details

Injection Name:	TW_JR 0.1 mmol no wash, std_lyoph_20% depro (80s depro) (CH3CN, NH4OH)	Run Time (min):	6.50
Vial Number:	Y:C3	Injection Volume:	3.00
Injection Type:	Unknown	Channel:	UV_VIS_1
Calibration Level:		Wavelength:	210
Instrument Method:	ACN10-70_0.500uL_40C Waters Acquity BEH C8 1.7um. 2.1 x 100mm Column	Bandwidth:	4
Processing Method:	Quantitative	Dilution Factor:	1.0000
Injection Date/Time:	16/Aug/22 15:34	Sample Weight:	1.0000

Chromatogram

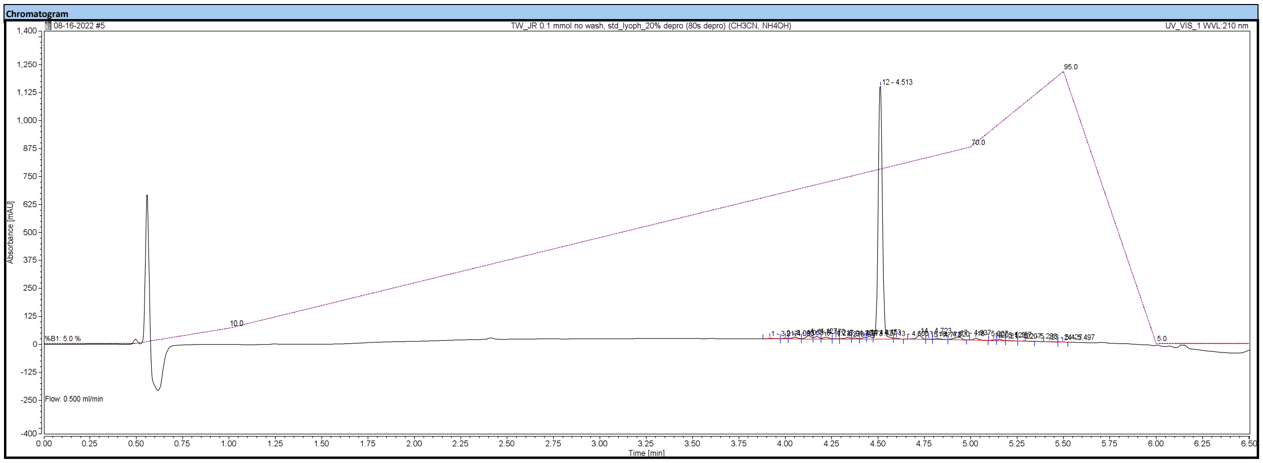

No.	Retention Time	Area	Height	Relative Area	Relative Height
	min	mAU*min	mAU	%	%
UV_VIS_1	UV_VIS_1	UV_VIS_1	UV_VIS_1	UV_VIS_1	UV_VIS_1
1	3.917	0.147	3.597	0.43	0.28
2	4.000	0.119	4.483	0.35	0.35
3	4.053	0.312	8.298	0.91	0.65
4	4.127	0.477	14.812	1.39	1.16
5	4.170	0.309	11.835	0.90	0.93
6	4.217	0.260	7.293	0.76	0.57
7	4.273	0.038	1.248	0.11	0.10
8	4.337	0.221	6.392	0.64	0.50
9	4.373	0.158	5.510	0.46	0.43
10	4.427	0.171	7.493	0.50	0.59
11	4.453	0.323	12.017	0.94	0.94
12	4.513	29.673	1128.485	86.42	88.52
13	4.600	0.131	4.151	0.38	0.33
14	4.723	0.553	17.345	1.61	1.36
15	4.773	0.050	1.620	0.14	0.13
16	4.820	0.172	4.704	0.50	0.37
17	4.937	0.517	11.696	1.50	0.92
18	5.027	0.272	9.104	0.79	0.71
19	5.123	0.110	4.476	0.32	0.35
20	5.157	0.157	5.568	0.46	0.44
21	5.207	0.050	1.117	0.15	0.09
22	5.293	0.065	1.892	0.19	0.15
23	5.427	0.028	0.872	0.08	0.07
24	5.497	0.023	0.830	0.07	0.07
Total:		34.335	1274.837	100.00	100.00

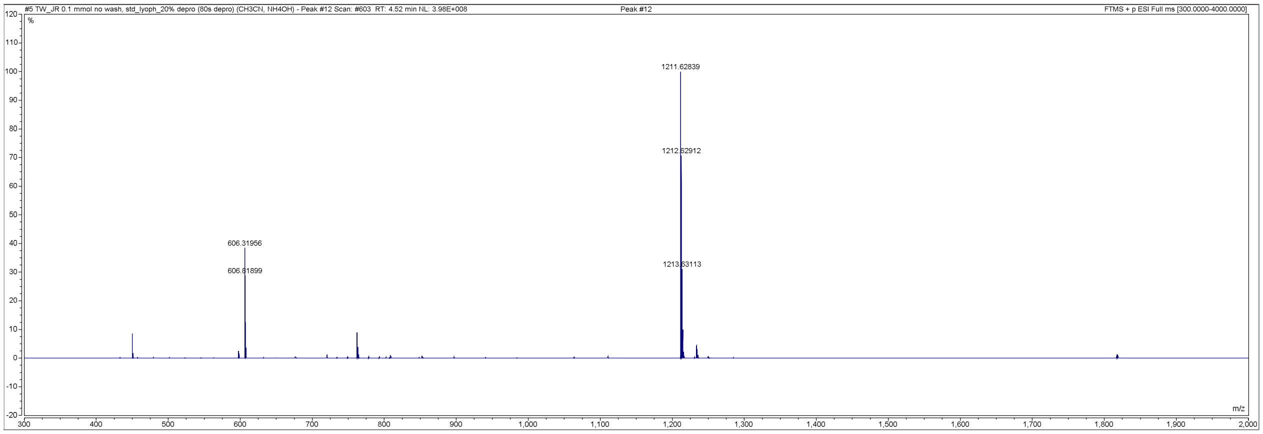

JR-Table 1, entry 9

Chromatogram and Results

Injection Details

Injection Name:	TW_JR 0.1 mmol no wash, no HS clean, std_lyoph_17% depro (80s depro) (CH3CN, NH4OH)		Run Time (min):	6.50
Vial Number:	Y:A4		Injection Volume:	5.00
Injection Type:	Unknown		Channel:	UV_VIS_1
Calibration Level:			Wavelength:	210
Instrument Method:	ACN10-70_0.500uL_40C	Waters Acquity BEH C8 1.7um. 2.1 x 100mm Column	Bandwidth:	4
Processing Method:	Quantitative		Dilution Factor:	1.0000
Injection Date/Time:	18/Aug/22 11:38		Sample Weight:	1.0000

Chromatogram

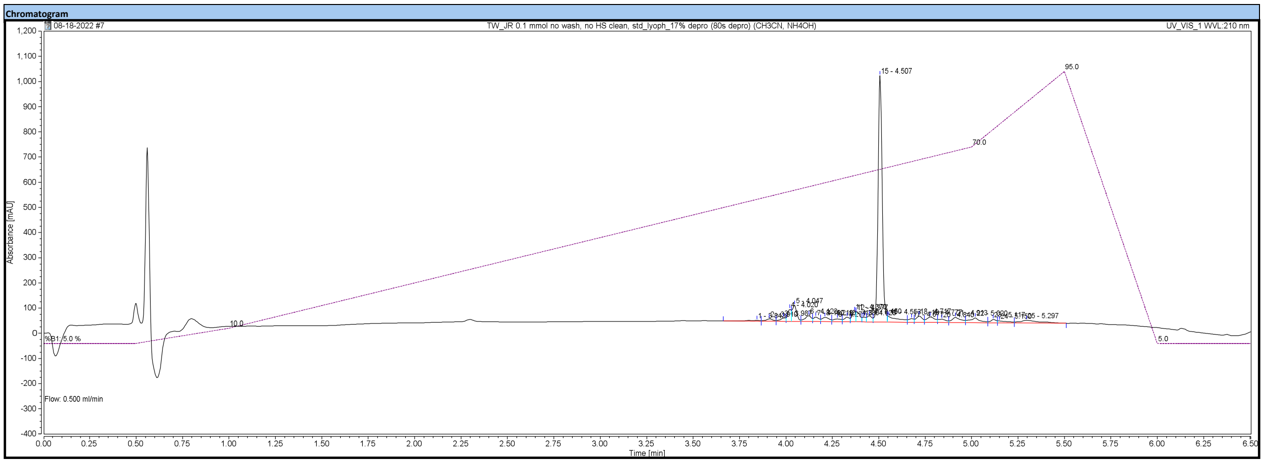

No.	Retention Time	Area	Height	Relative Area	Relative Height
	min	mAU*min	mAU	%	%
UV_VIS_1	UV_VIS_1	UV_VIS_1	UV_VIS_1	UV_VIS_1	UV_VIS_1
1	3.843	0.270	3.030	0.60	0.20
2	3.910	0.421	9.526	0.94	0.64
3	3.987	0.455	13.789	1.01	0.92
4	4.020	1.056	48.692	2.35	3.26
5	4.047	1.794	64.296	3.99	4.30
6	4.123	0.909	23.262	2.02	1.56
7	4.160	0.594	17.814	1.32	1.19
8	4.210	0.750	16.877	1.67	1.13
9	4.277	0.528	10.803	1.17	0.72
10	4.330	0.607	18.490	1.35	1.24
11	4.370	0.907	40.959	2.02	2.74
12	4.377	0.991	38.858	2.20	2.60
13	4.420	0.425	19.143	0.94	1.28
14	4.450	0.744	24.798	1.65	1.66
15	4.507	25.358	978.391	56.39	65.43
16	4.553	1.478	22.551	3.29	1.51
17	4.677	0.441	13.802	0.98	0.92
18	4.717	0.978	25.181	2.17	1.68
19	4.777	1.096	21.265	2.44	1.42
20	4.840	0.612	12.512	1.36	0.84
21	4.913	1.134	20.232	2.52	1.35
22	5.020	1.181	19.055	2.63	1.27
23	5.117	0.463	13.541	1.03	0.91
24	5.150	0.474	7.741	1.05	0.52
25	5.297	1.307	10.634	2.91	0.71
Total:		44.972	1495.242	100.00	100.00

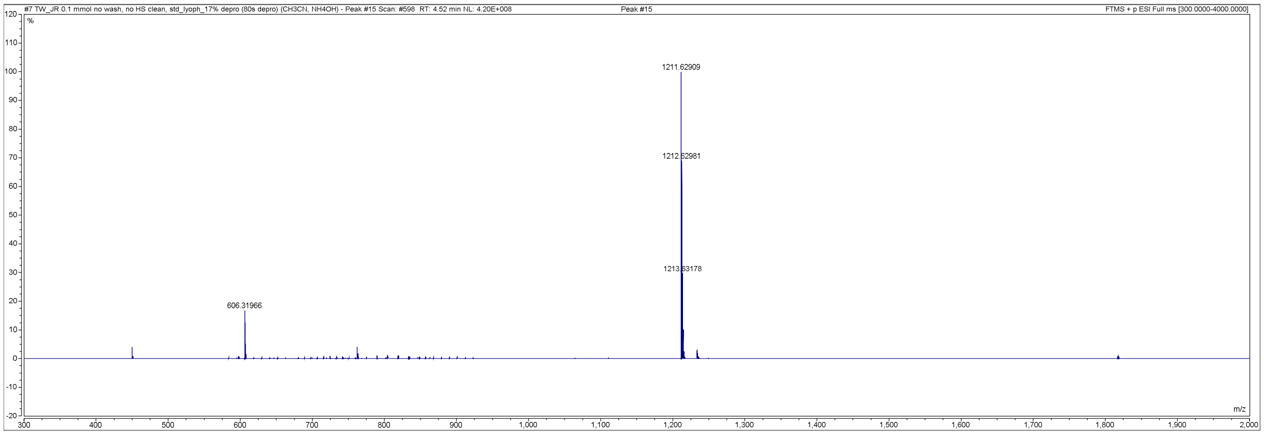

JR-Table 1, entry 10

Chromatogram and Results

Injection Details

Injection Name:	TW_JR complete no wash, std cond_lyophilized_17% depro, 80s depro, 10s drain (NH4OH added)	Run Time (min):	6.50
Vial Number:	Y:C8	Injection Volume:	10.00
Injection Type:	Unknown	Channel:	UV_VIS_1
Calibration Level:		Wavelength:	210
Instrument Method:	ACN10-70_0.500uL_40C Waters Acquity BEH C8 1.7um. 2.1 x 100mm Column	Bandwidth:	4
Processing Method:	Quantitative	Dilution Factor:	1.0000
Injection Date/Time:	17/Aug/22 12:12	Sample Weight:	1.0000

Chromatogram

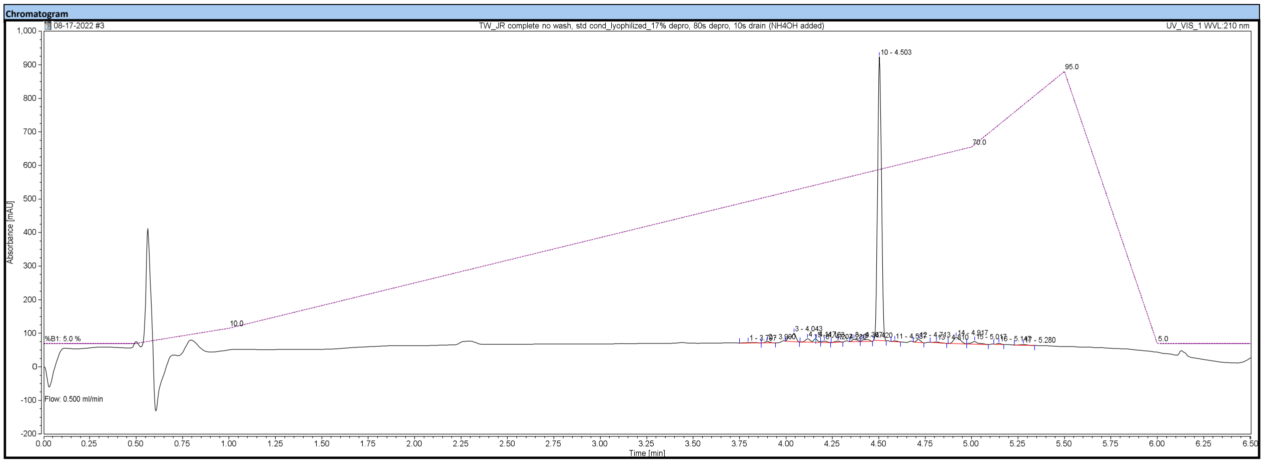

No.	Retention Time	Area	Height	Relative Area	Relative Height
	min	mAU*min	mAU	%	%
UV_VIS_1	UV_VIS_1	UV_VIS_1	UV_VIS_1	UV_VIS_1	UV_VIS_1
1	3.797	0.073	1.287	0.30	0.13
2	3.900	0.119	4.151	0.49	0.43
3	4.043	0.892	25.154	3.66	2.62
4	4.117	0.423	10.081	1.74	1.05
5	4.163	0.132	9.312	0.54	0.97
6	4.207	0.146	4.372	0.60	0.45
7	4.280	0.104	3.176	0.43	0.33
8	4.367	0.226	7.029	0.93	0.73
9	4.420	0.250	5.855	1.03	0.61
10	4.503	20.381	845.103	83.71	87.90
11	4.587	0.069	2.766	0.29	0.29
12	4.713	0.212	8.985	0.87	0.93
13	4.810	0.066	2.246	0.27	0.23
14	4.917	0.794	19.032	3.26	1.98
15	5.017	0.330	8.060	1.35	0.84
16	5.147	0.055	2.771	0.23	0.29
17	5.280	0.076	2.074	0.31	0.22
Total:		24.349	961.454	100.00	100.00

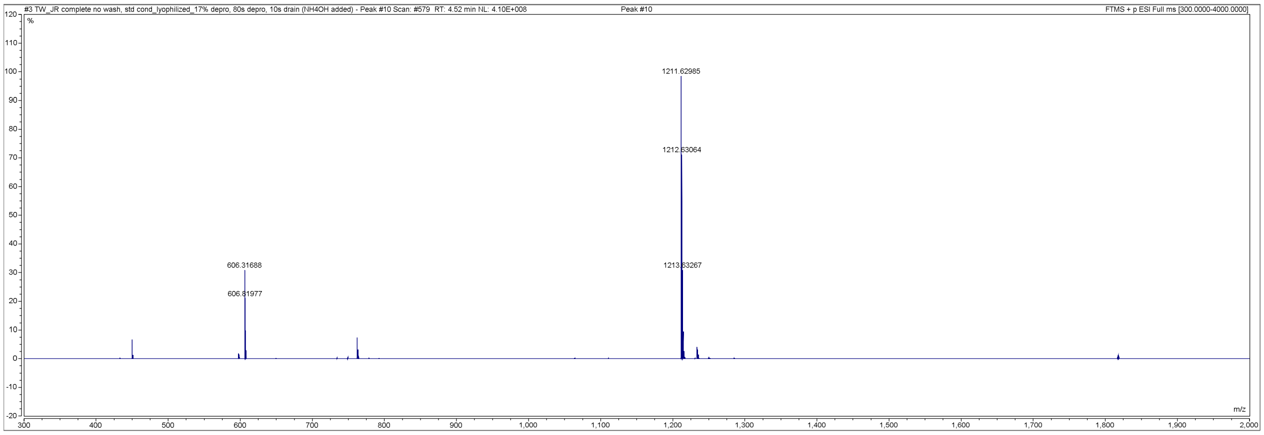

JR-Table 1, entry 11

Chromatogram and Results

Injection Details

Injection Name:	*TW_JR no wash, std cond_lyoph_RA MBHA resin (17% depro, 80s depro)*		*Run Time (min):*	*6.50*
Vial Number:	*Y:A1*		*Injection Volume:*	*18.00*
Injection Type:	*Unknown*		*Channel:*	*UV_VIS_1*
Calibration Level:			*Wavelength:*	*210*
Instrument Method:	*ACN10-70_0.500uL_40C*	*Waters Acquity BEH C8 1.7um. 2.1 x 100mm Column*	*Bandwidth:*	*4*
Processing Method:	*Quantitative*		*Dilution Factor:*	*1.0000*
Injection Date/Time:	*23/Aug/22 11:58*		*Sample Weight:*	*1.0000*

Chromatogram

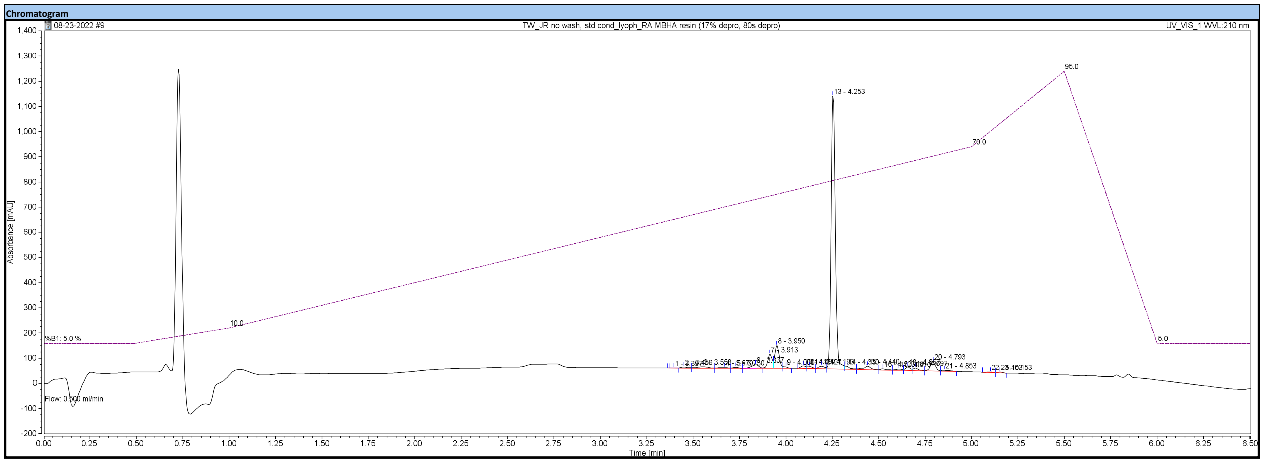

No.	Retention Time	Area	Height	Relative Area	Relative Height
	min	mAU*min	mAU	%	%
UV_VIS_1	UV_VIS_1	UV_VIS_1	UV_VIS_1	UV_VIS_1	UV_VIS_1
1	3.397	0.012	0.510	0.03	0.04
2	3.450	0.226	4.618	0.58	0.33
3	3.553	0.414	5.569	1.06	0.40
4	3.670	0.150	2.316	0.38	0.17
5	3.730	0.125	4.157	0.32	0.30
6	3.837	0.564	13.490	1.45	0.97
7	3.913	1.618	55.758	4.16	3.99
8	3.950	2.458	90.169	6.31	6.45
9	4.000	0.172	5.945	0.44	0.43
10	4.097	0.309	9.872	0.79	0.71
11	4.127	0.237	7.509	0.61	0.54
12	4.193	0.374	10.596	0.96	0.76
13	4.253	28.598	1084.314	73.46	77.57
14	4.330	0.386	11.374	0.99	0.81
15	4.440	0.646	13.663	1.66	0.98
16	4.523	0.175	4.495	0.45	0.32
17	4.610	0.198	4.714	0.51	0.34
18	4.657	0.404	14.042	1.04	1.00
19	4.697	0.329	10.395	0.85	0.74
20	4.793	1.184	36.172	3.04	2.59
21	4.853	0.179	3.264	0.46	0.23
22	5.103	0.077	1.895	0.20	0.14
23	5.153	0.092	2.959	0.24	0.21
Total:		38.929	1397.796	100.00	100.00

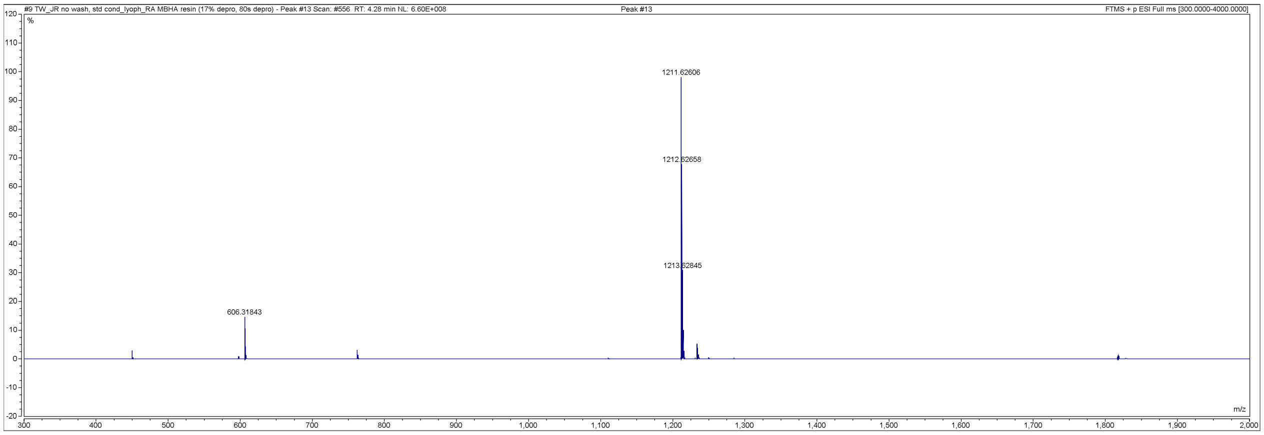

Chromeleon (c) Dionex
Version 7.2.10.23925

JR-Table 1, entry 12

Chromatogram and Results

Injection Details

Injection Name:	TW_JR no wash (7/26) std cond_lyoph_40s depro		Run Time (min):	6.50
Vial Number:	B:A6		Injection Volume:	5.00
Injection Type:	Unknown		Channel:	UV_VIS_1
Calibration Level:			Wavelength:	210
Instrument Method:	ACN10-70_0.500uL_40C	Waters Acquity BEH C8 1.7um. 2.1 x 100mm Column	Bandwidth:	4
Processing Method:	Quantitative		Dilution Factor:	1.0000
Injection Date/Time:	06/Dec/22 10:38		Sample Weight:	1.0000

Chromatogram

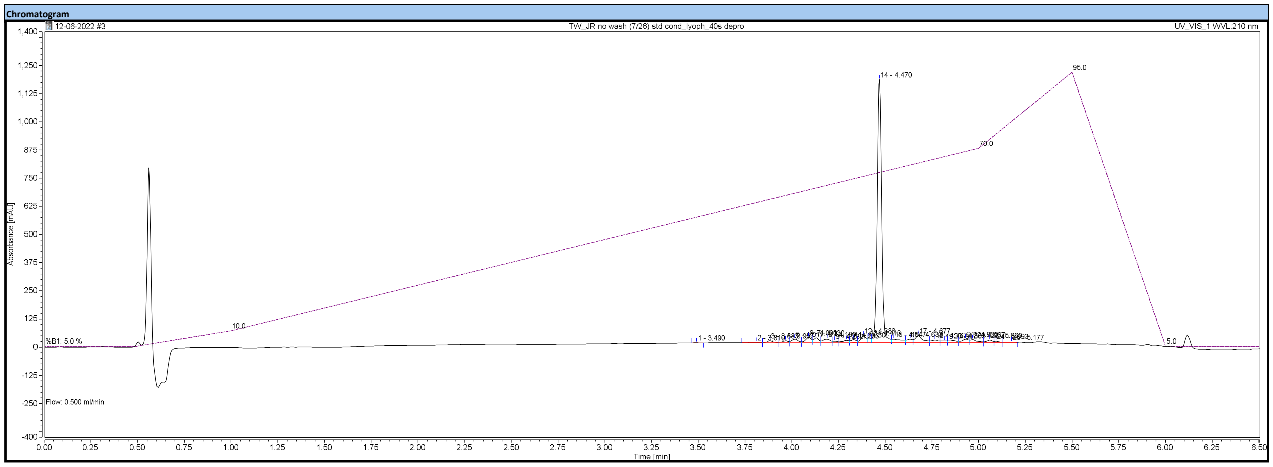

No.	Retention Time	Area	Height	Relative Area	Relative Height
	min	mAU*min	mAU	%	%
UV_VIS_1	UV_VIS_1	UV_VIS_1	UV_VIS_1	UV_VIS_1	UV_VIS_1
1	3.490	0.034	1.235	0.08	0.08
2	3.810	0.113	1.831	0.26	0.12
3	3.883	0.306	8.409	0.70	0.57
4	3.967	0.297	8.543	0.68	0.58
5	4.017	0.616	15.639	1.41	1.06
6	4.090	0.826	22.910	1.90	1.56
7	4.130	0.644	21.591	1.48	1.47
8	4.190	0.693	15.081	1.59	1.02
9	4.230	0.170	6.184	0.39	0.42
10	4.293	0.386	9.659	0.89	0.66
11	4.330	0.421	12.919	0.97	0.88
12	4.383	0.958	30.942	2.20	2.10
13	4.413	0.465	21.775	1.07	1.48
14	4.470	32.077	1166.285	73.69	79.25
15	4.547	0.924	14.631	2.12	0.99
16	4.633	0.499	14.534	1.15	0.99
17	4.677	1.364	30.939	3.13	2.10
18	4.767	0.467	10.709	1.07	0.73
19	4.817	0.260	6.842	0.60	0.46
20	4.863	0.407	8.328	0.94	0.57
21	4.930	0.577	14.254	1.32	0.97
22	4.967	0.488	12.198	1.12	0.83
23	5.060	0.354	10.275	0.81	0.70
24	5.093	0.127	4.837	0.29	0.33
25	5.177	0.057	1.117	0.13	0.08
Total:		43.529	1471.666	100.00	100.00

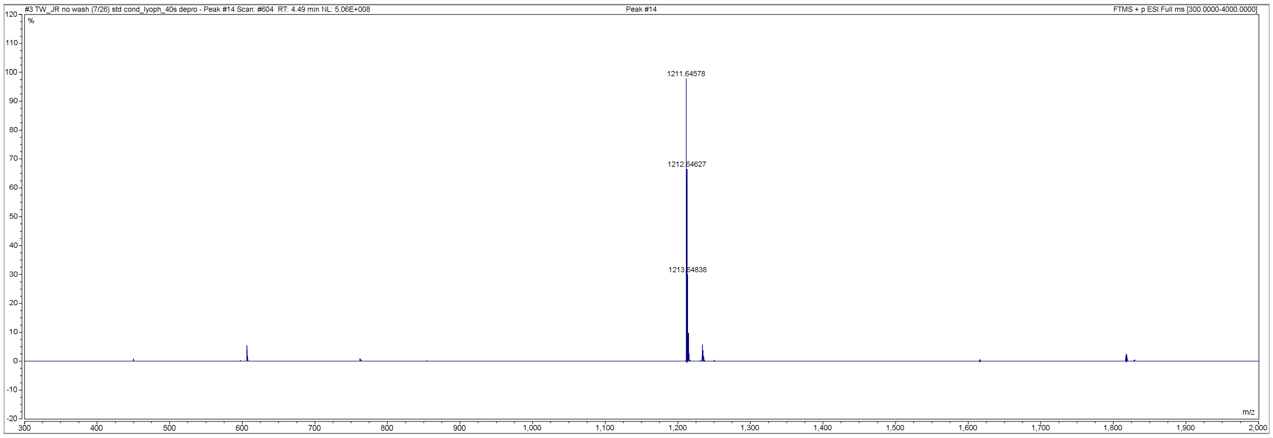

Chromeleon (c) Dionex
Version 7.2.10.23925

JR-Table 1, entry 13

Chromatogram and Results

Injection Details

Injection Name:	TW_JR 0.1 mmol no wash, no HS clean, std_lyoph_12% depro (80s depro) (CH3CN, NH4OH)	Run Time (min):	6.50
Vial Number:	Y:A3	Injection Volume:	25.00
Injection Type:	Unknown	Channel:	UV_VIS_1
Calibration Level:		Wavelength:	210
Instrument Method:	ACN10-70_0.500uL_40C Waters Acquity BEH C8 1.7um. 2.1 x 100mm Column	Bandwidth:	4
Processing Method:	Quantitative	Dilution Factor:	1.0000
Injection Date/Time:	18/Aug/22 11:31	Sample Weight:	1.0000

Chromatogram

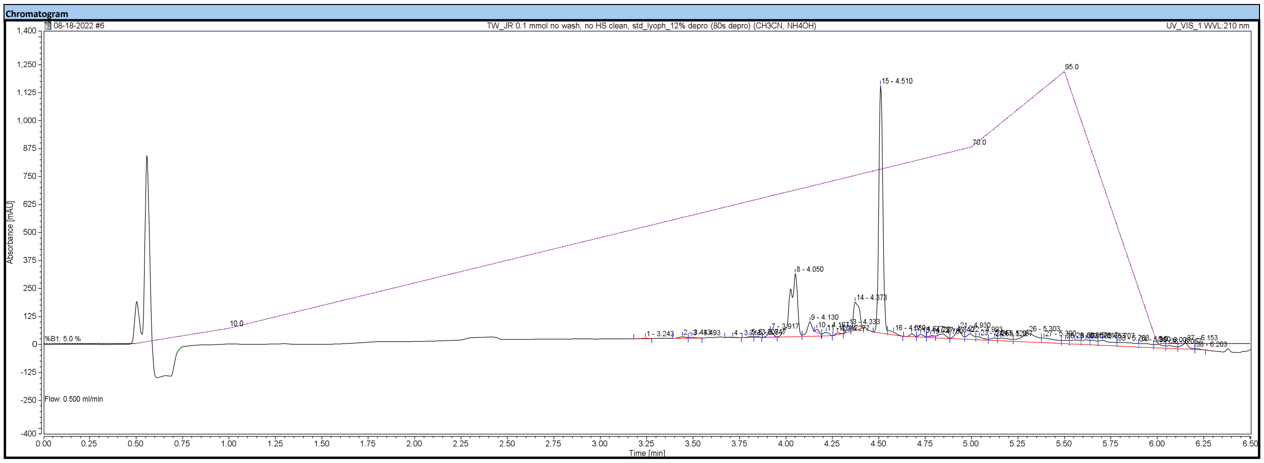

No.	Retention Time	Area	Height	Relative Area	Relative Height
	min	mAU*min	mAU	%	%
UV_VIS_1	UV_VIS_1	UV_VIS_1	UV_VIS_1	UV_VIS_1	UV_VIS_1
1	3.243	0.065	1.514	0.09	0.07
2	3.443	0.315	6.501	0.42	0.31
3	3.493	0.155	3.885	0.20	0.18
4	3.713	0.105	2.083	0.14	0.10
5	3.807	0.150	5.026	0.20	0.24
6	3.847	0.119	3.651	0.16	0.17
7	3.917	0.867	28.257	1.15	1.34
8	4.050	13.098	281.245	17.30	13.34
9	4.130	2.667	64.962	3.52	3.08
10	4.167	0.247	13.679	0.33	0.65
11	4.217	0.564	15.437	0.75	0.73
12	4.277	0.234	8.776	0.31	0.42
13	4.333	0.529	26.504	0.70	1.26
14	4.373	4.592	125.311	6.06	5.94
15	4.510	27.852	1106.878	36.78	52.49
16	4.580	0.773	12.849	1.02	0.61
17	4.677	0.380	14.468	0.50	0.69
18	4.727	0.378	11.919	0.50	0.57
19	4.780	0.073	2.830	0.10	0.13
20	4.840	1.184	18.729	1.56	0.89
21	4.930	1.591	41.554	2.10	1.97
22	4.993	1.058	25.333	1.40	1.20
23	5.043	0.599	12.642	0.79	0.60
24	5.123	0.428	11.365	0.57	0.54
25	5.157	0.865	12.253	1.14	0.58
26	5.303	3.671	38.109	4.85	1.81
27	5.390	1.826	21.071	2.41	1.00
28	5.507	0.614	14.755	0.81	0.70
29	5.557	1.099	18.265	1.45	0.87
30	5.617	0.935	21.813	1.23	1.03
31	5.653	0.838	20.883	1.11	0.99
32	5.707	1.854	23.208	2.45	1.10
33	5.783	1.889	15.641	2.49	0.74
34	5.900	1.230	15.198	1.62	0.72
35	6.003	0.873	15.962	1.15	0.76
36	6.067	0.643	12.624	0.85	0.60
37	6.153	1.238	30.030	1.63	1.42
38	6.203	0.125	3.383	0.17	0.16
Total:		75.722	2108.590	100.00	100.00

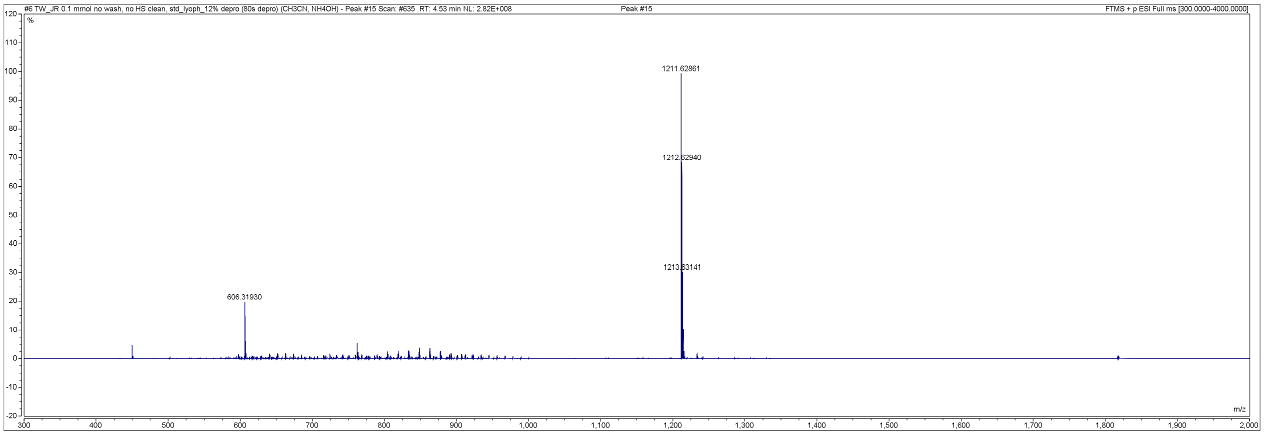

JR-Table 1, entry 14

Chromatogram and Results

Injection Details

Injection Name:	TW_JR 0.1 mmol no wash, std_lyoph_12% depro (80s depro) (CH3CN, NH4OH)	Run Time (min):	6.50
Vial Number:	Y:C1	Injection Volume:	5.00
Injection Type:	Unknown	Channel:	UV_VIS_1
Calibration Level:		Wavelength:	210
Instrument Method:	ACN10-70_0.500uL_40C Waters Acquity BEH C8 1.7um. 2.1 x 100mm Column	Bandwidth:	4
Processing Method:	Quantitative	Dilution Factor:	1.0000
Injection Date/Time:	16/Aug/22 14:38	Sample Weight:	1.0000

Chromatogram

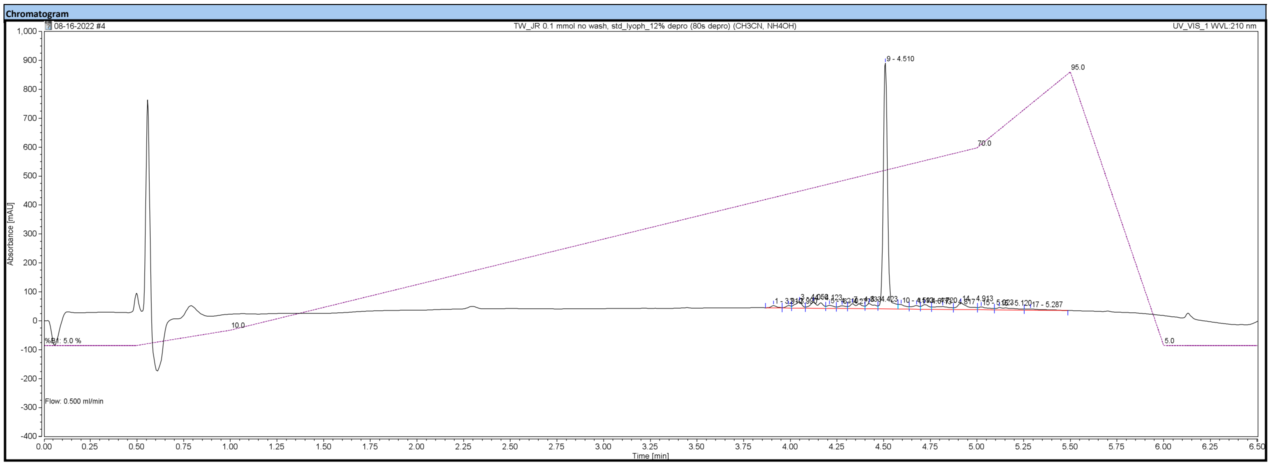

No.	Retention Time	Area	Height	Relative Area	Relative Height
	min	mAU*min	mAU	%	%
UV_VIS_1	UV_VIS_1	UV_VIS_1	UV_VIS_1	UV_VIS_1	UV_VIS_1
1	3.910	0.306	8.328	0.88	0.78
2	3.990	0.265	8.653	0.76	0.81
3	4.050	0.934	22.601	2.68	2.13
4	4.123	1.360	20.628	3.90	1.94
5	4.210	0.463	10.458	1.33	0.98
6	4.277	0.450	9.115	1.29	0.86
7	4.333	1.137	16.743	3.26	1.58
8	4.423	0.906	18.168	2.60	1.71
9	4.510	22.533	848.793	64.59	79.93
10	4.593	0.726	15.204	2.08	1.43
11	4.677	0.605	12.673	1.73	1.19
12	4.720	0.720	16.104	2.06	1.52
13	4.817	0.944	9.875	2.71	0.93
14	4.913	1.538	22.295	4.41	2.10
15	5.023	0.610	9.680	1.75	0.91
16	5.120	0.887	8.497	2.54	0.80
17	5.287	0.501	4.132	1.44	0.39
Total:		34.883	1061.946	100.00	100.00

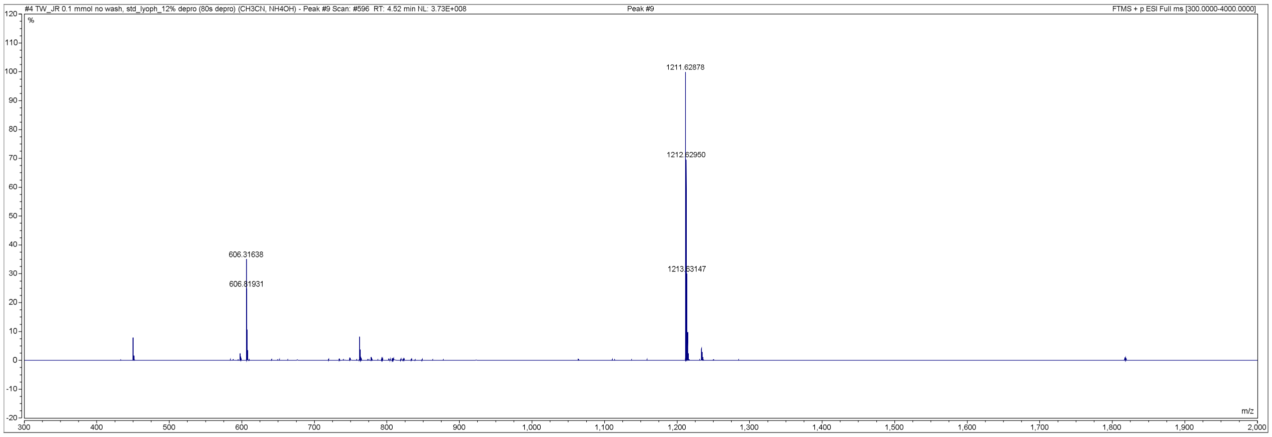

JR-Table 1, entry 15

Chromatogram and Results

Injection Details

Injection Name:	*TW_JR WASH 0.1 mmol PRIME_lyoph_80s 17perc depro (1/26/23)*	*Run Time (min):*	*6.50*
Vial Number:	*B:A1*	*Injection Volume:*	*14.00*
Injection Type:	*Unknown*	*Channel:*	*UV_VIS_1*
Calibration Level:		*Wavelength:*	*210*
Instrument Method:	*ACN10-70_0.500uL_40C* *Waters Acquity BEH C8 1.7um. 2.1 x 100mm Column*	*Bandwidth:*	*4*
Processing Method:	*Quantitative*	*Dilution Factor:*	*1.0000*
Injection Date/Time:	*30/Jan/23 16:12*	*Sample Weight:*	*1.0000*

Chromatogram

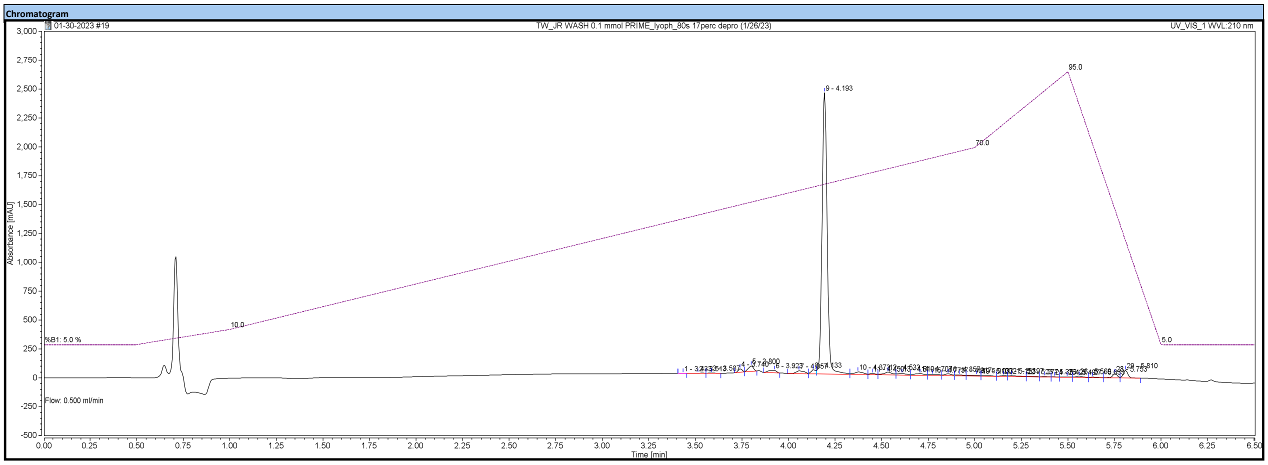

No.	Retention Time	Area	Height	Relative Area	Relative Height
	min	mAU*min	mAU	%	%
UV_VIS_1	UV_VIS_1	UV_VIS_1	UV_VIS_1	UV_VIS_1	UV_VIS_1
1	3.433	0.009	0.388	0.01	0.01
2	3.513	0.155	1.806	0.16	0.06
3	3.587	0.154	3.681	0.16	0.13
4	3.740	0.735	29.846	0.77	1.04
5	3.800	1.522	46.333	1.60	1.61
6	3.923	0.992	20.568	1.04	0.72
7	4.057	1.317	25.498	1.38	0.89
8	4.133	1.033	36.483	1.08	1.27
9	4.193	77.116	2436.467	80.94	84.86
10	4.373	1.281	22.214	1.34	0.77
11	4.450	0.381	10.242	0.40	0.36
12	4.533	1.254	24.290	1.32	0.85
13	4.610	0.792	13.037	0.83	0.45
14	4.707	0.915	14.604	0.96	0.51
15	4.773	0.526	7.550	0.55	0.26
16	4.857	0.696	16.630	0.73	0.58
17	4.917	0.416	6.868	0.44	0.24
18	5.010	0.483	6.307	0.51	0.22
19	5.033	0.387	5.485	0.41	0.19
20	5.153	0.307	7.381	0.32	0.26
21	5.197	0.474	7.260	0.50	0.25
22	5.277	0.195	2.410	0.20	0.08
23	5.373	0.203	3.890	0.21	0.14
24	5.427	0.097	2.421	0.10	0.08
25	5.497	0.141	2.467	0.15	0.09
26	5.560	0.459	10.582	0.48	0.37
27	5.633	0.228	4.151	0.24	0.14
28	5.753	1.142	37.152	1.20	1.29
29	5.810	1.871	65.089	1.96	2.27
Total:		95.280	2871.096	100.00	100.00

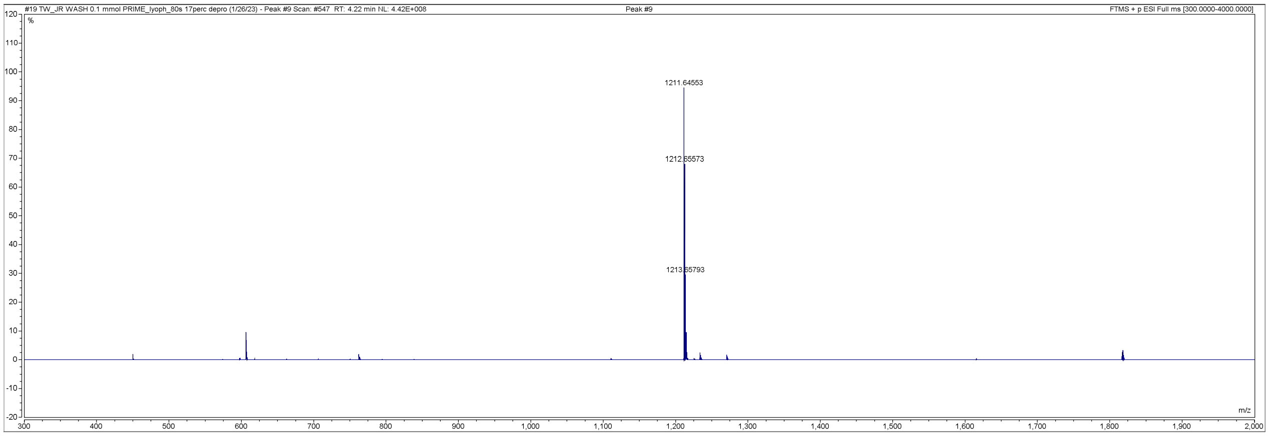

Chromeleon (c) Dionex
Version 7.2.10.23925

JR-Table 1, entry 16

Chromatogram and Results

Injection Details

Injection Name:	*TW_JR WASH, all reagents in NBP_PRIME_lyoph_40s depro, 25% depro (9/15)*	*Run Time (min):*	*6.50*
Vial Number:	*B:A4*	*Injection Volume:*	*10.00*
Injection Type:	*Unknown*	*Channel:*	*UV_VIS_1*
Calibration Level:		*Wavelength:*	*210*
Instrument Method:	*ACN10-70_0.500uL_40C* *Waters Acquity BEH C8 1.7um. 2.1 x 100mm Column*	*Bandwidth:*	*4*
Processing Method:	*Quantitative*	*Dilution Factor:*	*1.0000*
Injection Date/Time:	*02/Feb/23 16:00*	*Sample Weight:*	*1.0000*

Chromatogram

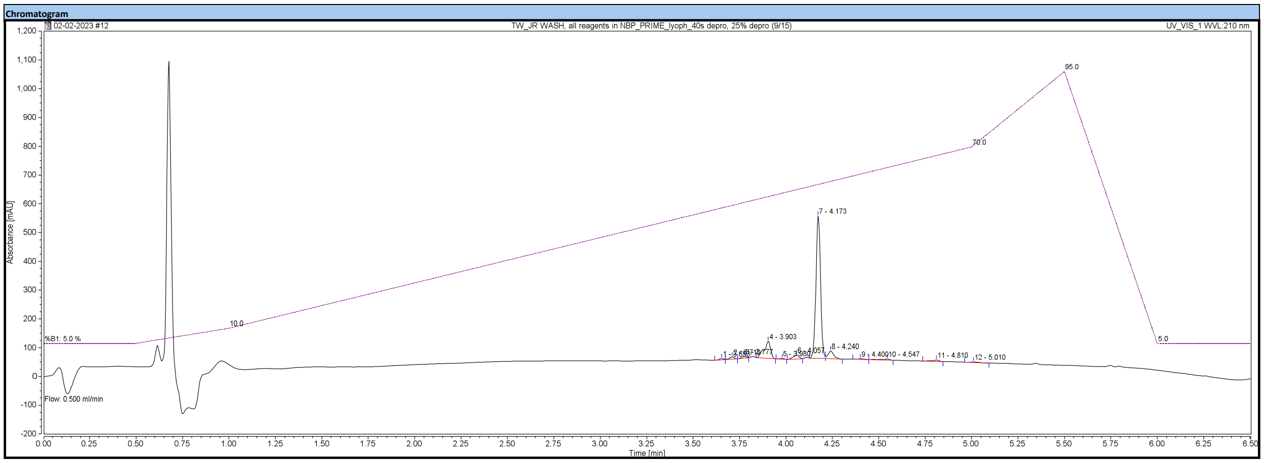

No.	Retention Time	Area	Height	Relative Area	Relative Height
	min	mAU*min	mAU	%	%
UV_VIS_1	UV_VIS_1	UV_VIS_1	UV_VIS_1	UV_VIS_1	UV_VIS_1
1	3.653	0.120	4.341	0.62	0.69
2	3.710	0.229	7.848	1.19	1.25
3	3.777	0.184	6.580	0.95	1.05
4	3.903	2.397	59.728	12.41	9.49
5	3.980	0.070	2.837	0.36	0.45
6	4.057	0.493	13.708	2.55	2.18
7	4.173	14.289	494.996	73.96	78.63
8	4.240	0.946	26.874	4.90	4.27
9	4.400	0.088	1.769	0.45	0.28
10	4.547	0.218	4.187	1.13	0.67
11	4.810	0.192	5.112	1.00	0.81
12	5.010	0.094	1.526	0.49	0.24
Total:		19.320	629.507	100.00	100.00

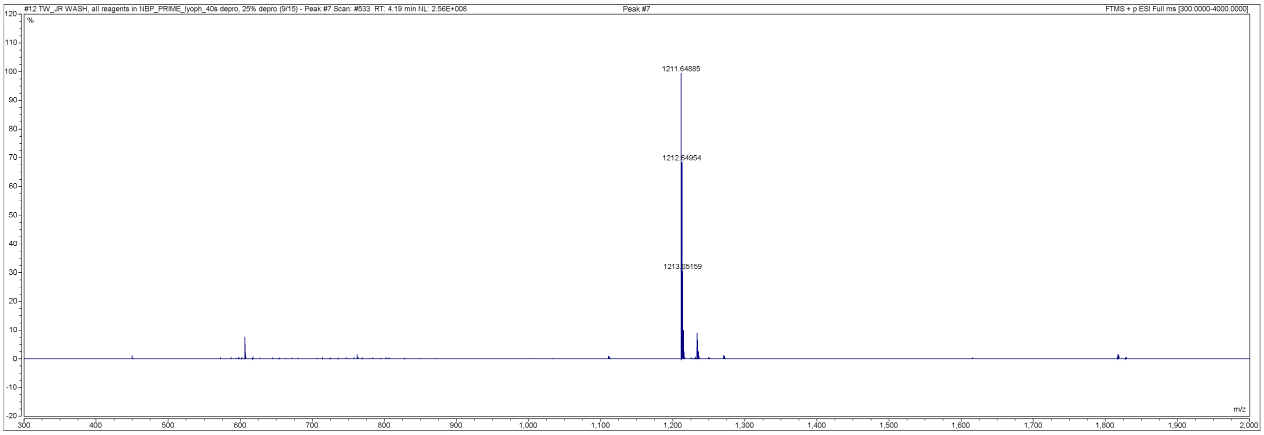

JR-Table 1, entry 17

Chromatogram and Results

Injection Details

Injection Name:	TW_JR no wash, all reagents in NBP_lyoph_80s depro, 17% depro (9/14)	Run Time (min):	6.50
Vial Number:	B:A5	Injection Volume:	15.00
Injection Type:	Unknown	Channel:	UV_VIS_1
Calibration Level:		Wavelength:	210
Instrument Method:	ACN10-70_0.500uL_40C Waters Acquity BEH C8 1.7um. 2.1 x 100mm Column	Bandwidth:	4
Processing Method:	Quantitative	Dilution Factor:	1.0000
Injection Date/Time:	03/Feb/23 08:40	Sample Weight:	1.0000

Chromatogram

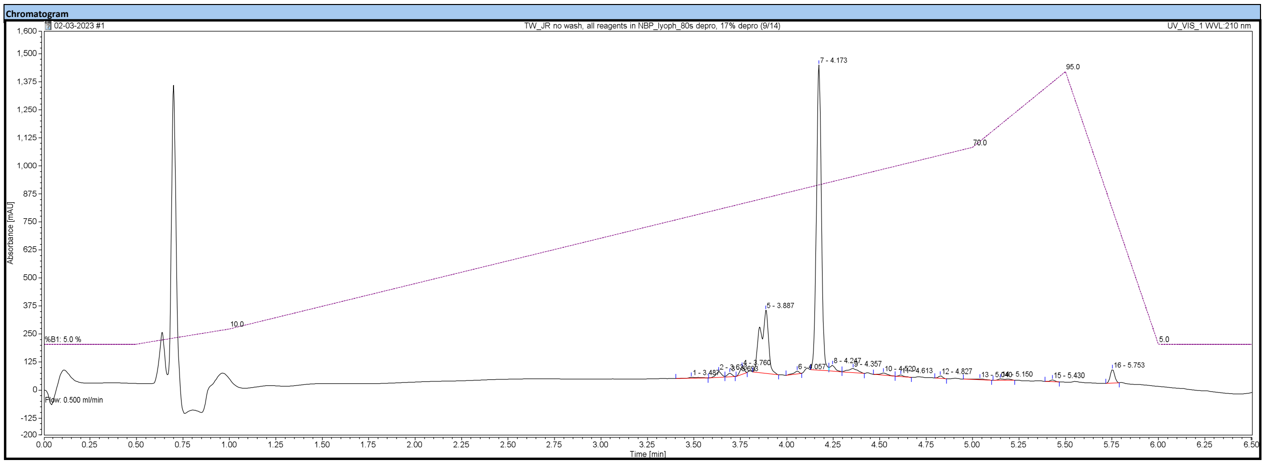

No.	Retention Time	Area	Height	Relative Area	Relative Height
	min	mAU*min	mAU	%	%
UV_VIS_1	UV_VIS_1	UV_VIS_1	UV_VIS_1	UV_VIS_1	UV_VIS_1
1	3.487	0.326	4.500	0.50	0.24
2	3.633	0.903	25.699	1.39	1.37
3	3.693	0.461	16.279	0.71	0.87
4	3.760	0.776	30.411	1.20	1.62
5	3.887	14.569	281.506	22.49	14.97
6	4.057	0.422	12.753	0.65	0.68
7	4.173	41.940	1361.068	64.74	72.39
8	4.247	0.855	25.490	1.32	1.36
9	4.357	1.097	18.055	1.69	0.96
10	4.520	0.418	9.583	0.65	0.51
11	4.613	0.205	6.547	0.32	0.35
12	4.827	0.292	10.962	0.45	0.58
13	5.040	0.269	3.486	0.42	0.19
14	5.150	0.354	6.096	0.55	0.32
15	5.430	0.219	7.913	0.34	0.42
16	5.753	1.677	59.962	2.59	3.19
Total:		64.784	1880.307	100.00	100.00

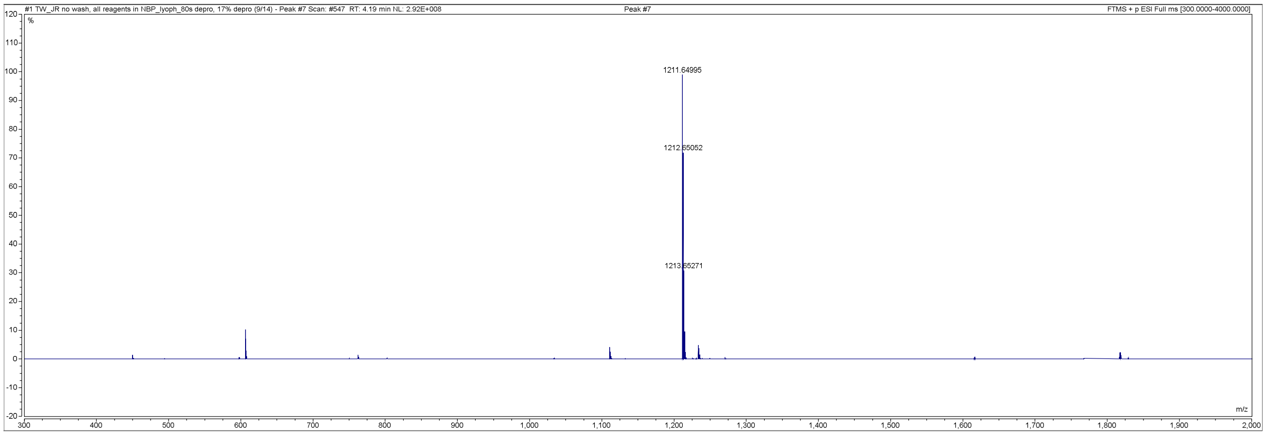

JR-Table 1, entry 18

Chromatogram and Results

Injection Details

Injection Name:	*SS-JR-NMP-25%pyrrolidine-40 sec depro-std PRIME-wash-lyo*	*Run Time (min):*	*6.50*
Vial Number:	*G:F5*	*Injection Volume:*	*10.00*
Injection Type:	*Unknown*	*Channel:*	*UV_VIS_1*
Calibration Level:		*Wavelength:*	*210*
Instrument Method:	*ACN10-70_0.500uL_40C* *Waters Acquity BEH C8 1.7um. 2.1 x 100mm Column*	*Bandwidth:*	*4*
Processing Method:	*Quantitative*	*Dilution Factor:*	*1.0000*
Injection Date/Time:	*09/Aug/23 11:24*	*Sample Weight:*	*1.0000*

Chromatogram

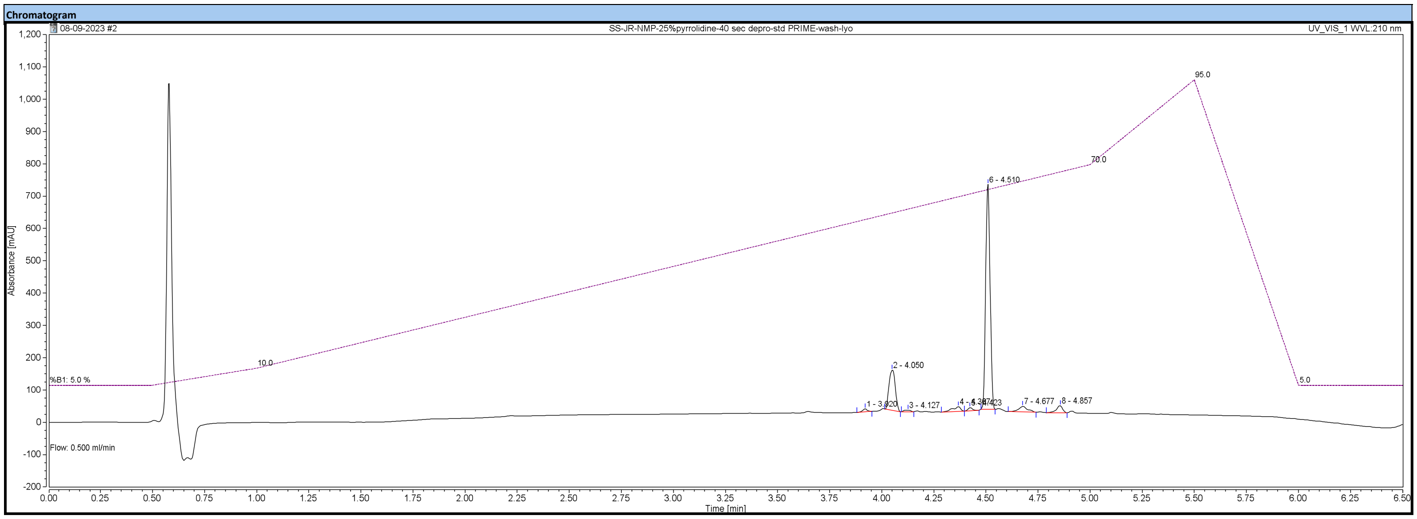

No.	Retention Time	Area	Height	Relative Area	Relative Height
	min	mAU*min	mAU	%	%
UV_VIS_1	UV_VIS_1	UV_VIS_1	UV_VIS_1	UV_VIS_1	UV_VIS_1
1	3.920	0.245	9.301	1.00	1.04
2	4.050	4.345	124.242	17.81	13.85
3	4.127	0.189	4.994	0.78	0.56
4	4.367	0.653	14.334	2.68	1.60
5	4.423	0.246	9.510	1.01	1.06
6	4.510	17.105	695.162	70.12	77.52
7	4.677	0.869	17.473	3.56	1.95
8	4.857	0.741	21.770	3.04	2.43
Total:		24.392	896.786	100.00	100.00

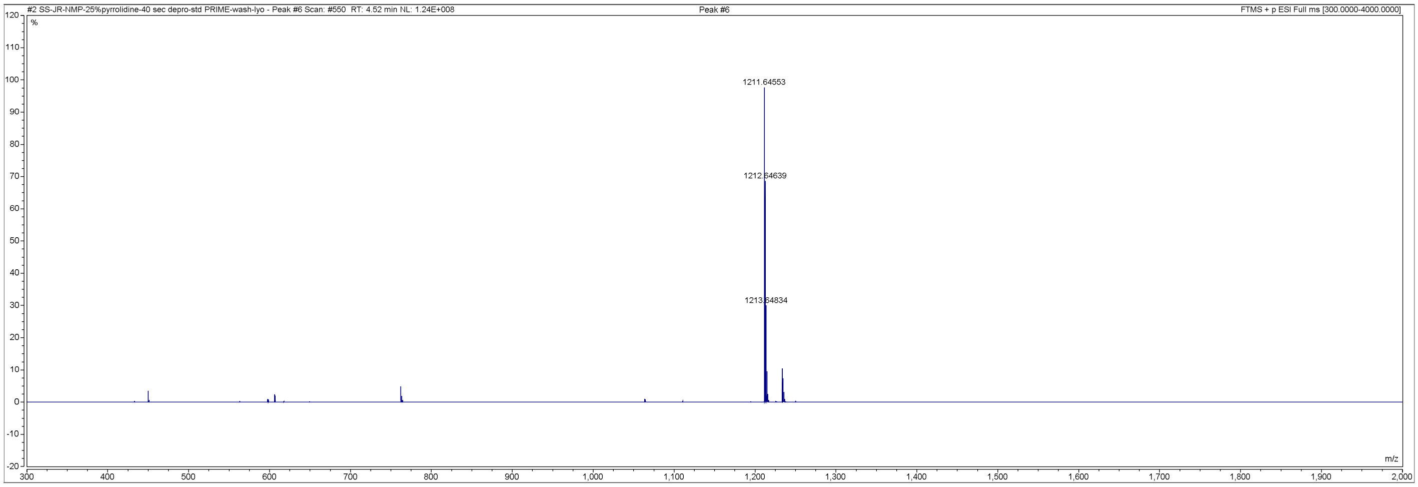

Chromeleon (c) Dionex
Version 7.2.10.23925

JR-Table 1, entry 19

Chromatogram and Results

Injection Details

Injection Name:	TW_JR no wash, NMP solvent_lyoph_80s depro, 17% depro (NH4OH add)	Run Time (min):	6.50
Vial Number:	Y:B5	Injection Volume:	4.00
Injection Type:	Unknown	Channel:	UV_VIS_1
Calibration Level:		Wavelength:	210
Instrument Method:	ACN10-70_0.500uL_40C Waters Acquity BEH C8 1.7um. 2.1 x 100mm Column	Bandwidth:	4
Processing Method:	Quantitative	Dilution Factor:	1.0000
Injection Date/Time:	25/Oct/22 12:12	Sample Weight:	1.0000

Chromatogram

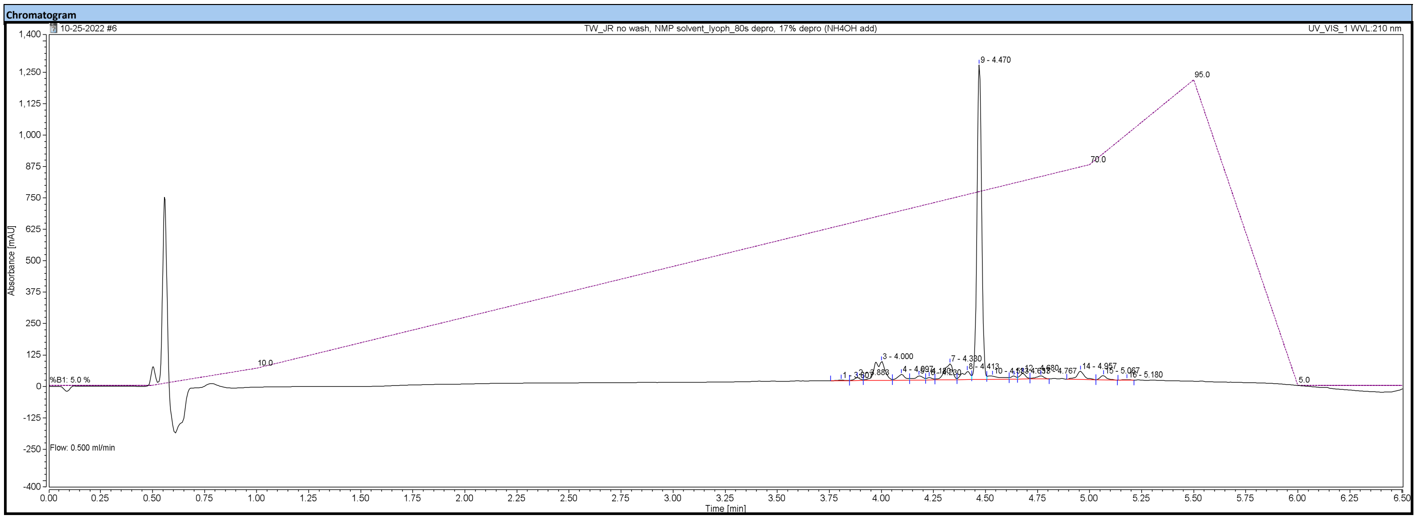

No.	Retention Time	Area	Height	Relative Area	Relative Height
	min	mAU*min	mAU	%	%
UV_VIS_1	UV_VIS_1	UV_VIS_1	UV_VIS_1	UV_VIS_1	UV_VIS_1
1	3.807	0.150	2.841	0.31	0.18
2	3.883	0.410	12.353	0.84	0.77
3	4.000	4.459	75.131	9.17	4.69
4	4.097	0.960	23.089	1.97	1.44
5	4.180	0.770	15.997	1.58	1.00
6	4.230	0.298	9.802	0.61	0.61
7	4.330	2.919	63.525	6.00	3.97
8	4.413	1.476	32.593	3.03	2.04
9	4.470	32.406	1251.204	66.61	78.18
10	4.533	1.003	13.055	2.06	0.82
11	4.637	0.376	12.471	0.77	0.78
12	4.680	0.752	23.307	1.55	1.46
13	4.767	0.541	12.106	1.11	0.76
14	4.957	1.435	33.868	2.95	2.12
15	5.067	0.620	17.085	1.27	1.07
16	5.180	0.076	1.938	0.16	0.12
Total:		48.649	1600.366	100.00	100.00

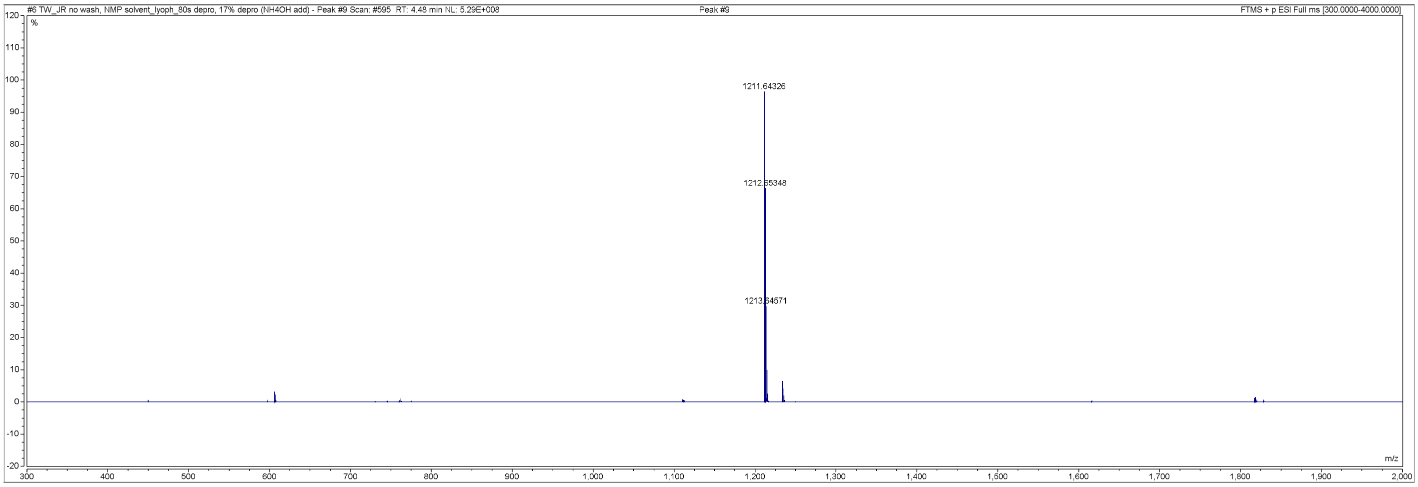

Chromeleon (c) Dionex
Version 7.2.10.23925

JR-Table 1, entry 20

Chromatogram and Results

Injection Details

Injection Name:	*SS-JR-25%pipd-std PRIME-wash-lyo*		*Run Time (min):*	*6.50*
Vial Number:	*G:F8*		*Injection Volume:*	*10.00*
Injection Type:	*Unknown*		*Channel:*	*UV_VIS_1*
Calibration Level:			*Wavelength:*	*210*
Instrument Method:	*ACN10-70_0.500uL_40C*	*Waters Acquity BEH C8 1.7um. 2.1 x 100mm Column*	*Bandwidth:*	*4*
Processing Method:	*Quantitative*		*Dilution Factor:*	*1.0000*
Injection Date/Time:	*04/Aug/23 13:51*		*Sample Weight:*	*1.0000*

Chromatogram

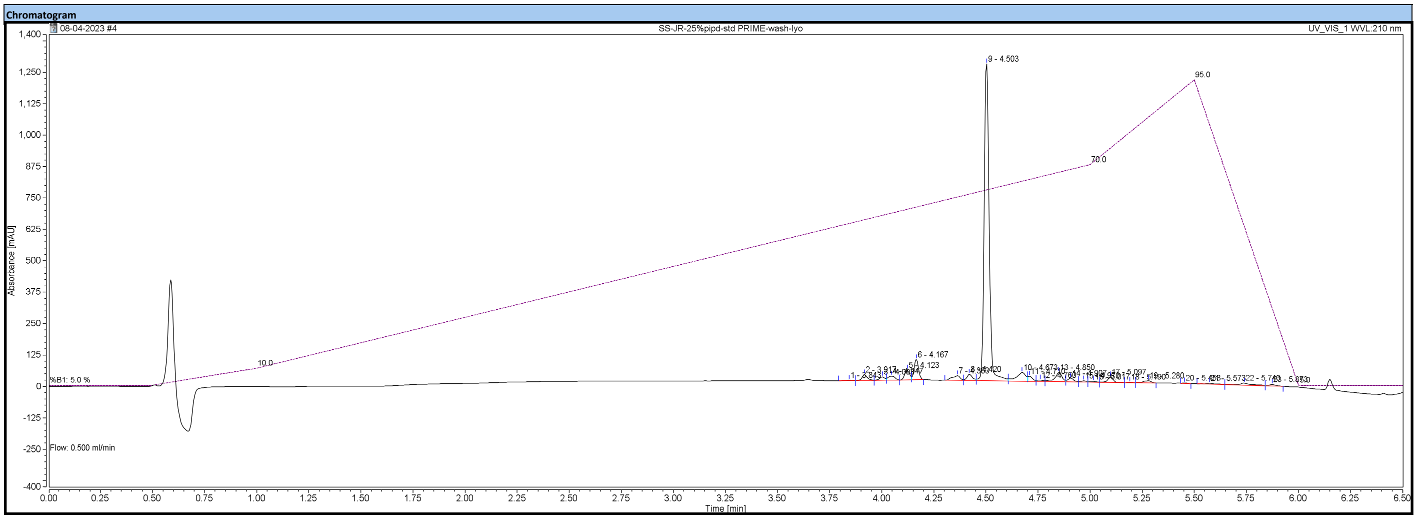

No.	Retention Time	Area	Height	Relative Area	Relative Height
	min	mAU*min	mAU	%	%
UV_VIS_1	UV_VIS_1	UV_VIS_1	UV_VIS_1	UV_VIS_1	UV_VIS_1
1	3.843	0.071	1.834	0.15	0.11
2	3.917	0.671	22.617	1.40	1.37
3	4.003	0.403	13.517	0.84	0.82
4	4.047	0.598	16.173	1.24	0.98
5	4.123	1.076	39.256	2.24	2.38
6	4.167	1.963	79.603	4.09	4.83
7	4.363	0.792	18.217	1.65	1.11
8	4.420	0.748	25.076	1.56	1.52
9	4.503	34.879	1260.708	72.59	76.50
10	4.673	1.793	34.595	3.73	2.10
11	4.710	0.521	20.252	1.08	1.23
12	4.760	0.189	5.495	0.39	0.33
13	4.850	1.456	37.227	3.03	2.26
14	4.907	0.474	14.853	0.99	0.90
15	4.970	0.148	4.536	0.31	0.28
16	5.017	0.115	2.398	0.24	0.15
17	5.097	0.661	22.190	1.38	1.35
18	5.190	0.055	1.974	0.11	0.12
19	5.280	0.393	8.980	0.82	0.54
20	5.453	0.040	1.722	0.08	0.10
21	5.573	0.182	3.025	0.38	0.18
22	5.740	0.588	8.199	1.22	0.50
23	5.873	0.230	5.586	0.48	0.34
Total:		48.048	1648.034	100.00	100.00

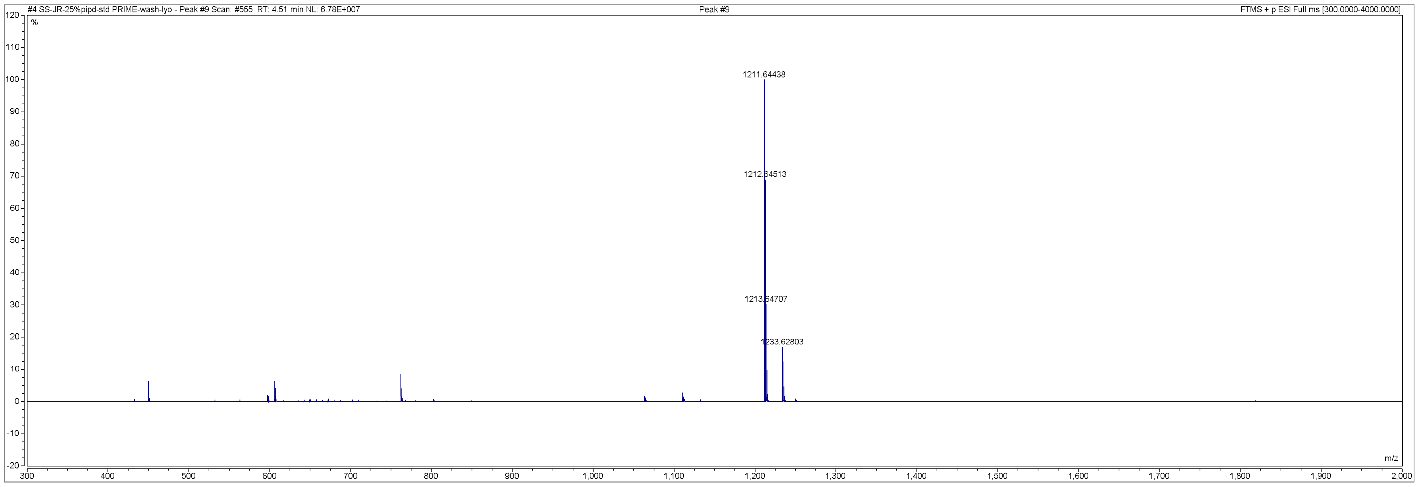

Chromeleon (c) Dionex
Version 7.2.10.23925

JR-Table 1, entry 21

Chromatogram and Results

Injection Details

Injection Name:	*SS-JR-25%pipd-std PRIME-no wash-lyo*		*Run Time (min):*	*6.50*
Vial Number:	*G:F9*		*Injection Volume:*	*10.00*
Injection Type:	*Unknown*		*Channel:*	*UV_VIS_1*
Calibration Level:			*Wavelength:*	*210*
Instrument Method:	*ACN10-70_0.500uL_40C*	*Waters Acquity BEH C8 1.7um. 2.1 x 100mm Column*	*Bandwidth:*	*4*
Processing Method:	*Quantitative*		*Dilution Factor:*	*1.0000*
Injection Date/Time:	*04/Aug/23 13:58*		*Sample Weight:*	*1.0000*

Chromatogram

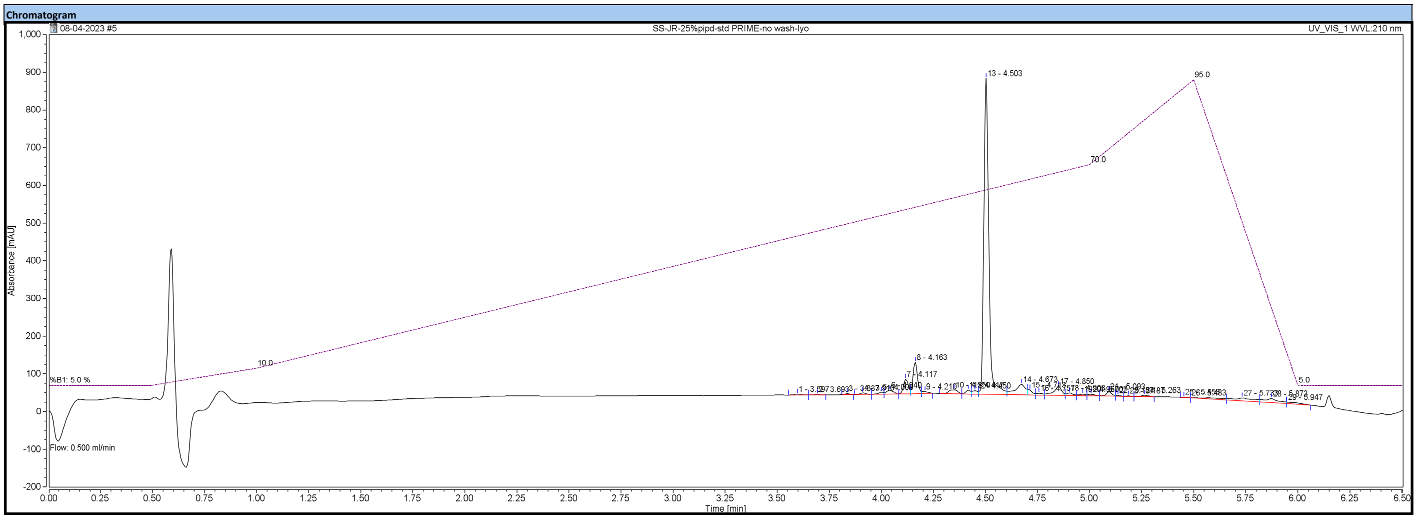

No.	Retention Time	Area	Height	Relative Area	Relative Height
	min	mAU*min	mAU	%	%
UV_VIS_1	UV_VIS_1	UV_VIS_1	UV_VIS_1	UV_VIS_1	UV_VIS_1
1	3.597	0.082	2.125	0.23	0.19
2	3.693	0.034	0.923	0.10	0.08
3	3.837	0.060	2.573	0.17	0.22
4	3.910	0.137	4.374	0.39	0.38
5	4.000	0.149	5.318	0.42	0.46
6	4.040	0.388	9.801	1.09	0.85
7	4.117	1.040	38.717	2.93	3.37
8	4.163	2.100	82.447	5.92	7.18
9	4.210	0.174	4.910	0.49	0.43
10	4.350	0.540	10.529	1.52	0.92
11	4.417	0.287	10.285	0.81	0.90
12	4.450	0.270	9.323	0.76	0.81
13	4.503	23.336	837.828	65.81	72.99
14	4.673	1.601	27.568	4.52	2.40
15	4.713	0.326	13.109	0.92	1.14
16	4.757	0.169	4.769	0.48	0.42
17	4.850	1.062	23.844	2.99	2.08
18	4.903	0.235	6.784	0.66	0.59
19	4.967	0.155	4.287	0.44	0.37
20	5.007	0.157	4.081	0.44	0.36
21	5.093	0.375	12.128	1.06	1.06
22	5.137	0.059	2.217	0.17	0.19
23	5.187	0.044	1.329	0.12	0.12
24	5.263	0.155	3.689	0.44	0.32
25	5.453	0.028	0.829	0.08	0.07
26	5.483	0.402	0.568	1.13	0.05
27	5.733	0.901	8.159	2.54	0.71
28	5.873	0.877	11.148	2.47	0.97
29	5.947	0.315	4.159	0.89	0.36
Total:		35.461	1147.821	100.00	100.00

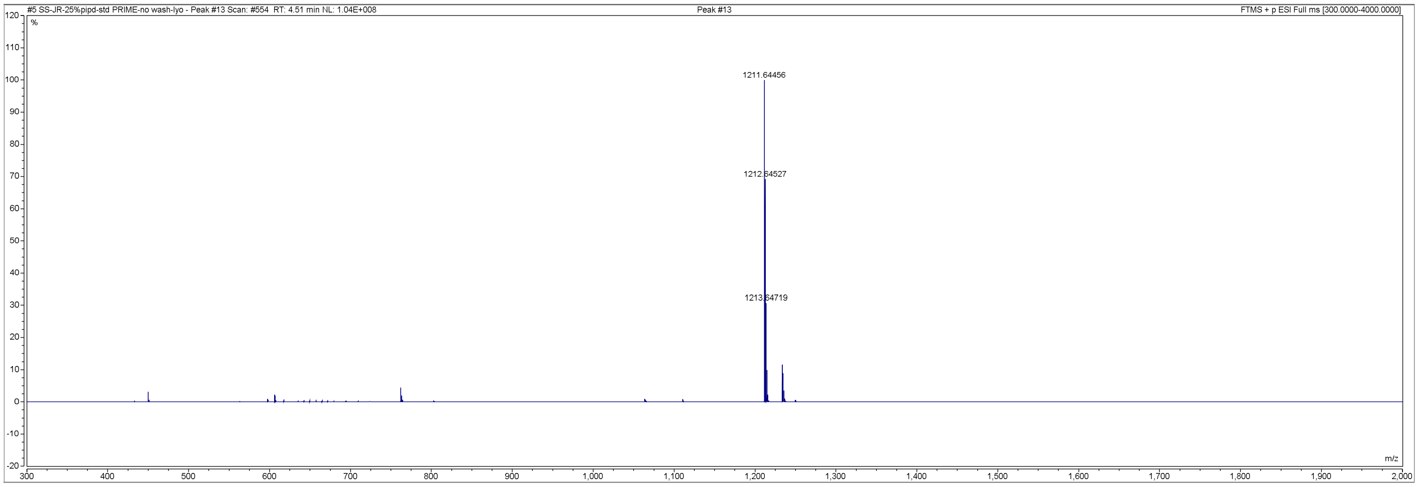

Chromeleon (c) Dionex
Version 7.2.10.23925

JR-Table 1, entry 22

Chromatogram and Results

Injection Details

Injection Name:	SS-JR-17%pipd-80 sec depro-No wash-PRIME-lyo	Run Time (min):	6.50
Vial Number:	G:F6	Injection Volume:	10.00
Injection Type:	Unknown	Channel:	UV_VIS_1
Calibration Level:		Wavelength:	210
Instrument Method:	ACN10-70_0.500uL_40C Waters Acquity BEH C8 1.7um. 2.1 x 100mm Column	Bandwidth:	4
Processing Method:	Quantitative	Dilution Factor:	1.0000
Injection Date/Time:	09/Aug/23 11:31	Sample Weight:	1.0000

Chromatogram

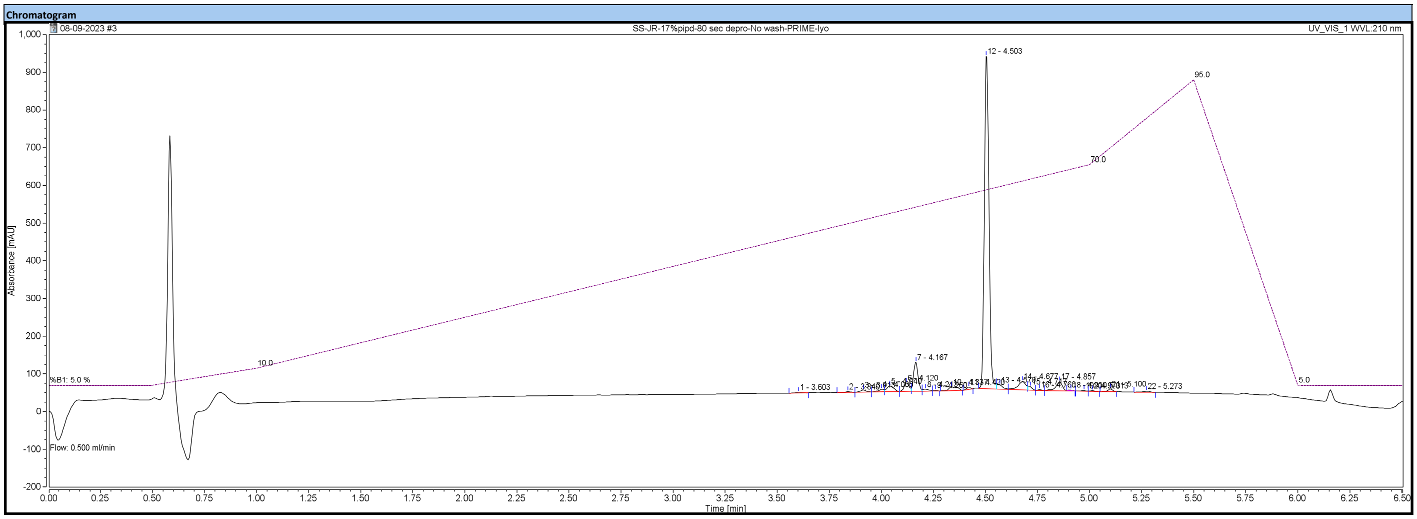

No.	Retention Time	Area	Height	Relative Area	Relative Height
	min	mAU*min	mAU	%	%
UV_VIS_1	UV_VIS_1	UV_VIS_1	UV_VIS_1	UV_VIS_1	UV_VIS_1
1	3.603	0.057	1.569	0.19	0.14
2	3.840	0.057	1.692	0.19	0.15
3	3.913	0.168	5.447	0.56	0.49
4	4.000	0.215	6.738	0.72	0.60
5	4.040	0.579	14.596	1.93	1.31
6	4.120	0.628	22.344	2.09	2.00
7	4.167	1.924	76.692	6.41	6.88
8	4.213	0.169	4.845	0.56	0.43
9	4.260	0.027	1.279	0.09	0.11
10	4.337	0.480	9.333	1.60	0.84
11	4.420	0.125	6.048	0.41	0.54
12	4.503	22.670	881.125	75.48	79.01
13	4.570	0.389	11.078	1.30	0.99
14	4.677	0.941	22.214	3.13	1.99
15	4.717	0.204	8.453	0.68	0.76
16	4.760	0.049	2.322	0.16	0.21
17	4.857	0.918	24.111	3.06	2.16
18	4.910	0.044	2.109	0.15	0.19
19	4.973	0.057	2.001	0.19	0.18
20	5.013	0.049	1.842	0.16	0.17
21	5.100	0.178	6.936	0.59	0.62
22	5.273	0.105	2.418	0.35	0.22
Total:		30.034	1115.192	100.00	100.00

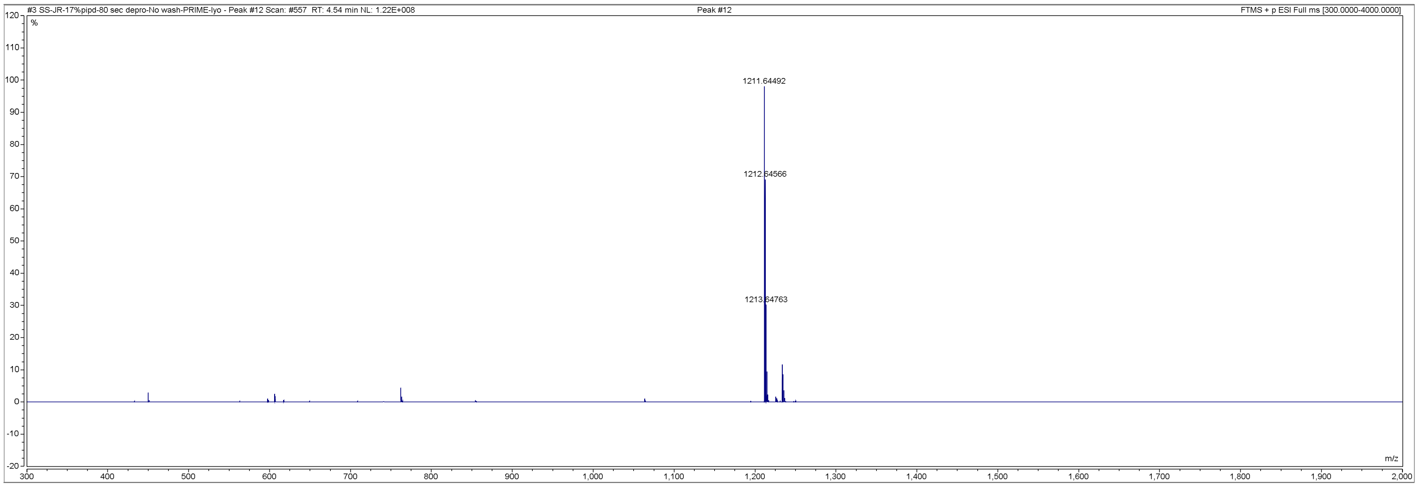

Chromeleon (c) Dionex
Version 7.2.10.23925

$^{65-74}$ACP-Supplementary Table 1, entry 1, wash-free

Chromatogram and Results

Injection Details

Injection Name:	*TW_ACP no wash (8/12/22), DMF, PRIME, 0.1mmol_lyoph_80s, 17% depro (for paper)*	*Run Time (min):*	*6.50*
Vial Number:	*B:A4*	*Injection Volume:*	*10.00*
Injection Type:	*Unknown*	*Channel:*	*UV_VIS_1*
Calibration Level:		*Wavelength:*	*210*
Instrument Method:	*ACN10-70_0.500uL_40C Waters Acquity BEH C8 1.7um. 2.1 x 100mm Column*	*Bandwidth:*	*4*
Processing Method:	*Quantitative*	*Dilution Factor:*	*1.0000*
Injection Date/Time:	*12/Jan/23 15:22*	*Sample Weight:*	*1.0000*

Chromatogram

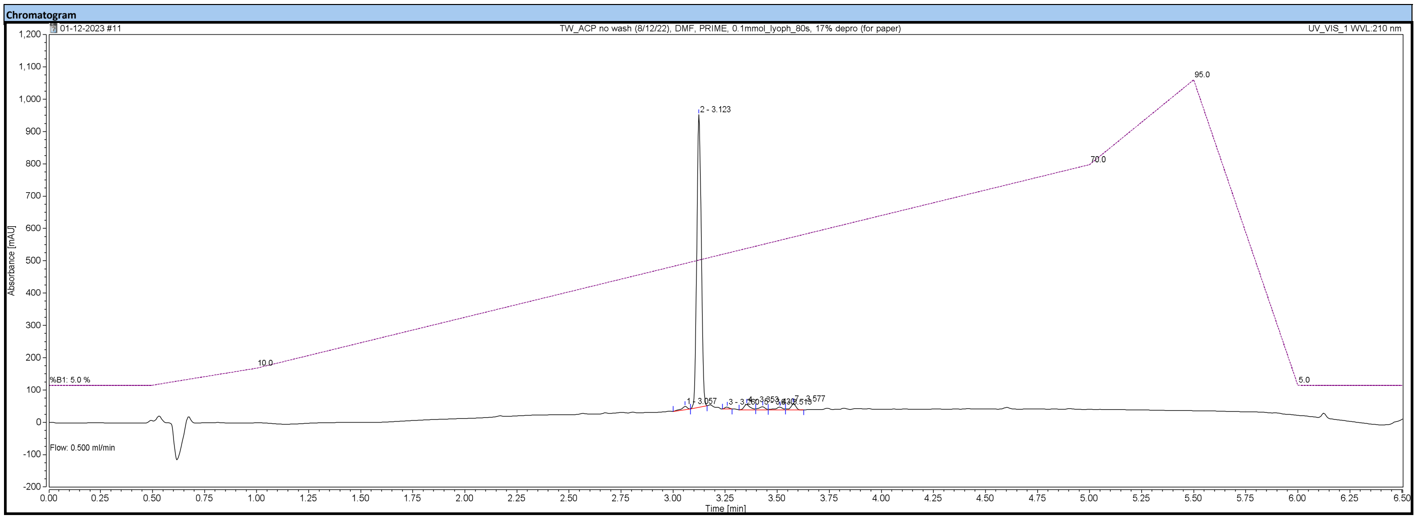

No.	Retention Time	Area	Height	Relative Area	Relative Height
	min	mAU*min	mAU	%	%
UV_VIS_1	UV_VIS_1	UV_VIS_1	UV_VIS_1	UV_VIS_1	UV_VIS_1
1	3.057	0.340	10.548	1.36	1.08
2	3.123	22.689	906.200	90.72	92.63
3	3.260	0.141	5.888	0.56	0.60
4	3.353	0.593	17.188	2.37	1.76
5	3.430	0.347	10.120	1.39	1.03
6	3.513	0.359	9.151	1.43	0.94
7	3.577	0.542	19.199	2.17	1.96
Total:		25.011	978.294	100.00	100.00

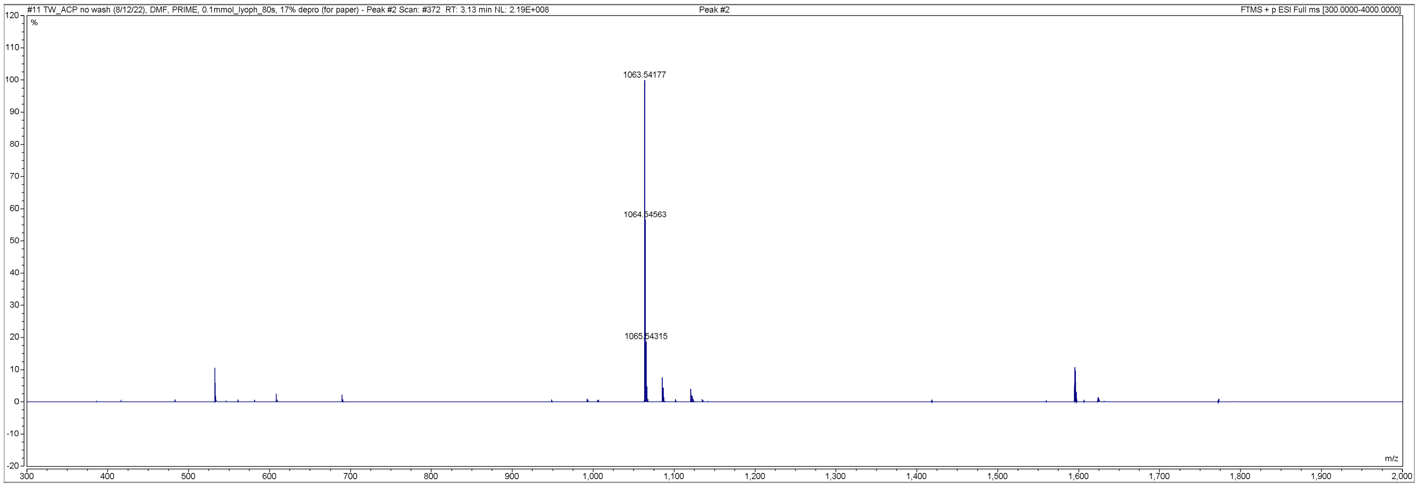

Chromeleon (c) Dionex
Version 7.2.10.23925

$^{65\text{-}74}$ACP-Supplementary Table 1, entry 1, wash-based

Chromatogram and Results

Injection Details

Injection Name:	TW_ACP WASH 0.1 mmol PRIME_lyoph_40s 17perc depro (1/25/23)	Run Time (min):	6.50
Vial Number:	B:D8	Injection Volume:	10.00
Injection Type:	Unknown	Channel:	UV_VIS_1
Calibration Level:		Wavelength:	210
Instrument Method:	ACN10-70_0.500uL_40C Waters Acquity BEH C8 1.7um. 2.1 x 100mm Column	Bandwidth:	4
Processing Method:	Quantitative	Dilution Factor:	1.0000
Injection Date/Time:	26/Jan/23 09:54	Sample Weight:	1.0000

Chromatogram

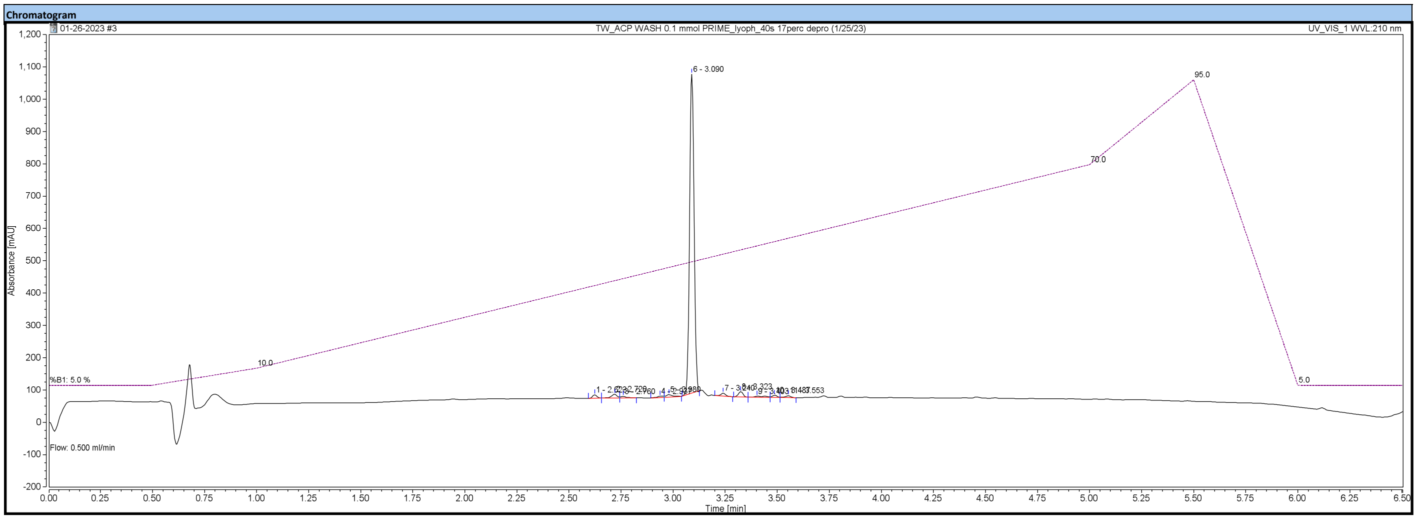

No.	Retention Time	Area	Height	Relative Area	Relative Height
	min	mAU*min	mAU	%	%
UV_VIS_1	UV_VIS_1	UV_VIS_1	UV_VIS_1	UV_VIS_1	UV_VIS_1
1	2.623	0.260	10.565	0.95	0.99
2	2.720	0.378	11.464	1.38	1.07
3	2.760	0.159	5.096	0.58	0.48
4	2.937	0.139	4.334	0.51	0.40
5	2.980	0.270	7.559	0.99	0.71
6	3.090	24.892	987.624	90.84	92.27
7	3.240	0.252	9.686	0.92	0.90
8	3.323	0.446	16.864	1.63	1.58
9	3.403	0.230	3.789	0.84	0.35
10	3.487	0.190	7.130	0.69	0.67
11	3.553	0.186	6.198	0.68	0.58
Total:		27.403	1070.309	100.00	100.00

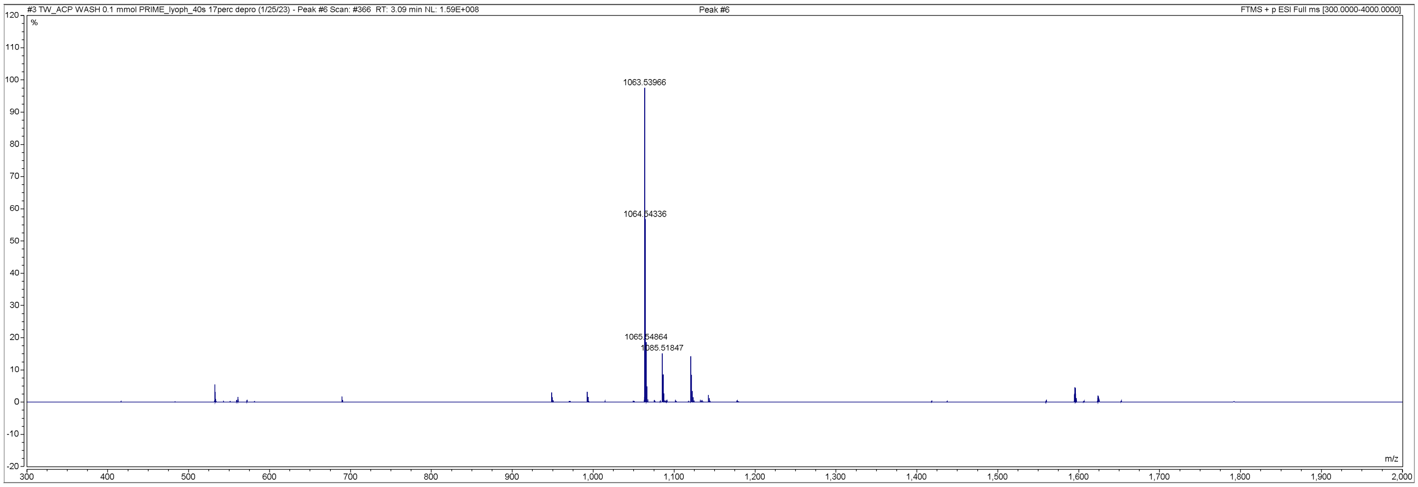

Chromeleon (c) Dionex
Version 7.2.10.23925

Liraglutide-Supplementary Table 1, entry 2, wash-free

Chromatogram and Results

Injection Details

Injection Name:	*TW_Liraglutide no wash paper, std cond_lyophilized_80s depro (plus NH4OH)*		*Run Time (min):*	*21.50*
Vial Number:	*Y:E6*		*Injection Volume:*	*10.00*
Injection Type:	*Unknown*		*Channel:*	*UV_VIS_1*
Calibration Level:			*Wavelength:*	*210*
Instrument Method:	*ACN10-90_0.500uL_20min_40C*	*Waters Acquity BEH C8 1.7um. 2.1 x 100mm Column*	*Bandwidth:*	*4*
Processing Method:	*Quantitative*		*Dilution Factor:*	*1.0000*
Injection Date/Time:	*03/Aug/22 12:39*		*Sample Weight:*	*1.0000*

Chromatogram

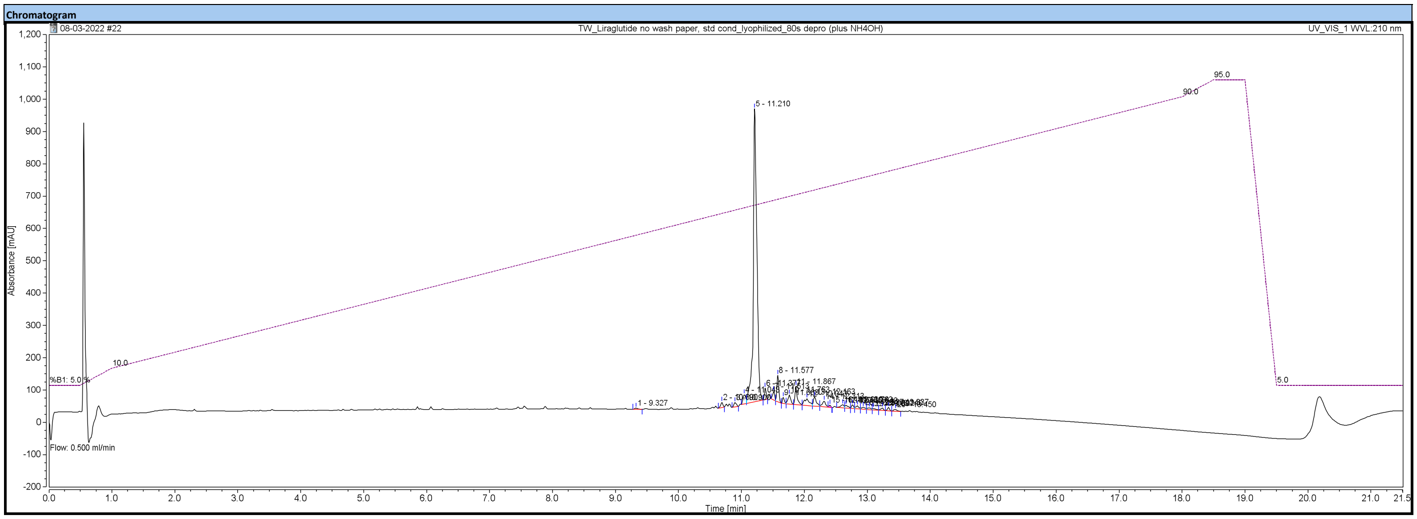

No.	Retention Time	Area	Height	Relative Area	Relative Height
	min	mAU*min	mAU	%	%
UV_VIS_1	UV_VIS_1	UV_VIS_1	UV_VIS_1	UV_VIS_1	UV_VIS_1
1	9.327	0.195	3.629	0.24	0.26
2	10.690	0.659	16.736	0.80	1.22
3	10.900	0.635	13.241	0.77	0.97
4	11.043	1.448	28.682	1.75	2.09
5	11.210	60.946	906.570	73.75	66.20
6	11.377	1.115	35.556	1.35	2.60
7	11.513	1.023	26.680	1.24	1.95
8	11.577	2.906	81.873	3.52	5.98
9	11.663	0.638	18.528	0.77	1.35
10	11.763	1.415	28.259	1.71	2.06
11	11.867	2.385	56.670	2.89	4.14
12	12.043	1.980	20.975	2.40	1.53
13	12.163	1.610	28.690	1.95	2.09
14	12.313	1.186	17.783	1.43	1.30
15	12.407	0.171	5.863	0.21	0.43
16	12.610	0.593	9.864	0.72	0.72
17	12.663	0.518	9.096	0.63	0.66
18	12.763	0.418	11.468	0.51	0.84
19	12.843	0.532	9.129	0.64	0.67
20	12.937	0.427	6.056	0.52	0.44
21	13.030	0.369	5.481	0.45	0.40
22	13.097	0.286	4.375	0.35	0.32
23	13.240	0.405	8.107	0.49	0.59
24	13.337	0.435	10.617	0.53	0.78
25	13.450	0.350	5.577	0.42	0.41
Total:		82.644	1369.505	100.00	100.00

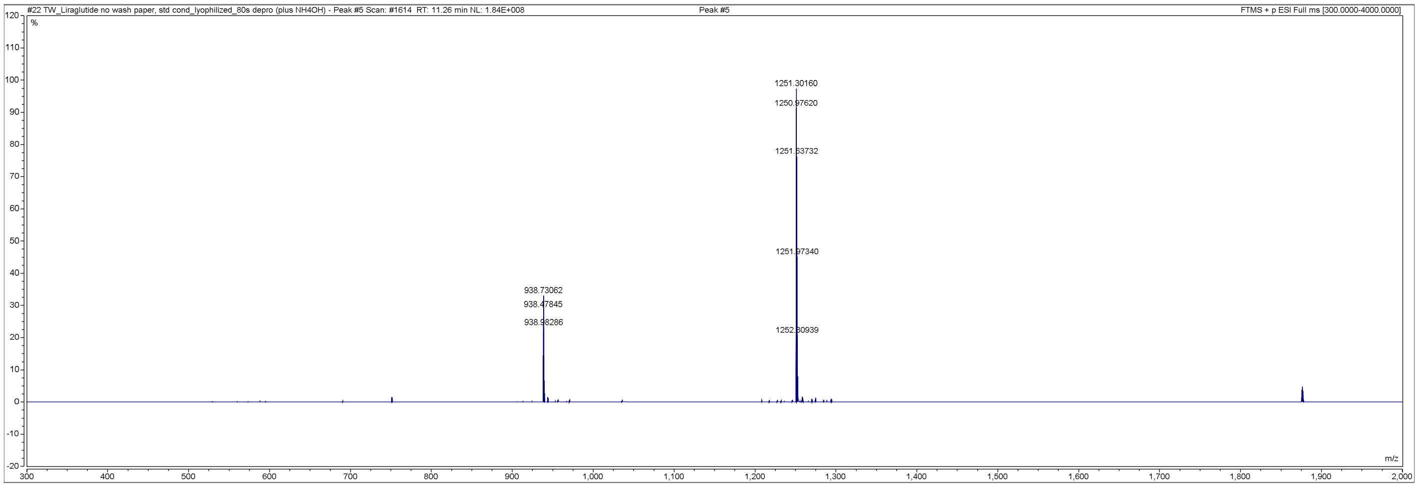

Chromeleon (c) Dionex
Version 7.2.10.23925

Liraglutide-Supplementary Table 1, entry 2, wash-based

Chromatogram and Results

Injection Details

Injection Name:	*TW_Liraglutide WASH, 0.1 mmol, PRIME, DMF_lyoph_80s 17% depro*	*Run Time (min):*	*21.50*
Vial Number:	*Y:A3*	*Injection Volume:*	*10.00*
Injection Type:	*Unknown*	*Channel:*	*UV_VIS_1*
Calibration Level:		*Wavelength:*	*210*
Instrument Method:	*ACN10-90_0.500uL_20min_40C* *Waters Acquity BEH C8 1.7um. 2.1 x 100mm Column*	*Bandwidth:*	*4*
Processing Method:	*Quantitative*	*Dilution Factor:*	*1.0000*
Injection Date/Time:	*30/Nov/22 11:12*	*Sample Weight:*	*1.0000*

Chromatogram

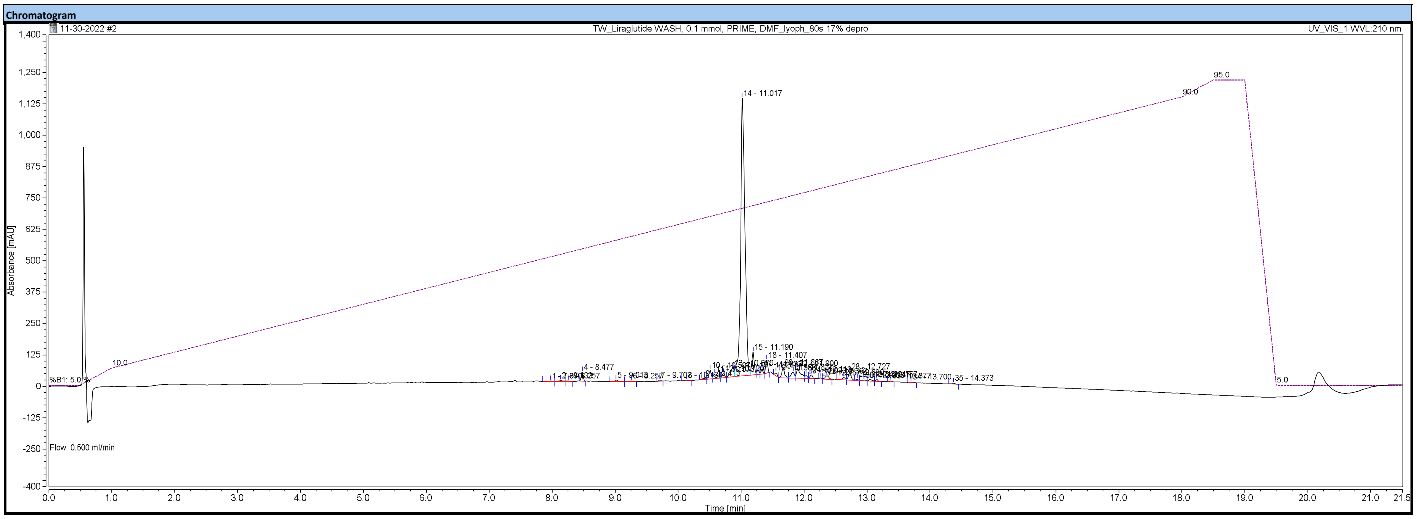

No.	Retention Time	Area	Height	Relative Area	Relative Height
	min	mAU*min	mAU	%	%
UV_VIS_1	UV_VIS_1	UV_VIS_1	UV_VIS_1	UV_VIS_1	UV_VIS_1
1	7.970	0.187	2.567	0.16	0.15
2	8.133	0.405	3.986	0.35	0.24
3	8.257	0.194	2.603	0.17	0.15
4	8.477	1.240	36.327	1.06	2.15
5	9.013	0.425	6.211	0.36	0.37
6	9.257	0.234	3.099	0.20	0.18
7	9.707	0.198	5.093	0.17	0.30
8	10.130	0.131	2.459	0.11	0.15
9	10.413	0.252	4.380	0.22	0.26
10	10.507	1.494	34.019	1.28	2.01
11	10.573	1.313	17.049	1.13	1.01
12	10.720	0.854	15.993	0.73	0.95
13	10.870	2.355	35.574	2.02	2.11
14	11.017	86.726	1105.349	74.44	65.43
15	11.190	4.172	90.856	3.58	5.38
16	11.267	0.796	18.438	0.68	1.09
17	11.333	0.709	21.240	0.61	1.26
18	11.407	1.859	51.235	1.60	3.03
19	11.553	0.496	12.617	0.43	0.75
20	11.667	2.042	41.306	1.75	2.45
21	11.827	1.453	23.826	1.25	1.41
22	11.900	2.885	38.404	2.48	2.27
23	12.033	0.529	11.203	0.45	0.66
24	12.113	0.673	15.425	0.58	0.91
25	12.257	0.155	3.960	0.13	0.23
26	12.363	0.970	14.155	0.83	0.84
27	12.630	0.529	9.549	0.45	0.57
28	12.727	1.748	33.468	1.50	1.98
29	12.843	0.114	3.160	0.10	0.19
30	12.953	0.212	2.844	0.18	0.17
31	13.047	0.403	7.000	0.35	0.41
32	13.157	0.316	7.082	0.27	0.42
33	13.377	0.153	3.680	0.13	0.22
34	13.700	0.144	2.395	0.12	0.14
35	14.373	0.141	2.725	0.12	0.16
Total:		116.506	1689.277	100.00	100.00

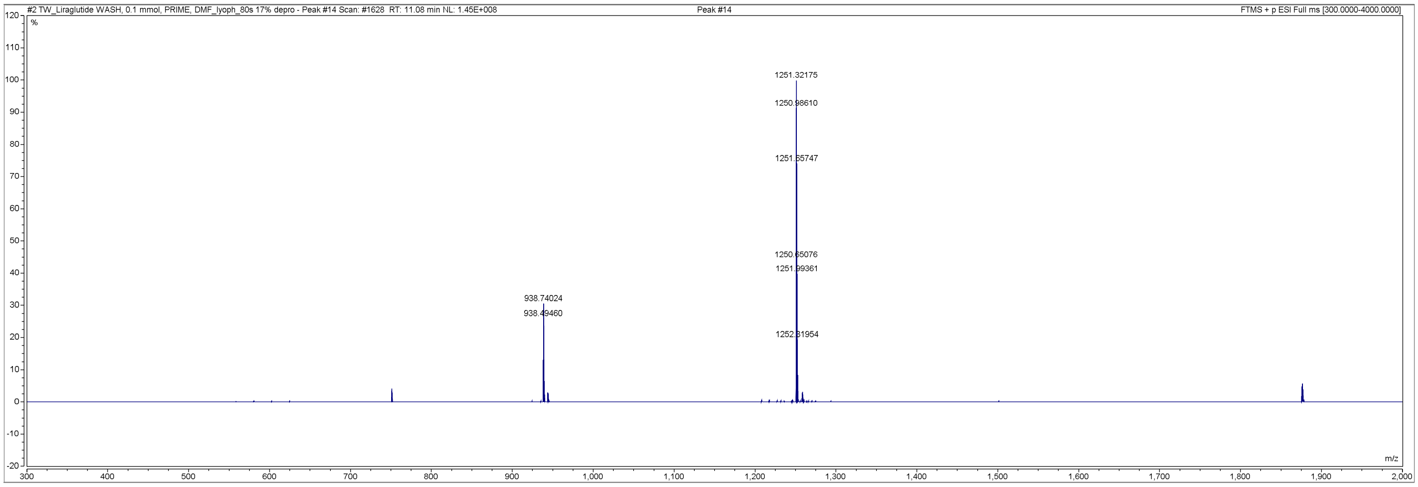

Chromeleon (c) Dionex
Version 7.2.10.23925

Semaglutide-Supplementary Table 1, entry 3, wash-free

Chromatogram and Results

Injection Details

Injection Name:	TW_Semaglutide-5 no wash 0.1mmol PRIME_lyoph_110C/80s 17% depro	Run Time (min):	21.50
Vial Number:	G:B7	Injection Volume:	10.00
Injection Type:	Unknown	Channel:	UV_VIS_1
Calibration Level:		Wavelength:	210
Instrument Method:	ACN10-90_0.500uL_20min_40C Waters Acquity BEH C8 1.7um. 2.1 x 100mm Column	Bandwidth:	4
Processing Method:	Quantitative	Dilution Factor:	1.0000
Injection Date/Time:	16/May/23 09:57	Sample Weight:	1.0000

Chromatogram

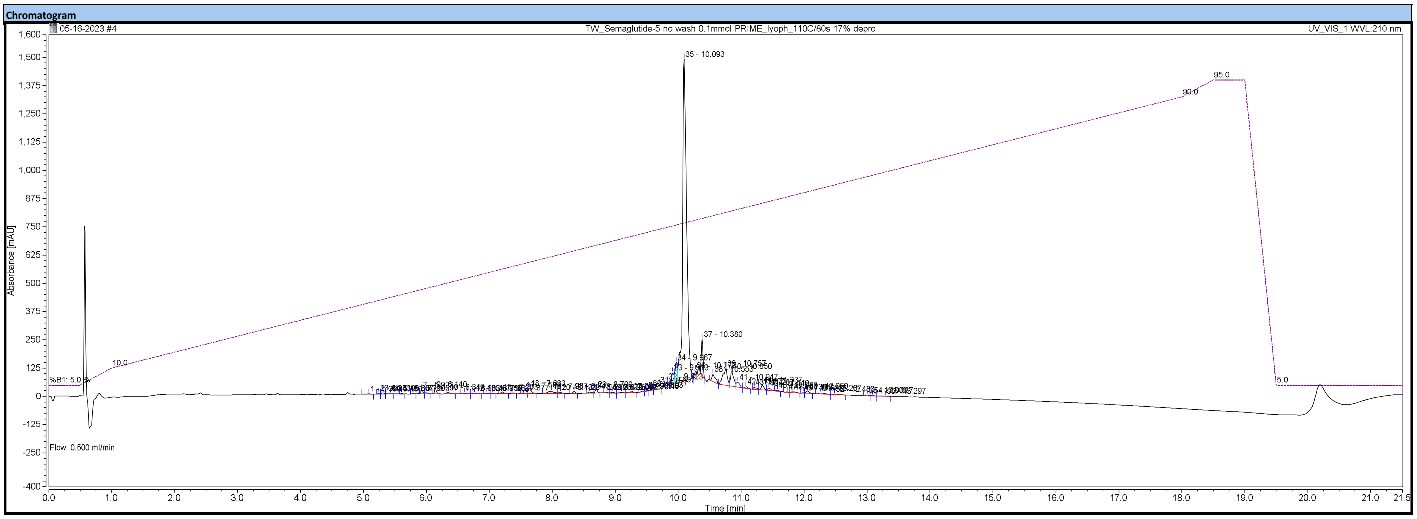

No.	Retention Time	Area	Height	Relative Area	Relative Height
	min	mAU*min	mAU	%	%
UV_VIS_1	UV_VIS_1	UV_VIS_1	UV_VIS_1	UV_VIS_1	UV_VIS_1
1	5.090	0.141	2.000	0.10	0.09
2	5.243	0.097	3.229	0.07	0.14
3	5.310	0.211	5.579	0.14	0.25
4	5.430	0.089	1.372	0.06	0.06
5	5.607	0.068	2.379	0.05	0.11
6	5.790	0.065	1.496	0.04	0.07
7	5.927	0.609	17.135	0.41	0.76
8	5.997	0.239	4.943	0.16	0.22
9	6.140	0.737	19.810	0.50	0.88
10	6.347	0.279	6.949	0.19	0.31
11	6.603	0.101	1.962	0.07	0.09
12	6.767	0.238	2.735	0.16	0.12
13	6.950	0.069	0.904	0.05	0.04
14	7.053	0.063	1.812	0.04	0.08
15	7.203	0.333	7.376	0.23	0.33
16	7.377	0.084	2.267	0.06	0.10
17	7.563	0.743	15.568	0.51	0.69
18	7.633	1.269	23.272	0.86	1.03
19	7.987	1.087	9.666	0.74	0.43
20	8.130	0.225	3.808	0.15	0.17
21	8.343	0.415	11.316	0.28	0.50
22	8.597	0.305	4.768	0.21	0.21
23	8.700	0.667	16.443	0.45	0.73
24	8.870	0.092	2.080	0.06	0.09
25	8.960	0.212	3.675	0.14	0.16
26	9.097	0.164	2.063	0.11	0.09
27	9.270	0.367	4.949	0.25	0.22
28	9.440	0.112	2.558	0.08	0.11
29	9.503	0.163	4.159	0.11	0.18
30	9.577	0.293	8.959	0.20	0.40
31	9.697	1.296	19.336	0.88	0.86
32	9.823	0.971	18.430	0.66	0.82
33	9.913	1.999	50.749	1.36	2.24
34	9.967	4.113	91.301	2.80	4.04
35	10.093	104.729	1423.867	71.25	62.98
36	10.277	0.733	22.867	0.50	1.01
37	10.380	5.157	158.542	3.51	7.01
38	10.553	2.079	33.104	1.41	1.46
39	10.757	5.859	73.924	3.99	3.27
40	10.850	3.949	65.791	2.69	2.91
41	10.947	1.363	22.253	0.93	0.98
42	11.077	0.392	6.619	0.27	0.29
43	11.207	0.525	9.464	0.36	0.42
44	11.337	1.141	23.204	0.78	1.03
45	11.440	0.699	11.068	0.48	0.49
46	11.577	0.158	3.576	0.11	0.16
47	11.730	0.082	1.389	0.06	0.06
48	11.823	0.359	3.989	0.24	0.18
49	11.983	0.084	2.323	0.06	0.10
50	12.060	0.562	10.166	0.38	0.45
51	12.267	0.573	4.935	0.39	0.22
52	12.483	0.312	2.643	0.21	0.12
53	13.000	0.144	3.272	0.10	0.14
54	13.087	0.055	1.282	0.04	0.06
55	13.297	0.124	1.337	0.08	0.06
Total:		146.994	2260.664	100.00	100.00

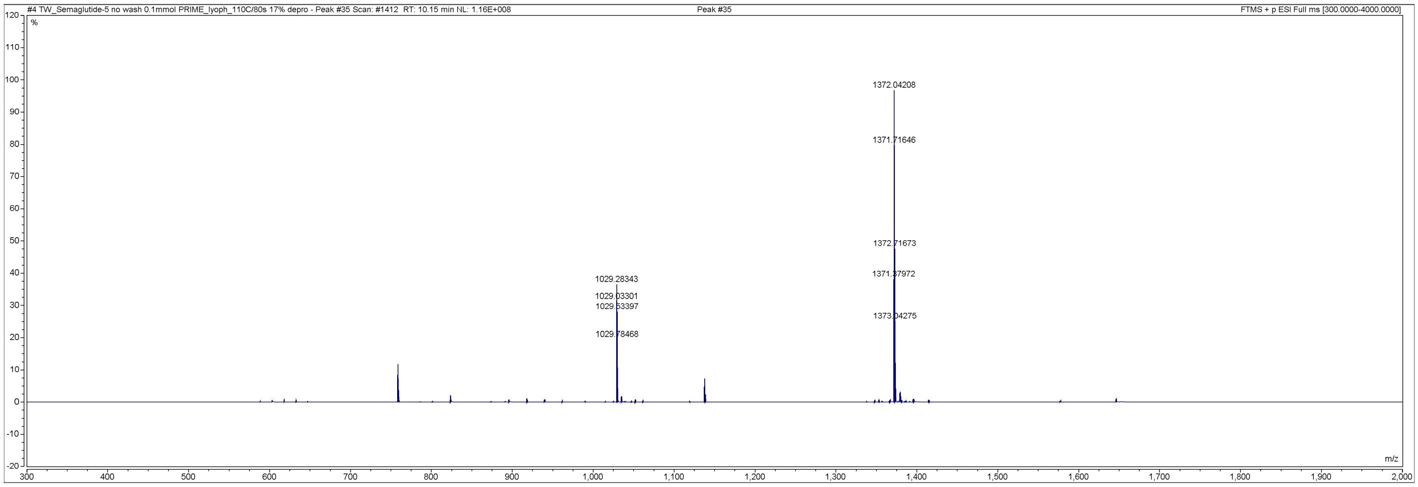

Chromeleon (c) Dionex
Version 7.2.10.23925

Semaglutide-Supplementary Table 1, entry 3, wash-based

Chromatogram and Results

Injection Details

Injection Name:	*TW_Semaglutide-2 WASH 0.1mmol PRIME_lyoph_110C/80s 17% depro*	*Run Time (min):*	*21.50*
Vial Number:	*G:B6*	*Injection Volume:*	*10.00*
Injection Type:	*Unknown*	*Channel:*	*UV_VIS_1*
Calibration Level:		*Wavelength:*	*210*
Instrument Method:	*ACN10-90_0.500uL_20min_40C* *Waters Acquity BEH C8 1.7um. 2.1 x 100mm Column*	*Bandwidth:*	*4*
Processing Method:	*Quantitative*	*Dilution Factor:*	*1.0000*
Injection Date/Time:	*16/May/23 09:34*	*Sample Weight:*	*1.0000*

Chromatogram

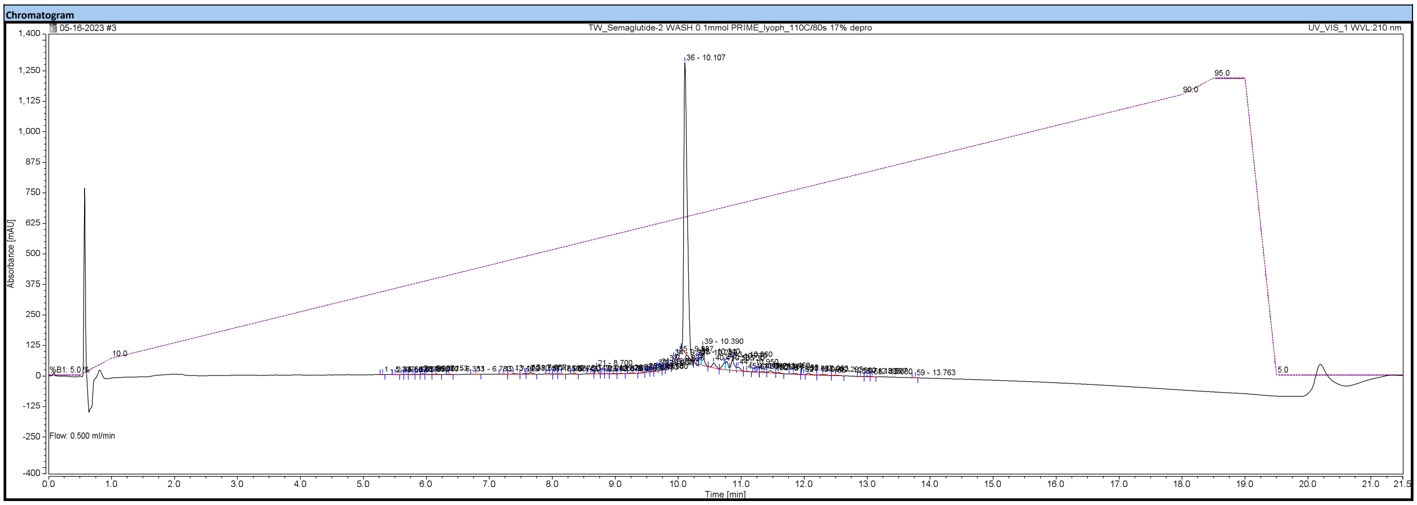

No.	Retention Time	Area	Height	Relative Area	Relative Height
	min	mAU*min	mAU	%	%
UV_VIS_1	UV_VIS_1	UV_VIS_1	UV_VIS_1	UV_VIS_1	UV_VIS_1
1	5.307	0.051	1.460	0.05	0.08
2	5.507	0.036	0.641	0.04	0.04
3	5.603	0.065	1.579	0.07	0.09
4	5.677	0.051	1.062	0.05	0.06
5	5.787	0.104	1.581	0.10	0.09
6	5.853	0.064	1.120	0.06	0.07
7	5.937	0.163	4.656	0.16	0.27
8	6.007	0.077	1.839	0.08	0.11
9	6.153	0.163	4.454	0.16	0.26
10	6.353	0.089	2.547	0.09	0.15
11	6.783	0.077	0.999	0.08	0.06
12	7.220	0.057	1.294	0.06	0.08
13	7.380	0.229	4.420	0.23	0.26
14	7.557	0.137	3.422	0.14	0.20
15	7.657	0.464	7.135	0.47	0.41
16	7.957	0.123	2.453	0.12	0.14
17	8.037	0.074	1.684	0.07	0.10
18	8.143	0.076	1.082	0.08	0.06
19	8.347	0.191	4.840	0.19	0.28
20	8.610	0.140	1.742	0.14	0.10
21	8.700	1.031	24.572	1.04	1.43
22	8.787	0.090	2.018	0.09	0.12
23	8.870	0.087	1.717	0.09	0.10
24	8.970	0.161	2.795	0.16	0.16
25	9.090	0.127	1.821	0.13	0.11
26	9.283	0.201	2.526	0.20	0.15
27	9.433	0.137	3.817	0.14	0.22
28	9.510	0.246	6.174	0.25	0.36
29	9.580	0.086	2.323	0.09	0.13
30	9.663	0.625	16.178	0.63	0.94
31	9.707	0.687	18.487	0.69	1.07
32	9.770	0.184	6.784	0.18	0.39
33	9.833	0.857	21.519	0.86	1.25
34	9.927	1.028	27.580	1.03	1.60
35	9.987	0.751	20.175	0.76	1.17
36	10.107	74.065	1187.759	74.57	68.98
37	10.287	1.159	25.026	1.17	1.45
38	10.340	1.147	31.966	1.15	1.86
39	10.390	3.117	77.856	3.14	4.52
40	10.563	1.352	20.694	1.36	1.20
41	10.730	1.401	27.169	1.41	1.58
42	10.770	1.684	36.224	1.70	2.10
43	10.860	2.578	45.238	2.60	2.63
44	10.950	0.991	15.722	1.00	0.91
45	11.087	0.139	2.592	0.14	0.15
46	11.213	0.116	3.076	0.12	0.18
47	11.260	0.020	0.822	0.02	0.05
48	11.360	0.306	4.409	0.31	0.26
49	11.460	0.716	10.011	0.72	0.58
50	11.590	0.217	3.236	0.22	0.19
51	11.827	0.241	2.908	0.24	0.17
52	11.990	0.020	0.721	0.02	0.04
53	12.063	0.460	6.432	0.46	0.37
54	12.293	0.412	3.646	0.41	0.21
55	12.500	0.199	2.000	0.20	0.12
56	12.893	0.057	1.174	0.06	0.07
57	12.997	0.106	2.319	0.11	0.13
58	13.080	0.046	1.128	0.05	0.07
59	13.763	0.048	1.215	0.05	0.07
Total:		99.323	1721.838	100.00	100.00

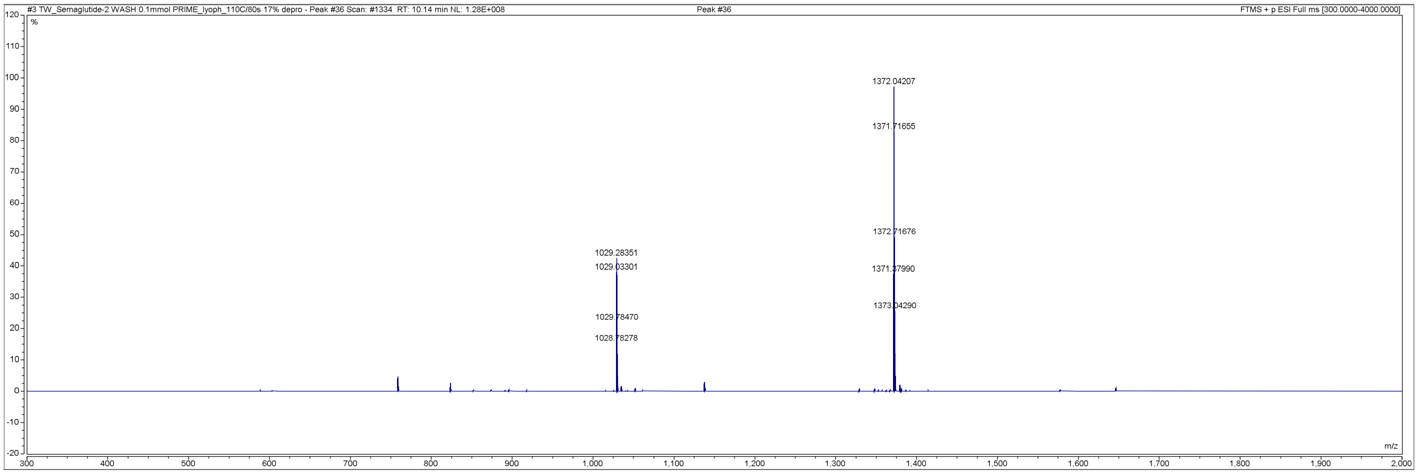

Chromeleon (c) Dionex
Version 7.2.10.23925

$^{1-42}$β- amyloid-Supplementary Table 1, entry 4, wash-free

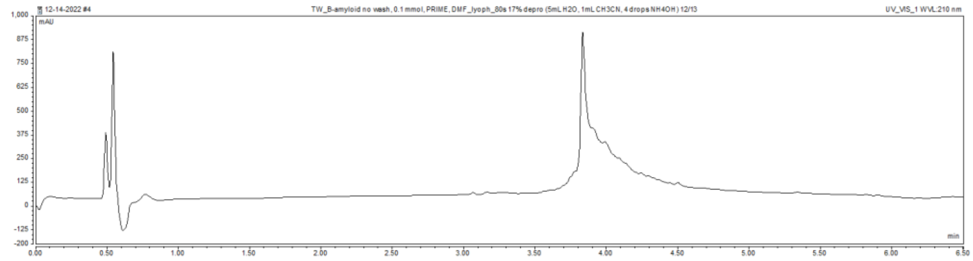

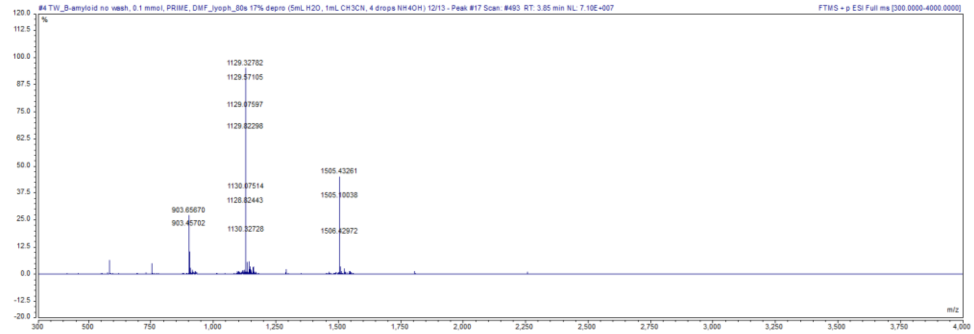

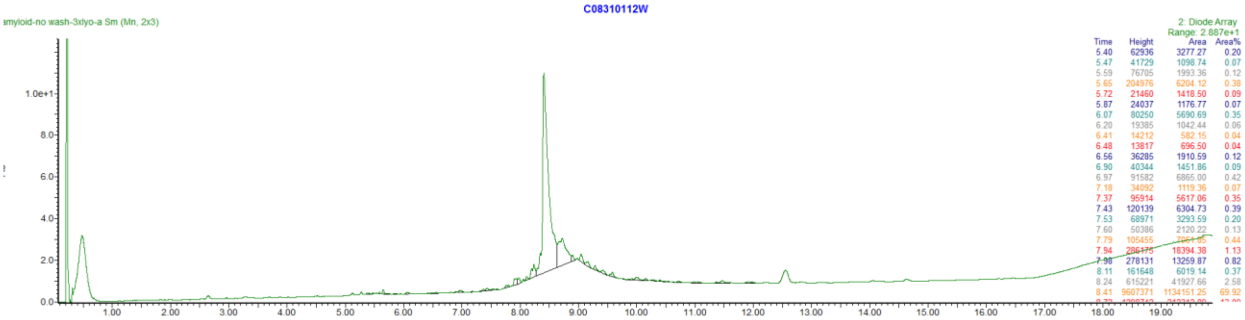

$^{1-42}$β- amyloid-Supplementary Table 1, entry 4, wash-based

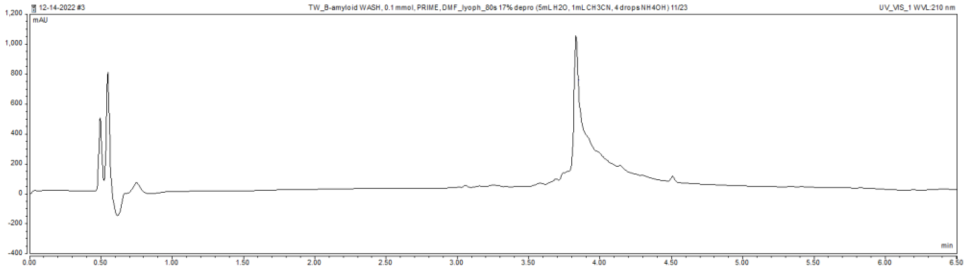

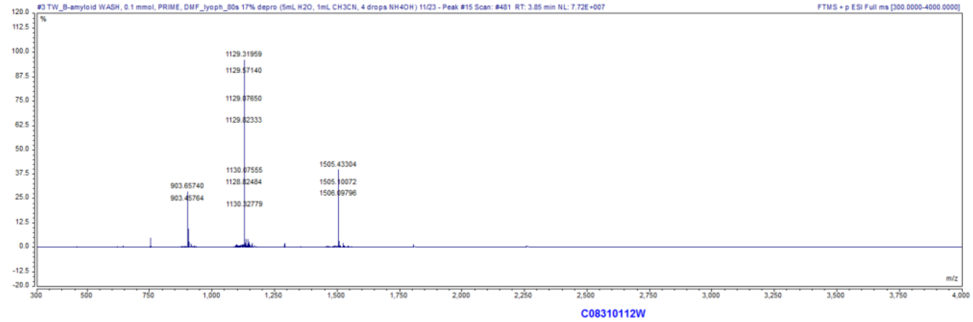

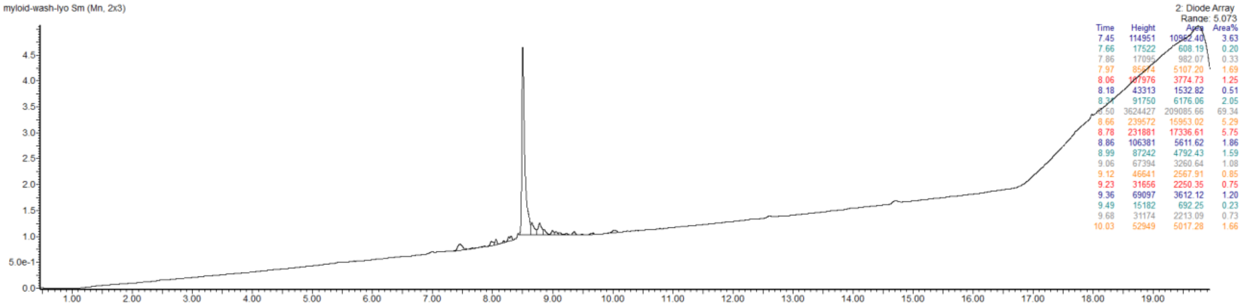

Liraglutide-Supplementary Table 1, entry 5, wash-free

Chromatogram and Results

Injection Details

Injection Name:	TW_Liraglutide PRO cond, no wash_lyophilized_8min/80C depro, 5min/80C couple, 17%depro	Run Time (min):	21.50
Vial Number:	Y:A6	Injection Volume:	10.00
Injection Type:	Unknown	Channel:	UV_VIS_1
Calibration Level:		Wavelength:	210
Instrument Method:	ACN10-90_0.500uL_20min_40C Waters Acquity BEH C8 1.7um. 2.1 x 100mm Column	Bandwidth:	4
Processing Method:	Quantitative	Dilution Factor:	1.0000
Injection Date/Time:	19/Aug/22 08:36	Sample Weight:	1.0000

Chromatogram

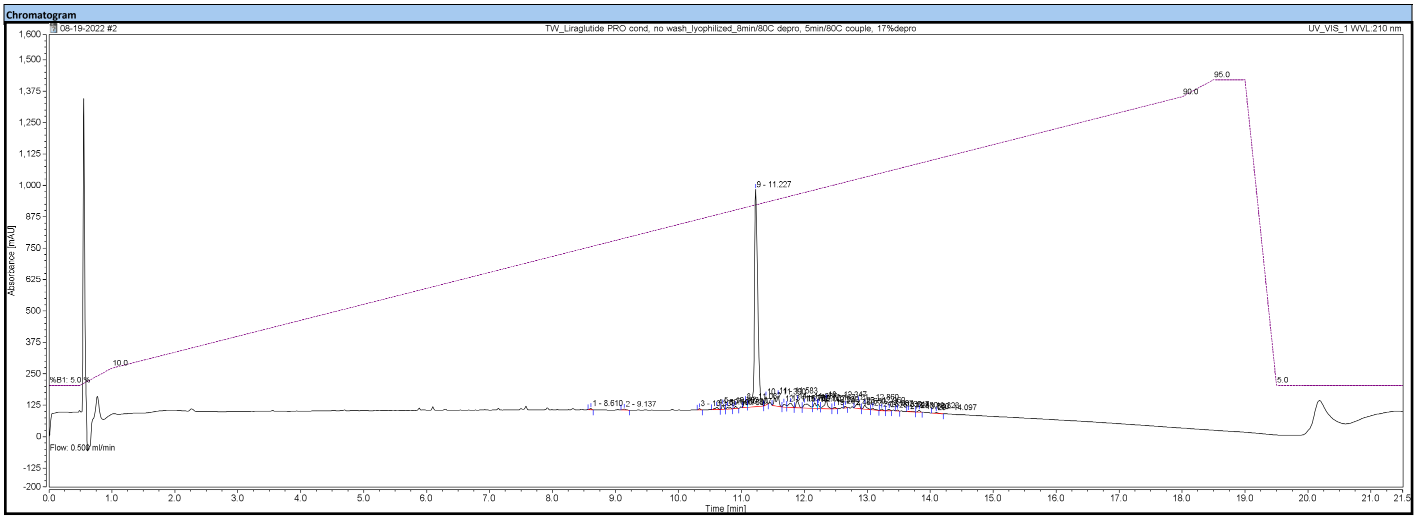

No.	Retention Time	Area	Height	Relative Area	Relative Height
	min	mAU*min	mAU	%	%
UV_VIS_1	UV_VIS_1	UV_VIS_1	UV_VIS_1	UV_VIS_1	UV_VIS_1
1	8.610	0.207	5.732	0.29	0.45
2	9.137	0.169	4.132	0.23	0.33
3	10.333	0.167	5.155	0.23	0.41
4	10.610	0.490	9.525	0.68	0.76
5	10.703	0.588	16.967	0.81	1.35
6	10.780	0.402	7.018	0.56	0.56
7	10.907	0.331	9.733	0.46	0.77
8	11.067	1.189	23.444	1.64	1.86
9	11.227	51.964	863.953	71.80	68.53
10	11.390	0.976	32.203	1.35	2.55
11	11.583	2.803	42.302	3.87	3.36
12	11.677	0.601	12.652	0.83	1.00
13	11.780	1.179	16.741	1.63	1.33
14	11.887	1.380	27.297	1.91	2.17
15	12.007	1.653	16.282	2.28	1.29
16	12.167	1.045	22.199	1.44	1.76
17	12.220	0.540	16.133	0.75	1.28
18	12.347	2.145	35.963	2.96	2.85
19	12.480	0.250	5.879	0.35	0.47
20	12.650	0.212	7.480	0.29	0.59
21	12.860	1.437	26.513	1.99	2.10
22	12.960	0.884	16.632	1.22	1.32
23	13.087	0.358	7.483	0.50	0.59
24	13.230	0.249	6.134	0.34	0.49
25	13.347	0.236	5.176	0.33	0.41
26	13.480	0.151	4.462	0.21	0.35
27	13.660	0.249	2.535	0.34	0.20
28	13.823	0.268	6.723	0.37	0.53
29	14.097	0.253	4.257	0.35	0.34
Total:		72.377	1260.707	100.00	100.00

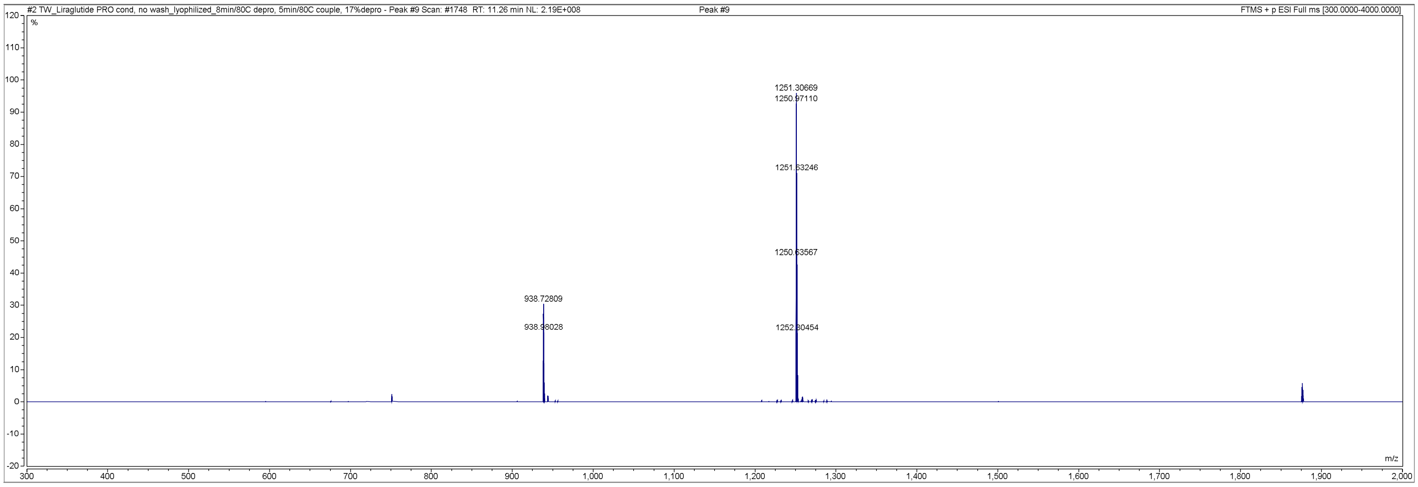

Chromeleon (c) Dionex
Version 7.2.10.23925

Liraglutide-Supplementary Table 1, entry 6, wash-free

Chromatogram and Results

Injection Details

Injection Name:	*Lira-25 mmol-PRO-No Wash-lyo-a*		*Run Time (min):*	*21.50*
Vial Number:	*G:B7*		*Injection Volume:*	*10.00*
Injection Type:	*Unknown*		*Channel:*	*UV_VIS_1*
Calibration Level:			*Wavelength:*	*210*
Instrument Method:	*ACN10-90_0.500uL_20min_40C*	*Waters Acquity BEH C8 1.7um. 2.1 x 100mm Column*	*Bandwidth:*	*4*
Processing Method:	*Quantitative*		*Dilution Factor:*	*1.0000*
Injection Date/Time:	*20/Feb/23 16:13*		*Sample Weight:*	*1.0000*

Chromatogram

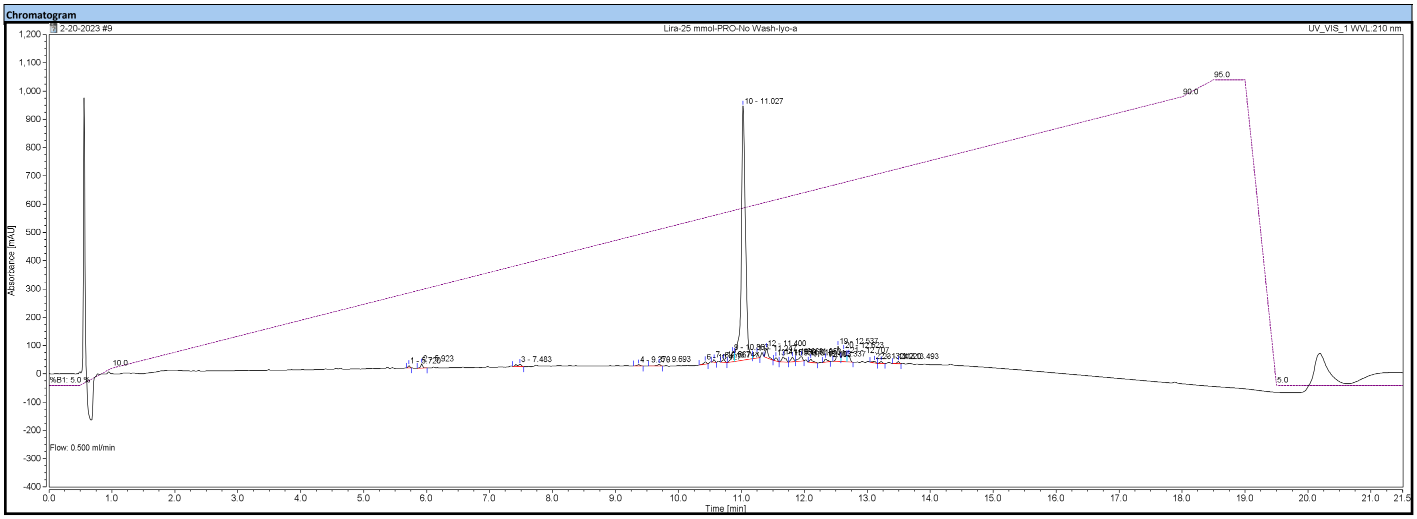

No.	Retention Time	Area	Height	Relative Area	Relative Height
	min	mAU*min	mAU	%	%
UV_VIS_1	UV_VIS_1	UV_VIS_1	UV_VIS_1	UV_VIS_1	UV_VIS_1
1	5.720	0.247	8.253	0.28	0.65
2	5.923	0.547	15.411	0.62	1.21
3	7.483	0.669	8.091	0.76	0.64
4	9.370	0.260	4.626	0.29	0.36
5	9.693	0.373	6.818	0.42	0.54
6	10.427	0.405	8.416	0.46	0.66
7	10.567	0.264	8.814	0.30	0.69
8	10.717	0.457	9.322	0.52	0.73
9	10.863	1.781	33.278	2.02	2.62
10	11.027	68.273	898.468	77.31	70.66
11	11.247	1.225	16.512	1.39	1.30
12	11.400	1.609	32.916	1.82	2.59
13	11.550	0.499	11.960	0.57	0.94
14	11.663	1.262	18.845	1.43	1.48
15	11.813	0.832	14.524	0.94	1.14
16	11.953	1.198	15.183	1.36	1.19
17	12.103	0.627	10.468	0.71	0.82
18	12.337	0.562	10.964	0.64	0.86
19	12.537	2.575	53.856	2.92	4.24
20	12.623	2.473	40.285	2.80	3.17
21	12.707	1.234	24.468	1.40	1.92
22	13.113	0.322	6.104	0.36	0.48
23	13.220	0.286	6.226	0.32	0.49
24	13.493	0.331	7.698	0.37	0.61
Total:		88.309	1271.506	100.00	100.00

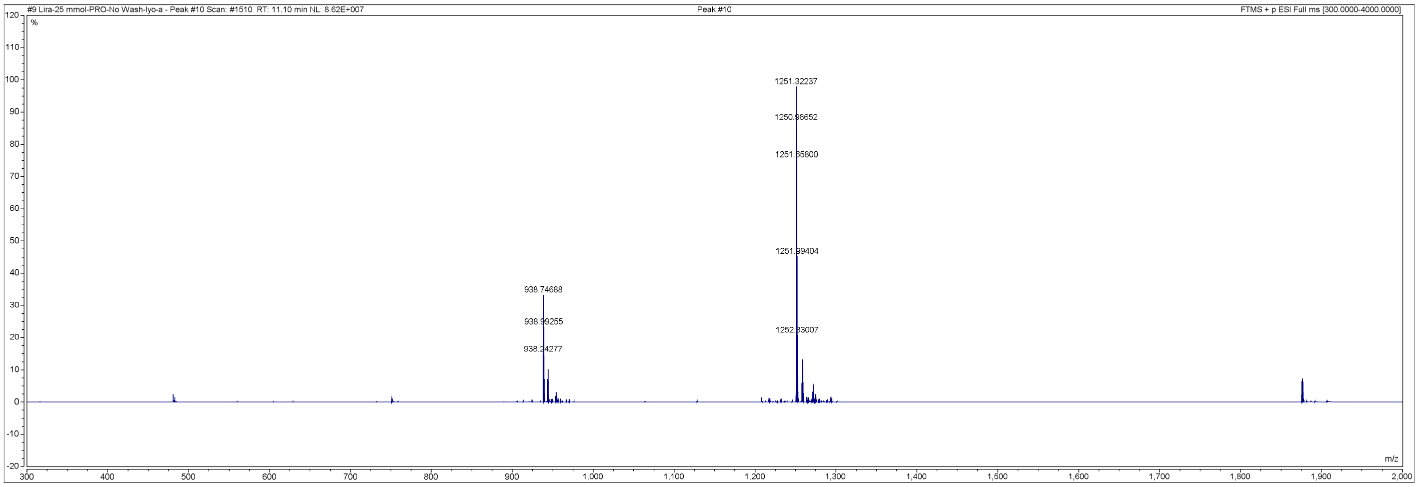

Chromeleon (c) Dionex
Version 7.2.10.23925

Liraglutide-Supplementary Table 1, entry 6, wash-based

Chromatogram and Results

Injection Details

Injection Name:	SS-Lira-25 mmol-wash-lyo-2		Run Time (min):	21.50
Vial Number:	G:F6		Injection Volume:	20.00
Injection Type:	Unknown		Channel:	UV_VIS_1
Calibration Level:			Wavelength:	210
Instrument Method:	ACN10-90_0.500uL_20min_40C	Waters Acquity BEH C8 1.7um. 2.1 x 100mm Column	Bandwidth:	4
Processing Method:	Quantitative		Dilution Factor:	1.0000
Injection Date/Time:	07/Sep/23 16:58		Sample Weight:	1.0000

Chromatogram

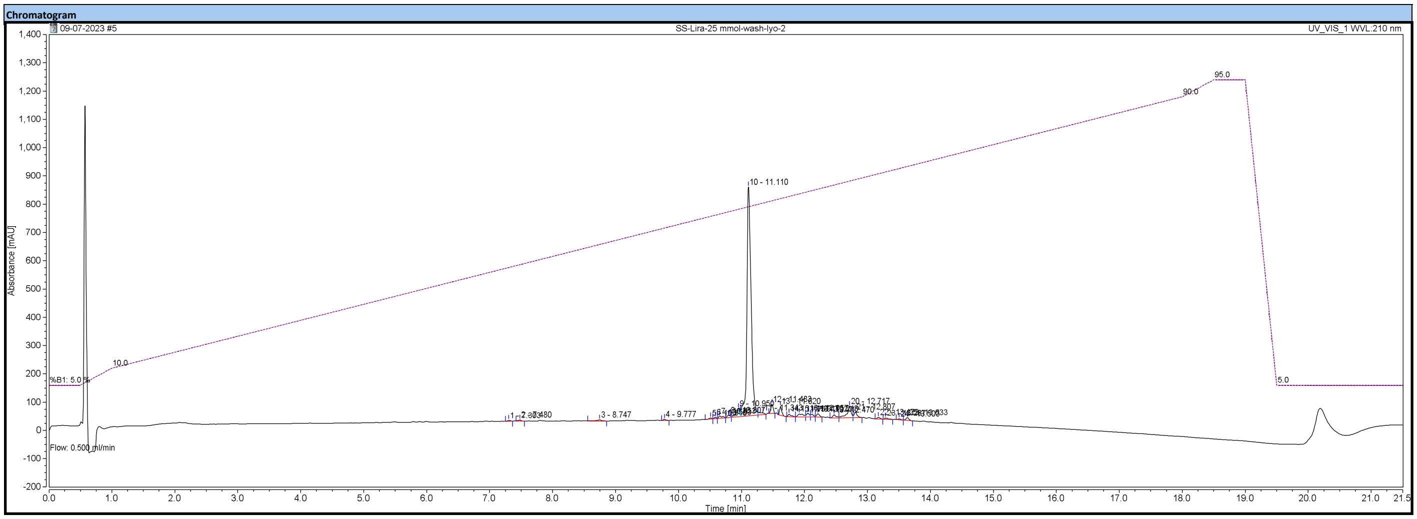

No.	Retention Time	Area	Height	Relative Area	Relative Height
	min	mAU*min	mAU	%	%
UV_VIS_1	UV_VIS_1	UV_VIS_1	UV_VIS_1	UV_VIS_1	UV_VIS_1
1	7.303	0.163	3.280	0.20	0.30
2	7.480	0.204	4.142	0.26	0.38
3	8.747	0.355	3.943	0.44	0.36
4	9.777	0.160	3.956	0.20	0.36
5	10.510	0.202	4.213	0.25	0.38
6	10.583	0.184	5.076	0.23	0.46
7	10.653	0.556	8.583	0.70	0.78
8	10.807	0.487	7.563	0.61	0.69
9	10.950	1.879	29.070	2.35	2.64
10	11.110	62.628	810.016	78.38	73.44
11	11.343	0.754	6.177	0.94	0.56
12	11.483	1.382	32.582	1.73	2.95
13	11.620	1.233	31.290	1.54	2.84
14	11.757	0.546	9.736	0.68	0.88
15	11.917	1.059	10.764	1.33	0.98
16	12.057	0.720	13.527	0.90	1.23
17	12.120	0.513	10.108	0.64	0.92
18	12.213	0.627	12.162	0.78	1.10
19	12.470	0.668	10.661	0.84	0.97
20	12.717	2.997	40.009	3.75	3.63
21	12.807	1.127	20.684	1.41	1.88
22	13.173	0.345	6.110	0.43	0.55
23	13.287	0.343	4.941	0.43	0.45
24	13.503	0.207	4.046	0.26	0.37
25	13.633	0.559	10.274	0.70	0.93
Total:		79.898	1102.915	100.00	100.00

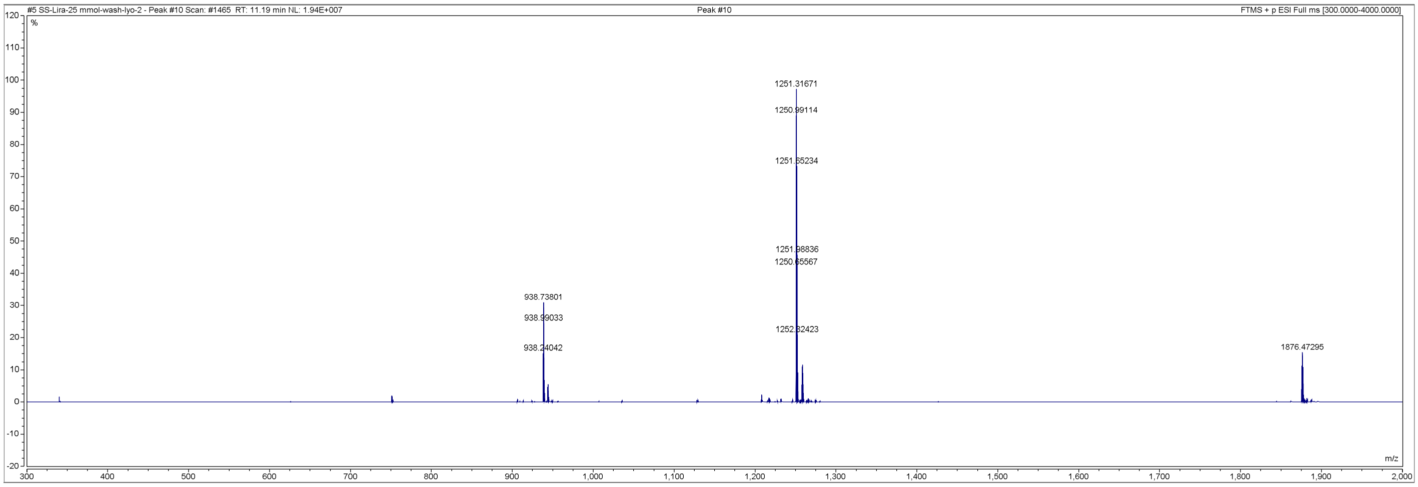

Chromeleon (c) Dionex
Version 7.2.10.23925

Certificate of Analysis

**Analysis number:**	**9998020-2/23**
**Analysis:**	Determination of optical purity of amino acid derivatives and peptides via GC-MS
**Sample name:**	Lira-25 mmol-no wash-purified
**Date of shipment:**	13.04.2023
**Sample receipt:**	17.04.2023
**Company:**	CEM
**Contact person:**	Dr. Sandeep Singh
**Date of completion:**	April 20, 2023

Completed:

(Operator)

Approved:

(Quality Assurance)

Out of Specification:
OOS is confirmed or defined by the customer. Investigation will be pursued in an stepwise approach: when all parameters of the analysis are checked and the analysis is verified, in a second approach further analyses are performed. If the results of the first analysis are not verified, root cause is defined, corrective action is done and if possible preventive action is taken.

Sitz: Tübingen-Hirschau; Registergericht: Stuttgart HRA 381110 Persönlich haftende Gesellschafterin: C.A.T. Beteiligungs-GmbH
Sitz: Tübingen-Hirschau; Registergericht: Stuttgart HRB 380849; Geschäftsführer: Dr. Jürgen Gerhardt, Dr. Heike Gerhardt

Analysis number 9998020-2/23	Lira-25 mmol-no wash-purified

Method description in accordance to SOP: A.0.3. /rv 200114

If Fmoc and DNP are present protective groups, they need to be cleaved.
The peptide / amino acid derivative is hydrolyzed in 6N DCl in D_2O. (In case of presence of Asn, it will be hydrolyzed to Asp, respectively Gln/Pyr to Glu, and so detected and determined).
If necessary an antioxidant and/or scavenger is added. After completion of hydrolysis excess of reagent is removed and the sample is esterified with deuterochloride in methyl alcohol. In accordance to the column specification homologue alcohols are possible. After evaporation of excess of reagent the residue is acylated using trifluoroacetic anhydrid or pentafluoropropionic anhydride. If histidine is to be determined, the -NH of the imidazol group is derivatized with propyl or butyl chloroformate in a separate step. The residue is dissolved and injected.

Calculation of enantiomeric purity:

$$\% D = \frac{Area_D}{Area_D + Area_L} * 100$$

Change Control:
Minor changes that are not in accordance to the method description are mentioned in the report. Major changes need to be approved.

System Suitability Test:

SST is running before each sequence. Results must meet the acceptance criteria.
For sensitive parameters performance qualification is determined weekly and must meet the acceptance criteria.

Data based on generic validation Revision 010213

Analyte:	:	Free proteinogenic amino acids
Standard deviation	:	≤± 0.1% (at ≤ 1.5% Enantiomer)<br>(For Cys and amino acids linked on to Cys possibly higher)
Limit of detection	:	<<0.1%
Limit of quantitation	:	0.10%
Range	:	0.10 - 5 % Enantiomer

The standard deviation of m-1 contribution that must be considered for some amino acids, is substance and matrix specific. Thus, also the standard deviation of the result for those amino acids could be higher. For amino acids which show complex mass pattern, such as Trp and Nal, standard deviation could be even higher than 0.3%.
If the LOQ of generic validation cannot be met due to substance-specific influences, the result will be reported as < <estimated LOQ >.

We assure that the analysis is performed in accordance to the GMP guidelines and in accordance to ICH guideline Q2(R1)

230103 A_0_3_e

Analysis number	Lira-25 mmol-no wash-purified
9998020-2/23	

Results: A.0.3. /rv 200114

The listed amino acid(s) were identified via retention time and mass spectra.
The identity of the main component(s) comply.
The following impurity of the optical antipode was found:

**Alanine**	0.10	% D-Enantiomer
**Valine**	<0.10	% D-Enantiomer
**Threonine**	<0.10	% D-Threonine
	<0.10	% D-allo Threonine
	<0.10	% L-allo Threonine
**Isoleucine**	<0.10	% D-Isoleucine
	<0.10	% D-allo-Isoleucine
	0.14	% L-allo-Isoleucine
**Leucine**	0.13	% D-Enantiomer
**Serine**	<0.10	% D-Enantiomer
**Aspartic acid**	0.18	% D-Enantiomer
**Phenylalanine**	0.14	% D-Enantiomer
**Glutamic acid**	0.25	% D-Enantiomer
**Tyrosine**	<0.10	% D-Enantiomer
**Lysine**	0.13	% D-Enantiomer
**Arginine**	0.10	% D-Enantiomer
**Tryptophan**	n.d.	*)
**Histidine**	0.47	% D-Enantiomer

Notes:

*) Tryptophan could not be determined.
Most likely, it was completely decomposed during sample preparation procedure. Only small differeces in the sample matrix can lead to decompostion.

The method is generically validated. However it may not meet all requirements for the release of drug substances and drug products. It is to prove if substance specific validation is required.

Sitz: Tübingen-Hirschau; Registergericht: Stuttgart HRA 381110 Persönlich haftende Gesellschafterin: C.A.T. Beteiligungs-GmbH
Sitz: Tübingen-Hirschau; Registergericht: Stuttgart HRB 380849; Geschäftsführer: Dr. Jürgen Gerhardt, Dr. Heike Gerhardt

Chromatogram Report

**Data file**	/HP9/Results/2023_04/XY020_2.rslt\XY020-02-01_1A1.dx
**Sample name**	Lira-25 mmol-no wash-purified

**Instrument**	HP#9	**Location**	94
**Injection date**	04/19/2023 13:57:49	**Injection volume [µl]**	0.200
**Acq. method**	S1419_ET_AAA_SIM_B.amx	**Acq. operator**	Verena Pohl (verena)
**Analysis method**	ET_AAA.pmx		

Signal: MS1Front SIM(140) EI

RT [min]	Type	Width [min]	Area	Height	Area%	Name
8.374	MM m	0.22	383.5	78.4	0.10	D Ala
9.698	BM m	0.77	383402.5	96088.1	99.90	L Ala

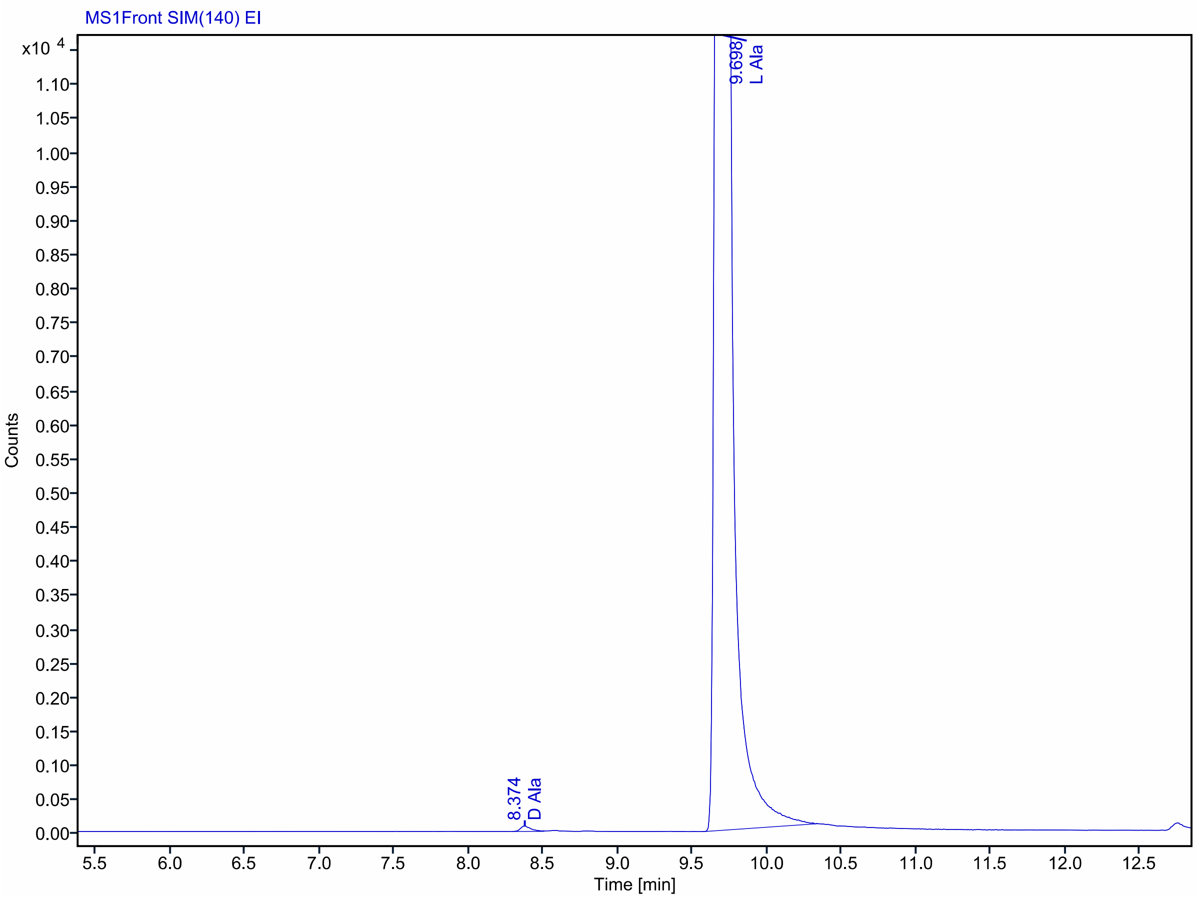

E-Signature

Level	Level Name	Signed By	Date	Comment	Meaning
1	Approver	Verena Pohl (verena)	2023-04-20 11:02:57+02:00		Approved

Chromatogram Report

**Data file**	/HP9/Results/2023_04/XY020_2.rslt\XY020-02-01_1A1.dx		
**Sample name**	Lira-25 mmol-no wash-purified		
**Instrument**	HP#9	**Location**	94
**Injection date**	04/19/2023 13:57:49	**Injection volume [µl]**	0.200
**Acq. method**	S1419_ET_AAA_SIM_B.amx	**Acq. operator**	Verena Pohl (verena)
**Analysis method**	ET_AAA.pmx		

Signal: MS1Front SIM(168) EI

RT [min]	Type	Width [min]	Area	Height	Area%	Name
11.994	MM m	0.18	45.4	9.6	0.04	D Val
12.950	BBA	0.98	117125.7	26393.9	99.96	L Val

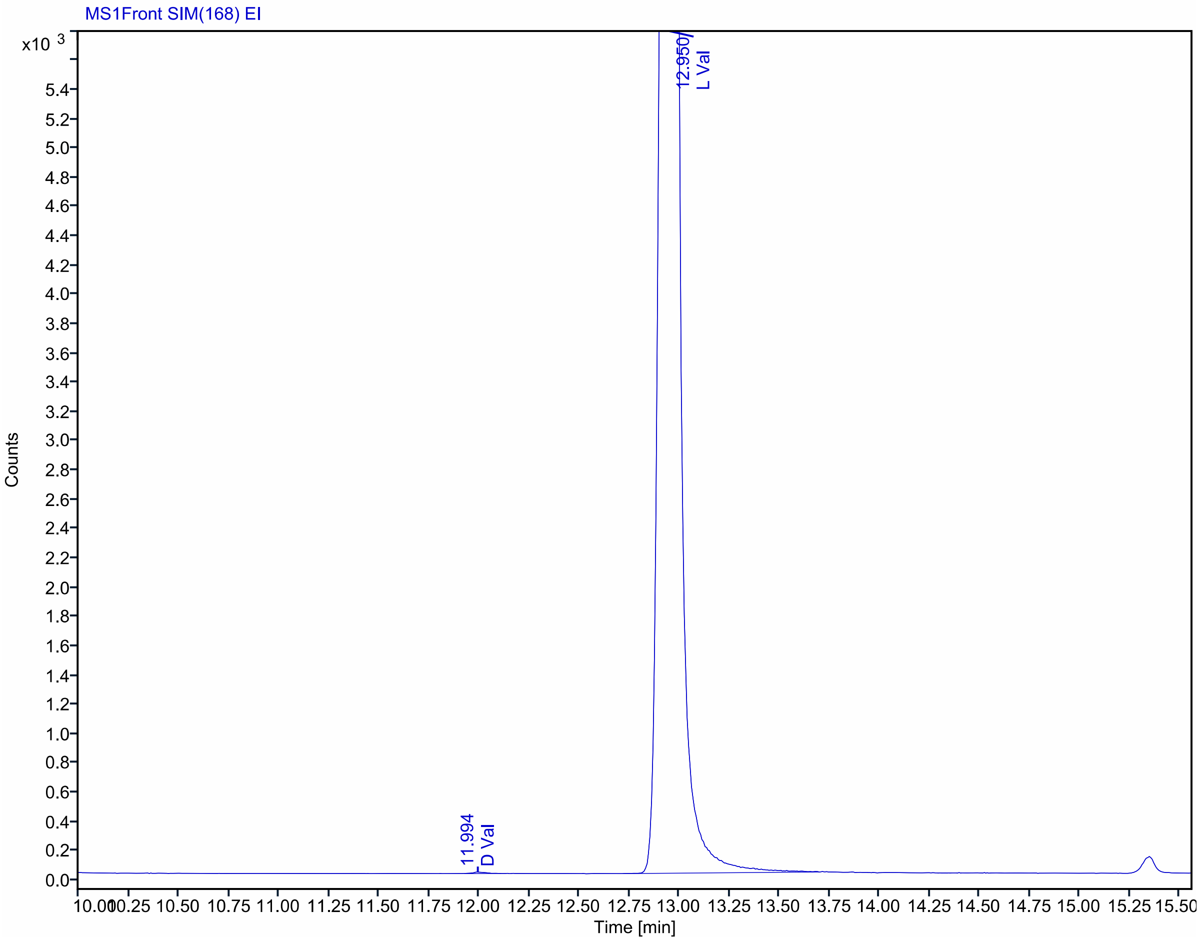

E-Signature

Level	Level Name	Signed By	Date	Comment	Meaning
1	Approver	Verena Pohl (verena)	2023-04-20 11:02:57+02:00		Approved

Chromatogram Report

**Data file**	/HP9/Results/2023_04/XY020_2.rslt\XY020-02-01_1A1.dx		
**Sample name**	Lira-25 mmol-no wash-purified		
**Instrument**	HP#9	**Location**	94
**Injection date**	04/19/2023 13:57:49	**Injection volume [µl]**	0.200
**Acq. method**	S1419_ET_AAA_SIM_B.amx	**Acq. operator**	Verena Pohl (verena)
**Analysis method**	ET_AAA.pmx		

Signal: MS1Front SIM(152) EI

RT [min]	Type	Width [min]	Area	Height	Area%	Name
14.375	MM m	0.1	8.7	2.4	0.01	D Thr
15.343	BBA	0.9	102719.5	24501.5	99.94	L Thr
19.559	MM m	0.1	19.2	5.6	0.02	D Ath
20.659	MM m	0.1	30.3	7.0	0.03	L Ath

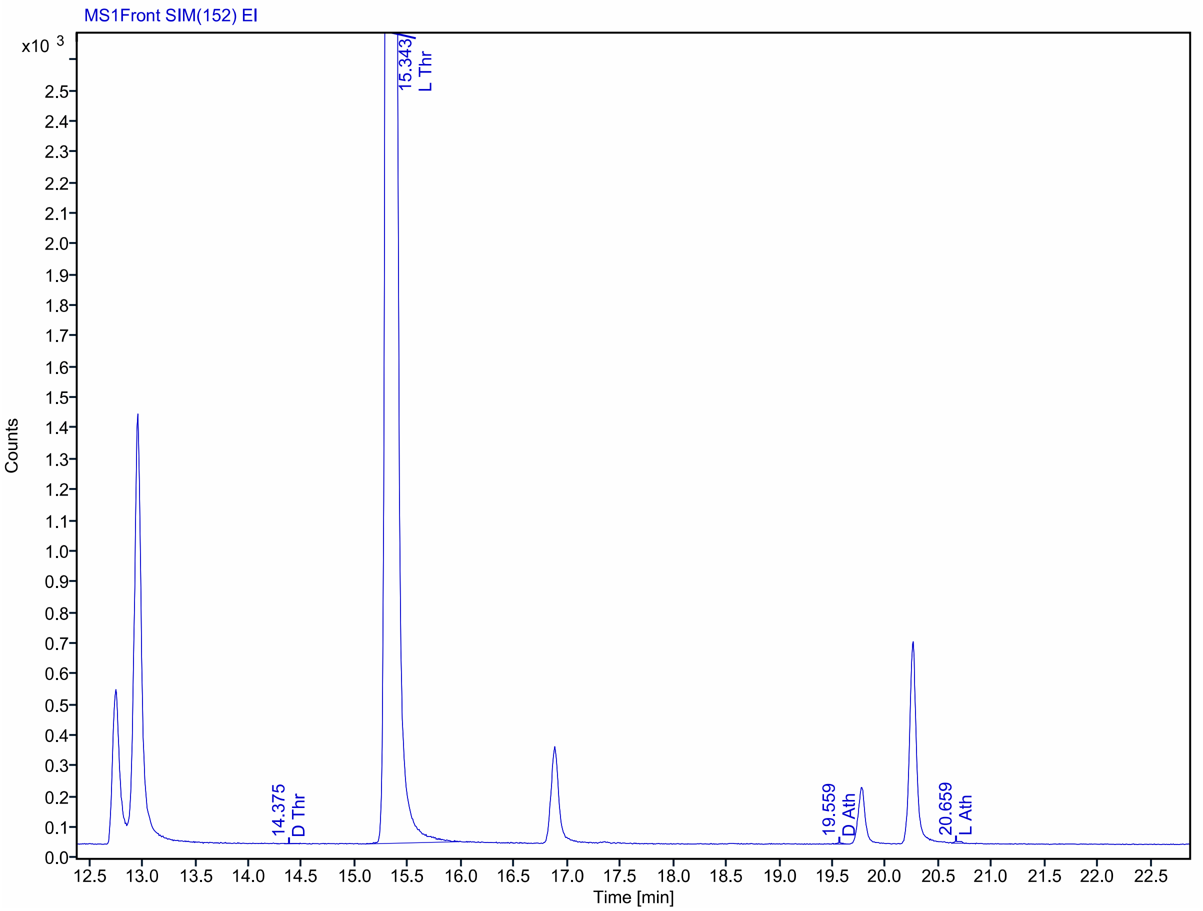

E-Signature

Level	Level Name	Signed By	Date	Comment	Meaning
1	Approver	Verena Pohl (verena)	2023-04-20 11:02:57+02:00		Approved

Chromatogram Report

**Data file**	/HP9/Results/2023_04/XY020_2.rslt\XY020-02-01_1A1.dx
**Sample name**	Lira-25 mmol-no wash-purified

**Instrument**	HP#9	**Location**	94
**Injection date**	04/19/2023 13:57:49	**Injection volume [µl]**	0.200
**Acq. method**	S1419_ET_AAA_SIM_B.amx	**Acq. operator**	Verena Pohl (verena)
**Analysis method**	ET_AAA.pmx		

Signal: MS1Front SIM(182) EI

RT [min]	Type	Width [min]	Area	Height	Area%	Name
15.137	MM m	0.13	17.3	3.9	0.08	D Ail
15.924	MM m	0.11	3.4	0.8	0.02	D Ile
16.305	MM m	0.16	29.0	7.0	0.14	L Ail
16.878	MM m	0.65	21029.7	4326.8	99.76	L Ile

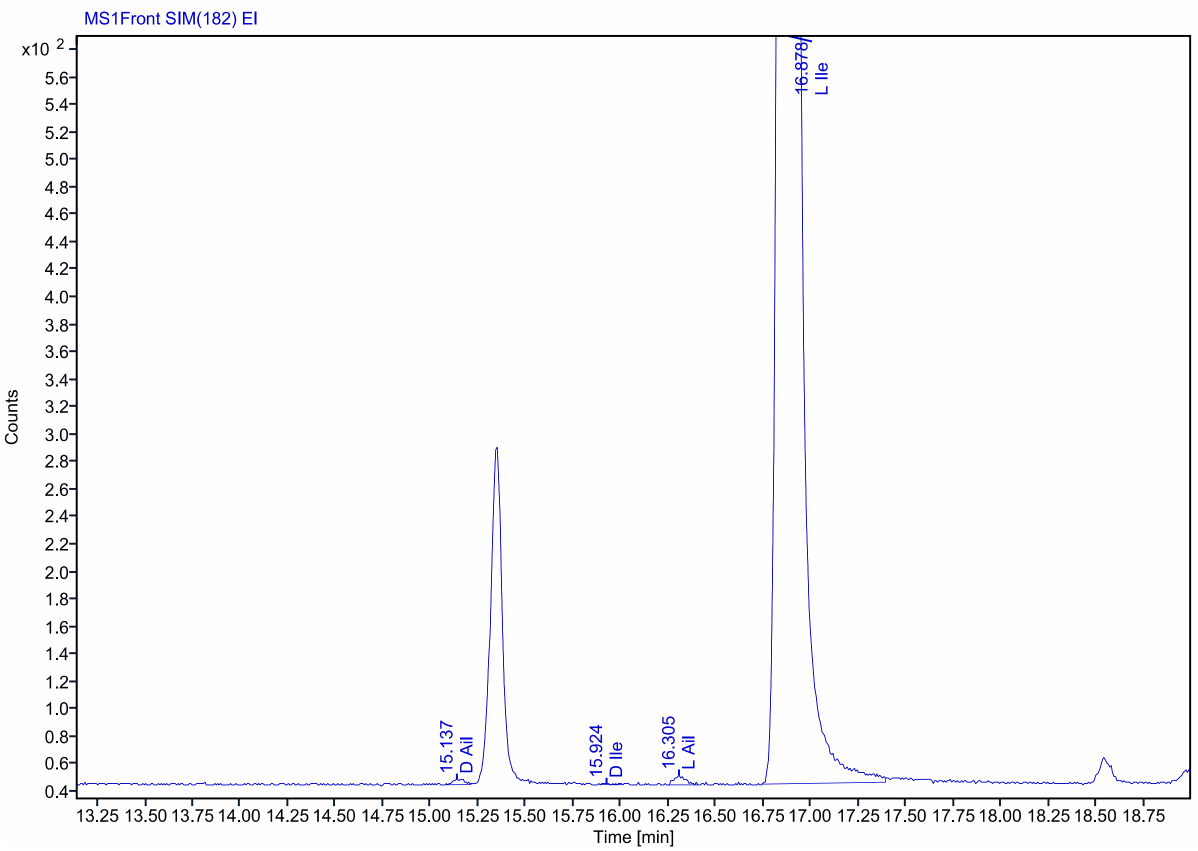

E-Signature

Level	Level Name	Signed By	Date	Comment	Meaning
1	Approver	Verena Pohl (verena)	2023-04-20 11:02:57+02:00		Approved

Chromatogram Report

**Data file**	/HP9/Results/2023_04/XY020_2.rslt\XY020-02-01_1A1.dx
**Sample name**	Lira-25 mmol-no wash-purified

**Instrument**	HP#9	**Location**	94
**Injection date**	04/19/2023 13:57:49	**Injection volume [µl]**	0.200
**Acq. method**	S1419_ET_AAA_SIM_B.amx	**Acq. operator**	Verena Pohl (verena)
**Analysis method**	ET_AAA.pmx		

Signal: MS1Front SIM(182) EI

RT [min]	Type	Width [min]	Area	Height	Area%	Name
18.540	MM m	0.3	82.4	18.7	0.13	D Leu
20.253	MM m	0.9	63765.5	14655.5	99.87	L Leu

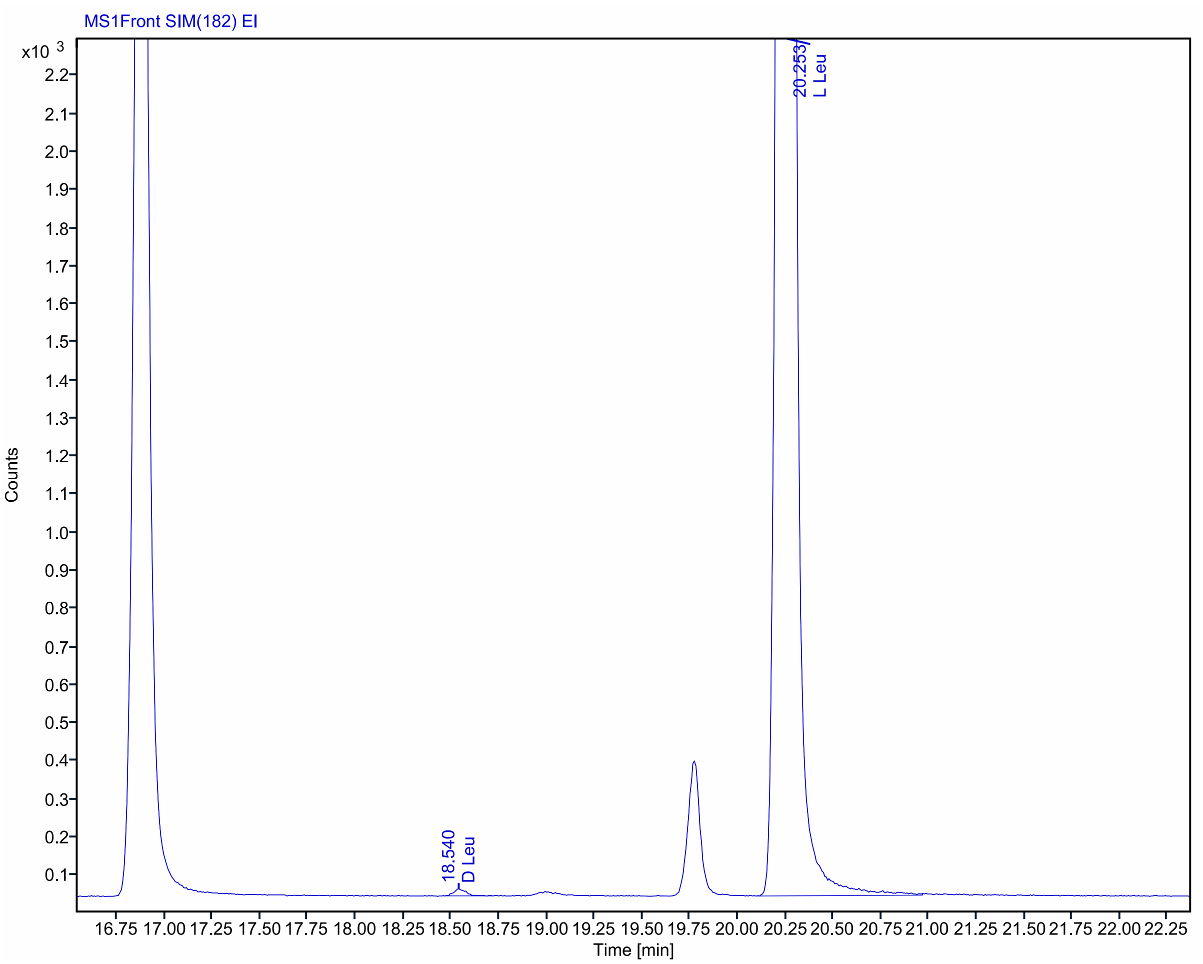

E-Signature

Level	Level Name	Signed By	Date	Comment	Meaning
1	Approver	Verena Pohl (verena)	2023-04-20 11:02:57+02:00		Approved

Chromatogram Report

**Data file**	/HP7/Results/2023_04/XY020_4.rslt\XY020-02-01_1A2.dx		
**Sample name**	Lira-25 mmol-no wash-purified		
**Instrument**	HP#7	**Location**	92
**Injection date**	04/19/2023 17:55:12	**Injection volume [µl]**	0.100
**Acq. method**	S1382_ET_AAA_SIM_A.amx	**Acq. operator**	Verena Pohl (verena)
**Analysis method**	ET_AAA.pmx		

Signal: MS1Front SIM(138) EI

RT [min]	Type	Width [min]	Area	Height	Area%	Name
11.468	MM m	0.07	1106.6	639.8	0.05	D Ser
11.569	MM m	0.37	2014579.7	1131560.8	99.95	L Ser

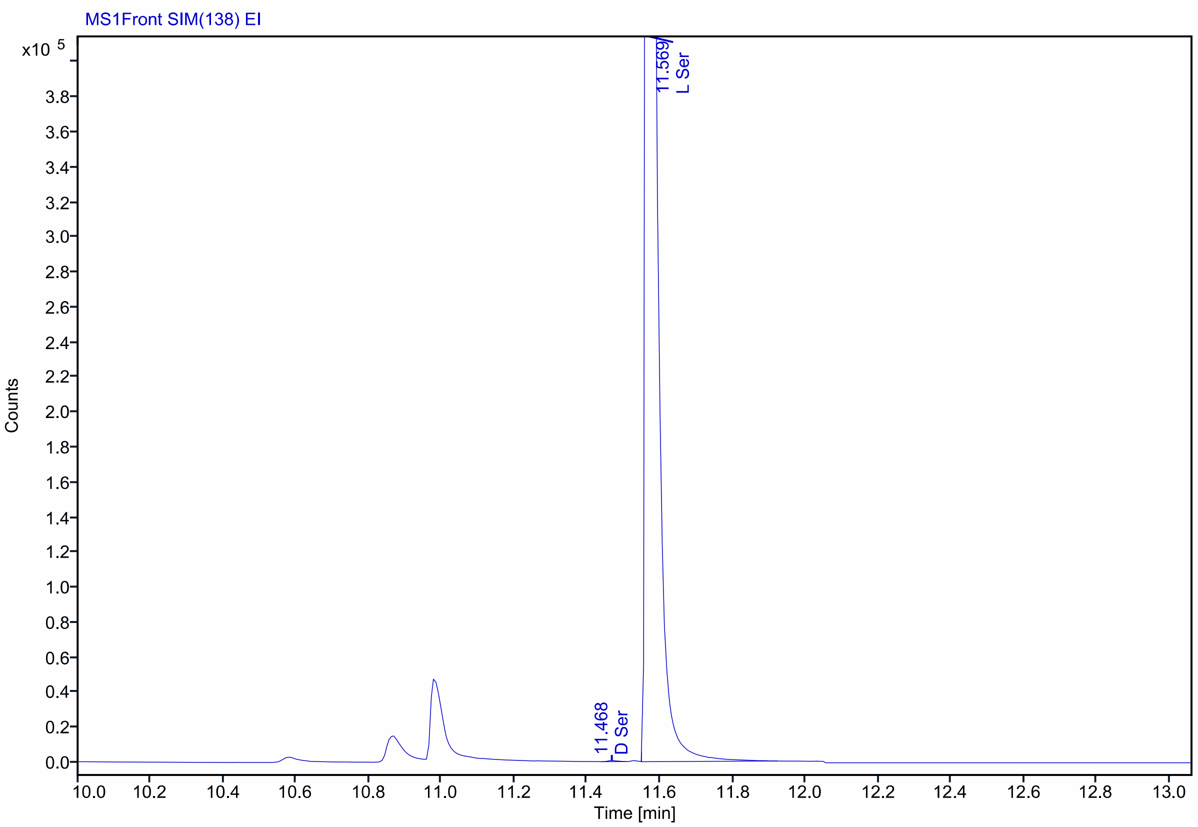

E-Signature

Level	Level Name	Signed By	Date	Comment	Meaning
1	Approver	Verena Pohl (verena)	2023-04-20 09:16:16+02:00		Approved

Chromatogram Report

**Data file**	/HP9/Results/2023_04/XY020_2.rslt\XY020-02-01_1A1.dx
**Sample name**	Lira-25 mmol-no wash-purified

**Instrument**	HP#9	**Location**	94
**Injection date**	04/19/2023 13:57:49	**Injection volume [µl]**	0.200
**Acq. method**	S1419_ET_AAA_SIM_B.amx	**Acq. operator**	Verena Pohl (verena)
**Analysis method**	ET_AAA.pmx		

Signal: MS1Front SIM(214) EI

RT [min]	Type	Width [min]	Area	Height	Area%	Name
24.333	BB	0.14	126.3	39.8	0.41	D Asp
24.517	BM m	0.57	30997.5	10850.6	99.59	L Asp

M-1 contribution of 0.23% must be subtracted

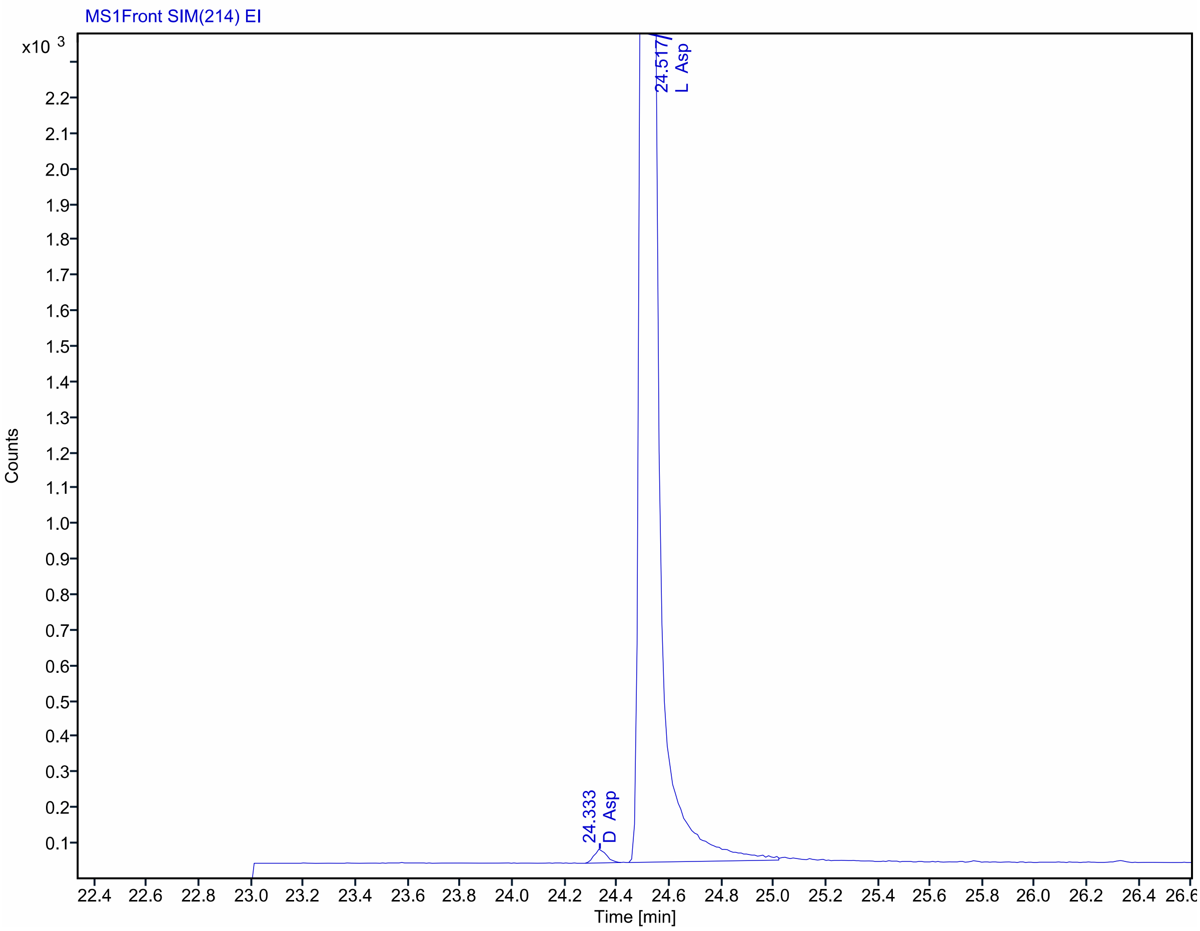

E-Signature

Level	Level Name	Signed By	Date	Comment	Meaning
1	Approver	Verena Pohl (verena)	2023-04-20 11:02:57+02:00		Approved

Chromatogram Report

**Data file**	/HP9/Results/2023_04/XY020_1.rslt\XY020-02-01_1EVY1.dx
**Sample name**	Lira-25 mmol-no wash-purified

**Instrument**	HP#9	**Location**	92
**Injection date**	04/19/2023 12:22:24	**Injection volume [µl]**	0.200
**Acq. method**	S1419_ET_AAA_SIM_B.amx	**Acq. operator**	Verena Pohl (verena)
**Analysis method**	ET_AAA.pmx		

Signal: MS1Front SIM(176) EI

RT [min]	Type	Width [min]	Area	Height	Area%	Name
27.148	MM m	0.11	127.9	58.4	0.14	D Phe
27.380	MM m	0.34	91904.9	50673.0	99.86	L Phe

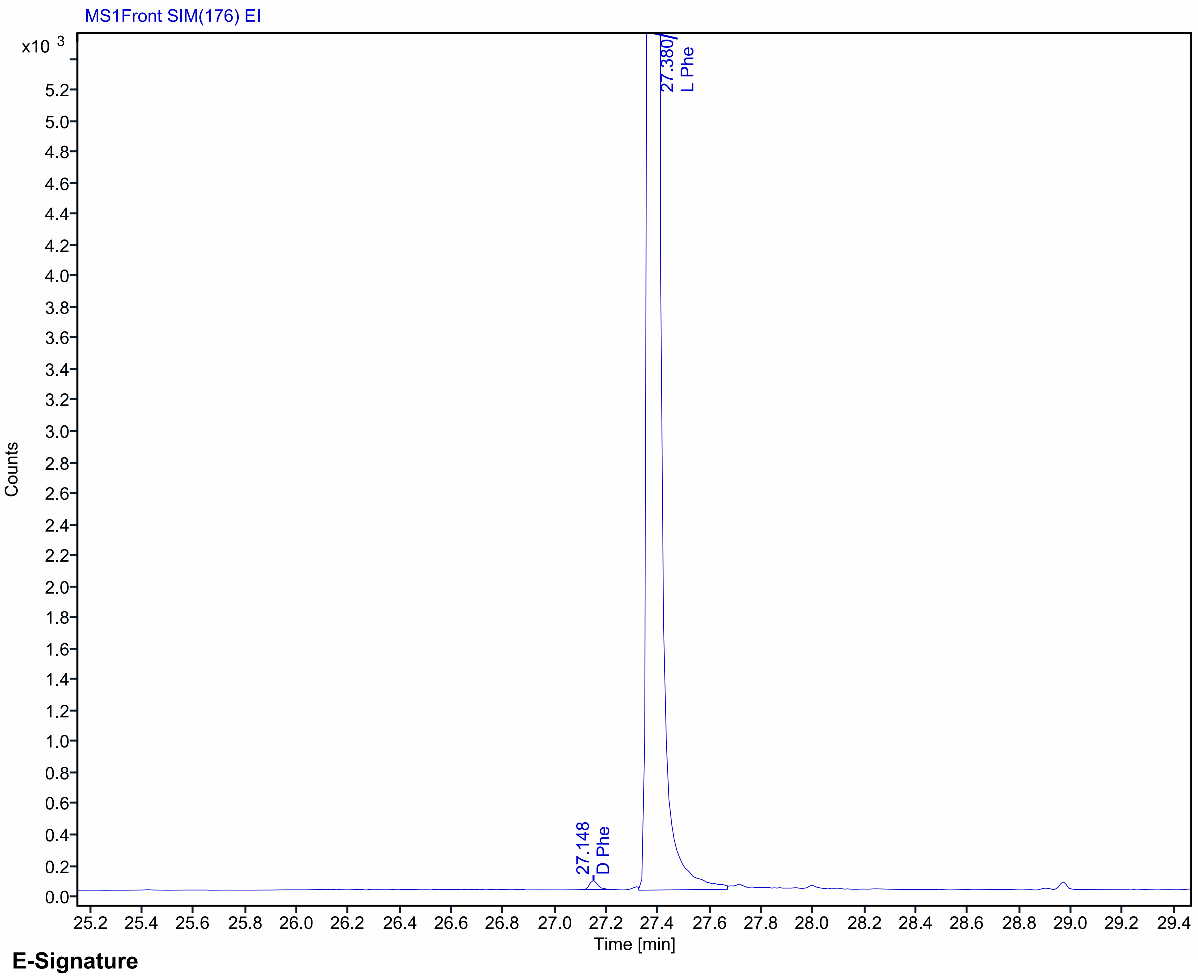

E-Signature

Level	Level Name	Signed By	Date	Comment	Meaning
1	Approver	Verena Pohl (verena)	2023-04-20 11:12:24+02:00		Approved

Chromatogram Report

**Data file**	/HP9/Results/2023_04/XY020_1.rslt\XY020-02-01_1EVY1.dx
**Sample name**	Lira-25 mmol-no wash-purified

**Instrument**	HP#9	**Location**	92
**Injection date**	04/19/2023 12:22:24	**Injection volume [µl]**	0.200
**Acq. method**	S1419_ET_AAA_SIM_B.amx	**Acq. operator**	Verena Pohl (verena)
**Analysis method**	ET_AAA.pmx		

Signal: MS1Front SIM(226) EI

RT [min]	Type	Width [min]	Area	Height	Area%	Name
27.072	MM m	0.11	63.9	31.1	0.25	D Glu
27.318	MM m	0.13	25337.8	15858.9	99.75	L Glu

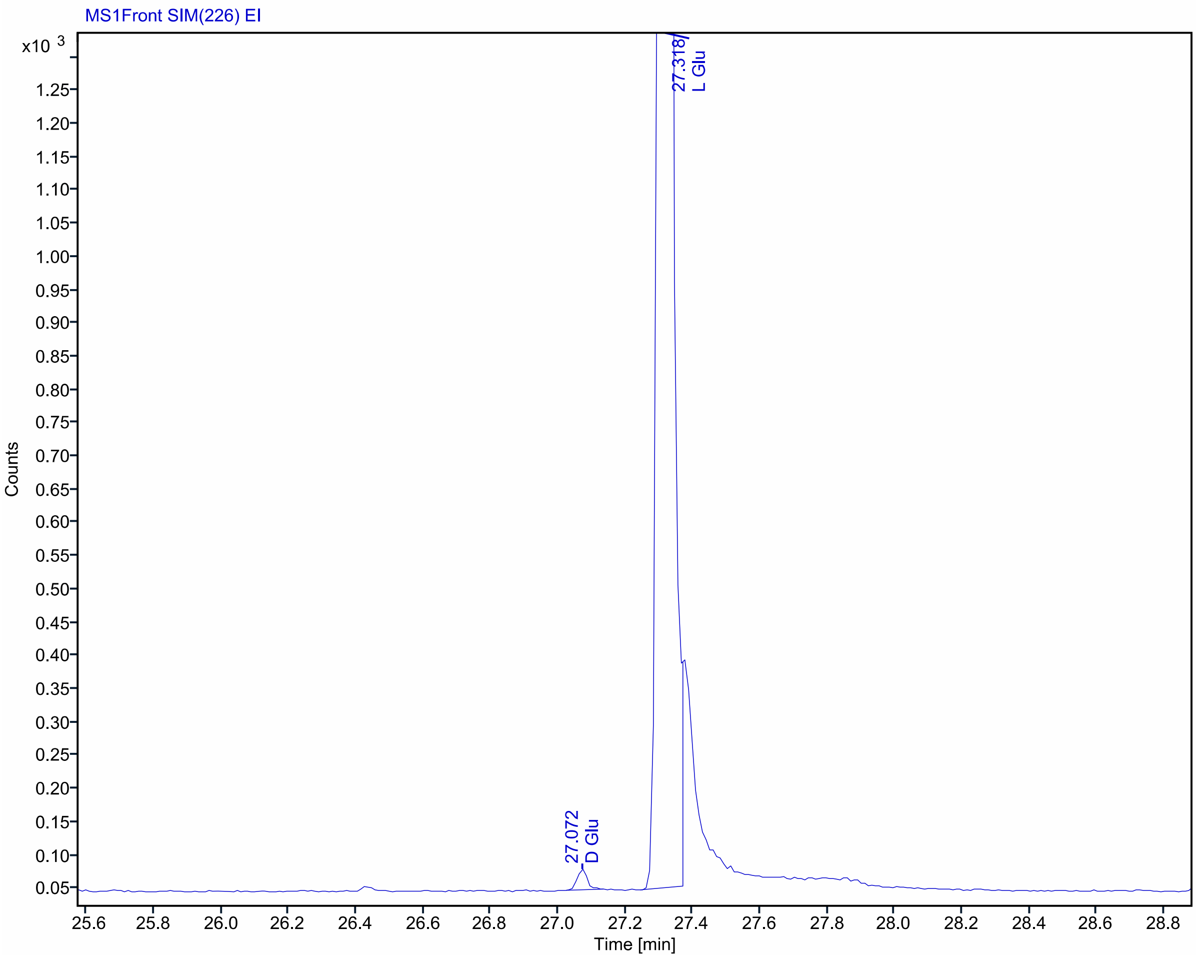

E-Signature

Level	Level Name	Signed By	Date	Comment	Meaning
1	Approver	Verena Pohl (verena)	2023-04-20 11:12:24+02:00		Approved

Chromatogram Report

**Data file**	/HP9/Results/2023_04/XY020_1.rslt\XY020-02-01_1EVY1.dx
**Sample name**	Lira-25 mmol-no wash-purified

**Instrument**	HP#9	**Location**	92
**Injection date**	04/19/2023 12:22:24	**Injection volume [µl]**	0.200
**Acq. method**	S1419_ET_AAA_SIM_B.amx	**Acq. operator**	Verena Pohl (verena)
**Analysis method**	ET_AAA.pmx		

Signal: MS1Front SIM(288) EI

RT [min]	Type	Width [min]	Area	Height	Area%	Name
29.828	MM m	0.08	14.9	8.5	0.07	D Tyr
30.011	MM m	0.39	21385.3	9850.0	99.93	L Tyr

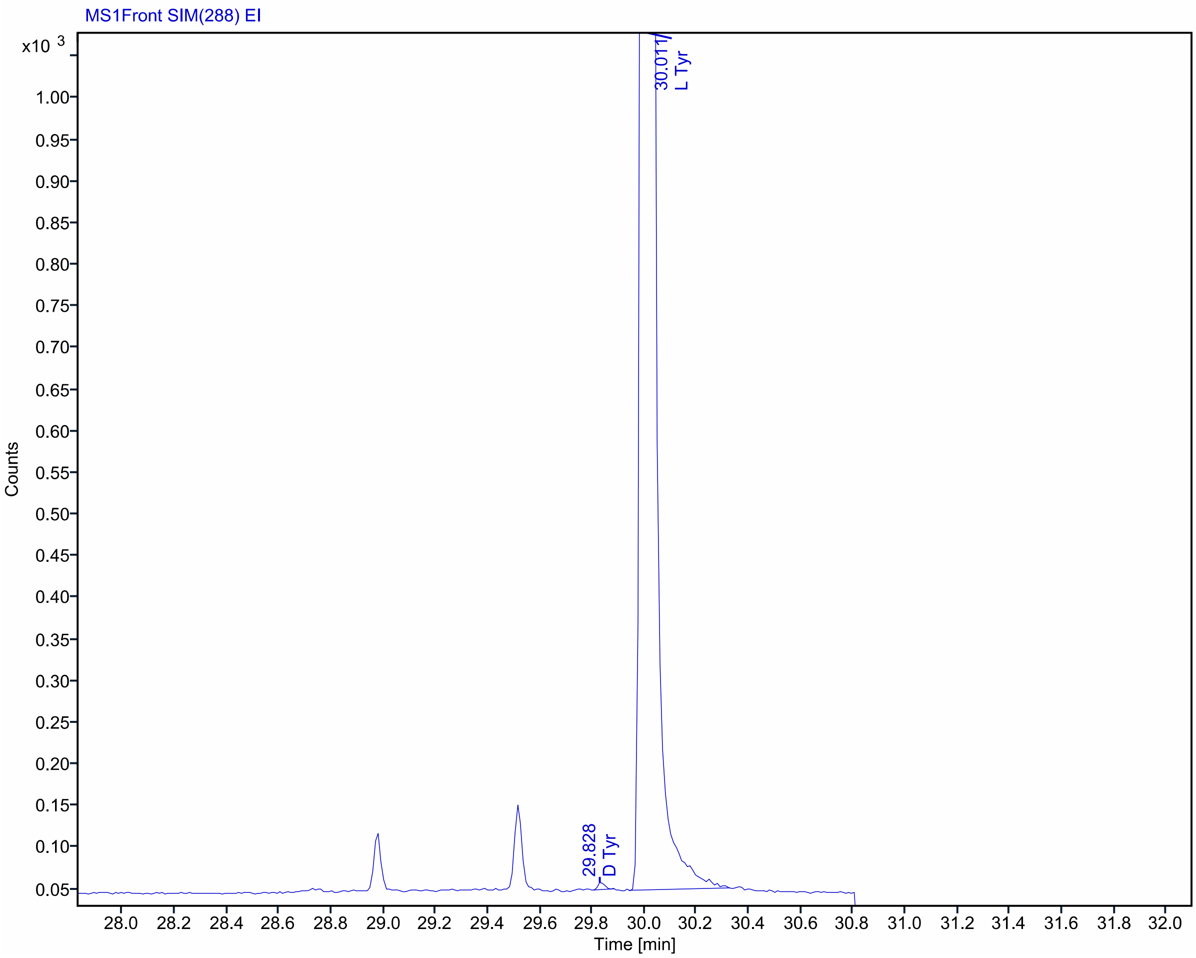

E-Signature

Level	Level Name	Signed By	Date	Comment	Meaning
1	Approver	Verena Pohl (verena)	2023-04-20 11:12:24+02:00		Approved

Chromatogram Report

**Data file**	/HP9/Results/2023_04/XY020_1.rslt\XY020-02-01_1EVY1.dx
**Sample name**	Lira-25 mmol-no wash-purified

**Instrument**	HP#9	**Location**	92
**Injection date**	04/19/2023 12:22:24	**Injection volume [µl]**	0.200
**Acq. method**	S1419_ET_AAA_SIM_B.amx	**Acq. operator**	Verena Pohl (verena)
**Analysis method**	ET_AAA.pmx		

Signal: MS1Front SIM(180) EI

RT [min]	Type	Width [min]	Area	Height	Area%	Name
32.297	MM m	0.10	77.4	22.9	0.13	D Lys
32.476	MM m	0.50	60475.5	22431.5	99.87	L Lys

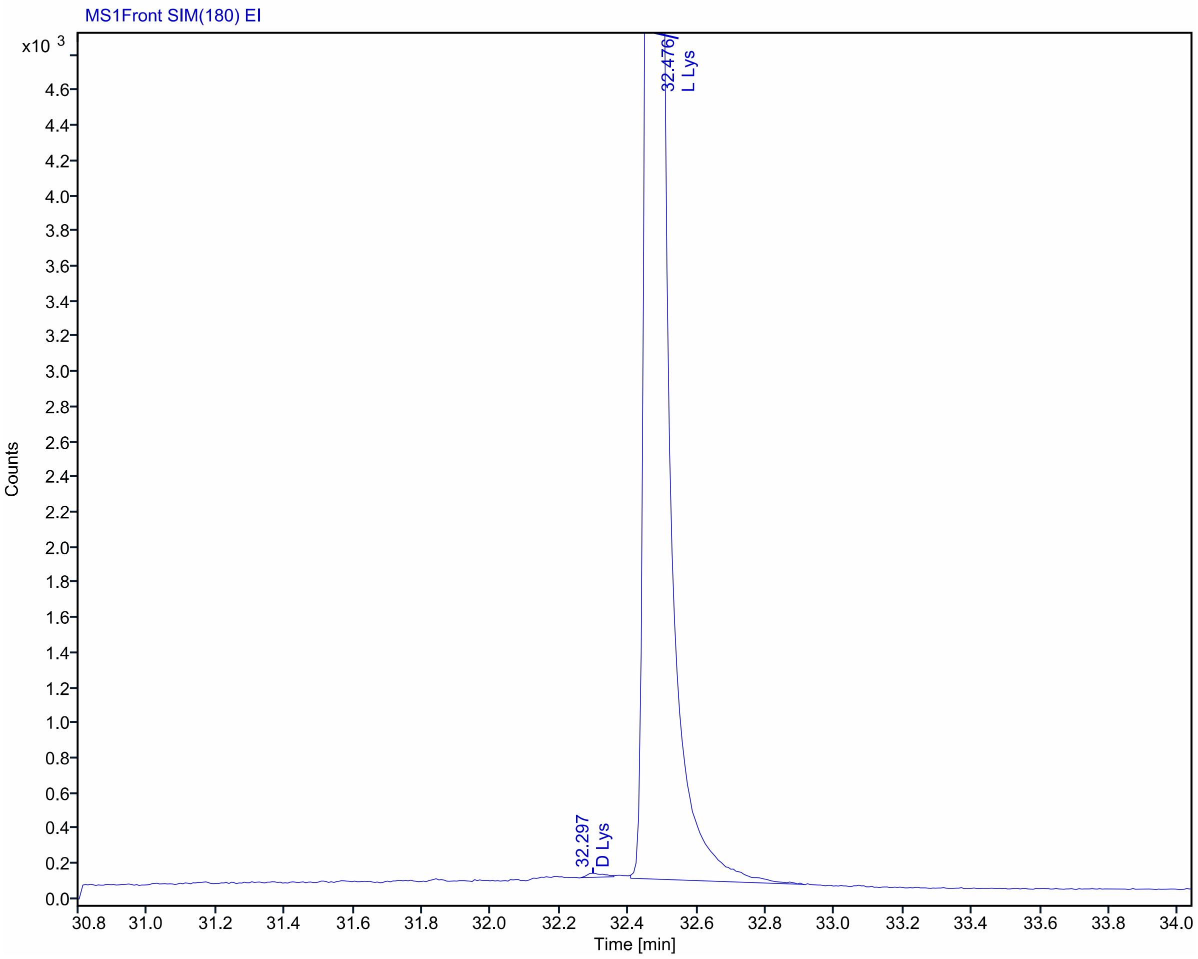

E-Signature

Level	Level Name	Signed By	Date	Comment	Meaning
1	Approver	Verena Pohl (verena)	2023-04-20 11:12:24+02:00		Approved

Chromatogram Report

**Data file**	/HP9/Results/2023_04/XY020_2.rslt\XY020-02-01_1A1.dx		
**Sample name**	Lira-25 mmol-no wash-purified		
**Instrument**	HP#9	**Location**	94
**Injection date**	04/19/2023 13:57:49	**Injection volume [µl]**	0.200
**Acq. method**	S1419_ET_AAA_SIM_B.amx	**Acq. operator**	Verena Pohl (verena)
**Analysis method**	ET_AAA.pmx		

Signal: MS1Front SIM(421) EI

RT [min]	Type	Width [min]	Area	Height	Area%	Name
34.206	MM m	0.12	42.5	11.4	0.10	D Arg
34.457	BBA	0.75	40578.5	10879.9	99.90	L Arg

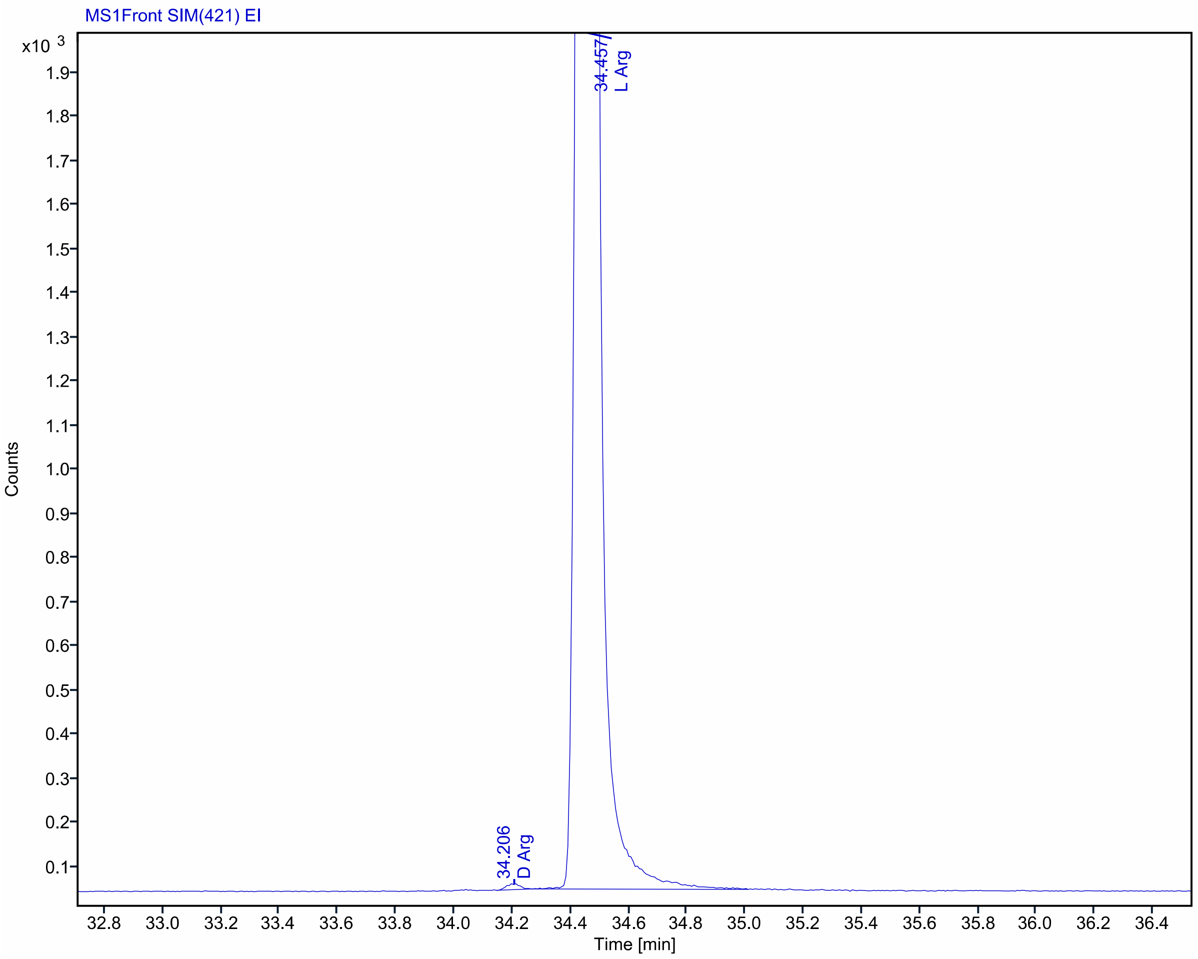

E-Signature

Level	Level Name	Signed By	Date	Comment	Meaning
1	Approver	Verena Pohl (verena)	2023-04-20 11:02:57+02:00		Approved

Chromatogram Report

**Data file**	/HP9/Results/2023_04/XY020_3.rslt\XY020-02-01_1EVY2.dx		
**Sample name**	Lira-25 mmol-no wash-purified		
**Instrument**	HP#9	**Location**	92
**Injection date**	04/19/2023 23:29:10	**Injection volume [µl]**	1.000
**Acq. method**	S1419_ET_AAA_WRH_A.amx	**Acq. operator**	Verena Pohl (verena)
**Analysis method**	ET_WRH.pmx		

Signal: MS1Front SIM(311) EI

RT [min]	Type	Width [min]	Area	Height	Area%	Name
16.816	MM m	0.06	3.9	2.0	100.00	L Trp

M-1 contribution of 0.17% must be subtracted

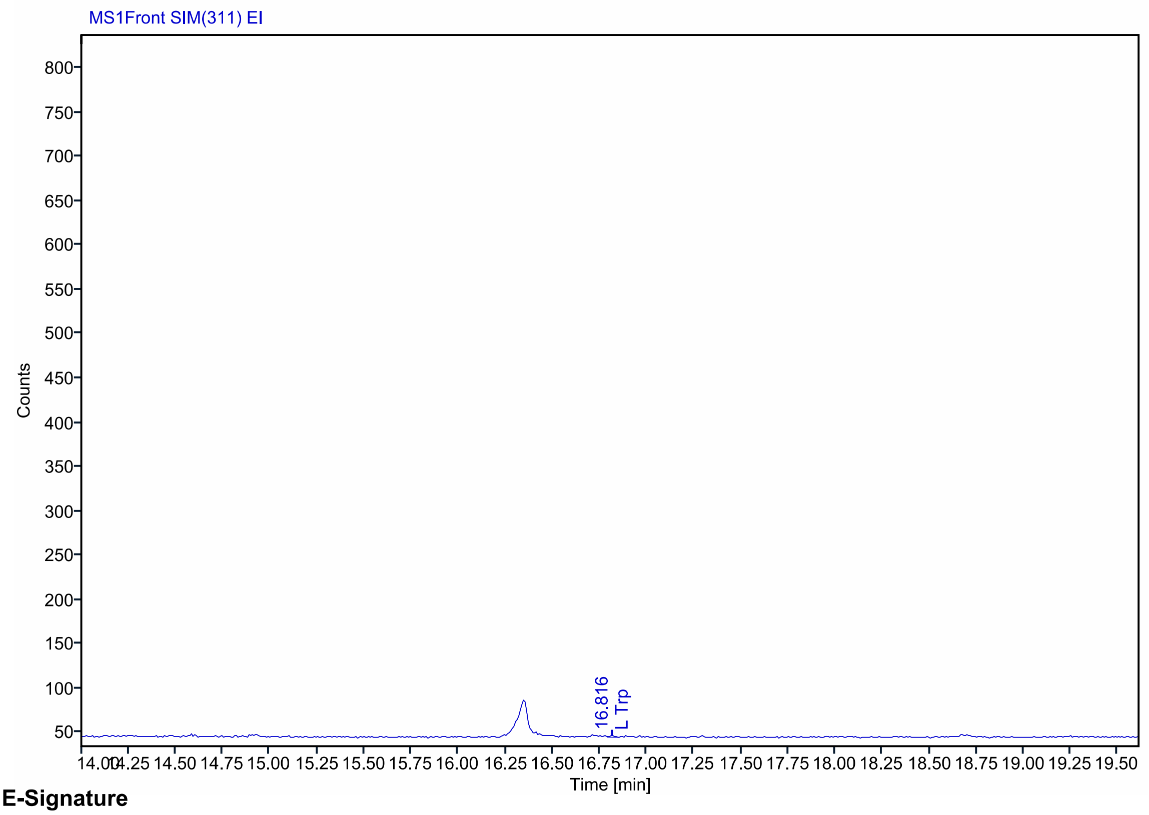

E-Signature

Level	Level Name	Signed By	Date	Comment	Meaning
1	Approver	Verena Pohl (verena)	2023-04-20 09:04:51+02:00		Approved

Chromatogram Report

**Data file**	/HP9/Results/2023_04/XY020_5.rslt\XY020-02-01_1A3.dx		
**Sample name**	Lira-25 mmol-no wash-purified		
**Instrument**	HP#9	**Location**	94
**Injection date**	04/20/2023 11:32:42	**Injection volume [µl]**	0.200
**Acq. method**	S1419_ET_AAA_WRH_A.amx	**Acq. operator**	Verena Pohl (verena)
**Analysis method**	*ET_WRH.pmx		

Signal: MS1Front SIM(379) EI

RT [min]	Type	Width [min]	Area	Height	Area%	Name
17.722	MM m	0.17	15.2	3.1	0.47	D His
17.971	MM m	0.69	3207.2	615.0	99.53	L His

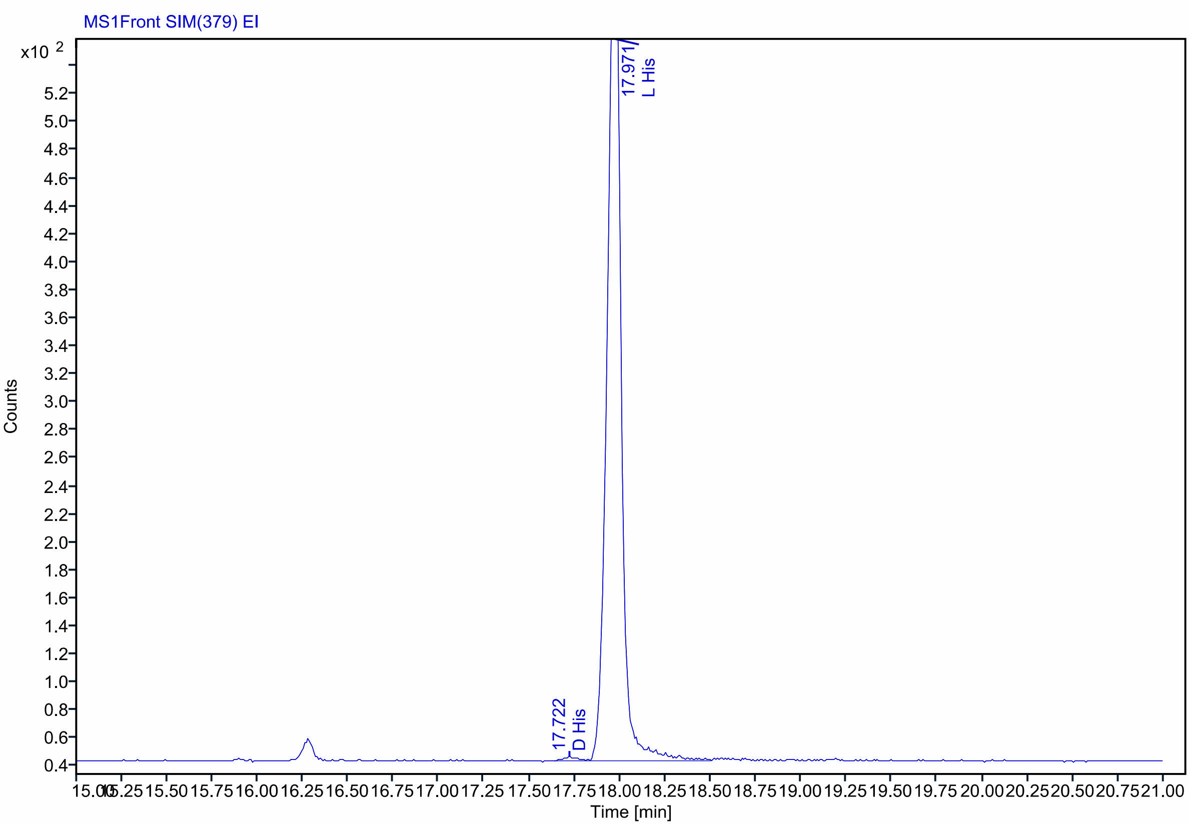

E-Signature

Level	Level Name	Signed By	Date	Comment	Meaning
1	Approver	Verena Pohl (verena)	2023-04-20 13:48:53+02:00		Approved

Supplementary Table 3 Stepwise method details for wash-free 0.1 mmol research scale synthesis.

Wash-free method for 0.1 mmol synthesis (using one-pot methodology)	
**Cycle steps**	**Operations**
**1. Resin swelling**	**1.1.** Load 0.1 mmol resin in 30 mL reaction vessel<br>**1.2.** Add DMF for resin swelling: 4 mL<br>**1.3.** Start bubbling (from bottom of reaction vessel) with N_2<br>**1.4.** Start resin swelling method: 5 min/RT followed by drain to remove DMF
**2. First Deprotection**	**2.1.** Add DMF: 3.5 mL<br>**2.2.** Add deprotection reagent: 0.75 mL (17% pyrrolidine in DMF)<br>Total deprotection volume in reaction vessel = 4.25 mL<br>**2.3.** Start deprotection microwave method: 80 sec/110 ^oC<br>**2.4.** Start bubbling (from bottom of reaction vessel) with N_2<br>**2.5.** Start headspace flushing (from top of reaction vessel) with N_2 flow rate at 3.5 L/min
**3. Drain**	**3.1.** Drain all leftover reagents and byproducts.<br>*Deprotected peptide resin is left behind in the reaction vessel.*
**4. Coupling**	**4.1.** Add reagents for coupling in the following order:<br>Amino acid: 1 mL (0.5 M in DMF)<br>DIC: 1 mL (0.75 M in DMF)<br>Oxyma: 1.5 mL (0.26 M in DMF)<br>Total coupling volume in reaction vessel = 3.5 mL<br>**4.2.** Start coupling microwave method: 30 sec/RT + 60 sec/105 ^oC<br>**4.3.** Start bubbling (from bottom of reaction vessel) with N_2
**5. Deprotection**	**5.1.** Add deprotection reagent: 0.75 mL (17% pyrrolidine in DMF) to the undrained post-coupling mixture in step 4<br>Total deprotection volume in reaction vessel = 4.25 mL<br>**5.2.** Start deprotection microwave method: 80 sec/110 ^oC<br>**5.3.** Start bubbling (from bottom of reaction vessel) with N_2<br>**5.4.** Start headspace flushing (from top of reaction vessel) with N_2 flow rate at 3.5 L/min
**6. Drain**	**6.1.** Drain all leftover reagents and byproducts.<br>*Deprotected peptide resin is left behind in the reaction vessel.*
Repeat steps 4-6 for all remaining Fmoc-amino acid residues in the sequence	
**7. Final deprotection**	**7.1.** Repeat step 5 To remove Fmoc protection from the final amino acid residue in the sequence

At the end of final Fmoc deprotection, the peptide resin is collected from the reaction vessel for cleavage and analysis.

Supplementary Table 4 Stepwise method details for wash-free 25 mmol production scale synthesis.

Wash-free method for 25 mmol synthesis	
**Cycle steps**	**Operations**
**1. Resin swelling**	**1.1.** Load 25 mmol resin in 3 L reaction vessel<br>**1.2.** Add DMF for resin swelling: 500 mL<br>**1.3.** Start stirring and bubbling (from bottom of reaction vessel) with N_2 flow rate at 10 L/min<br>**1.4.** Start resin swelling method: 10 min/RT followed by drain to remove DMF
**2. Deprotection**	**2.1.** Add deprotection reagent: 50 mL (15% pyrrolidine in DMF)<br>**2.2.** Add DMF to clean reagent line with purge: 250 mL<br>Total deprotection volume in reaction vessel = 475 mL<br>**2.3.** Start deprotection microwave method: 10 min/90 ^{o}C<br>**2.4.** Start stirring and bubbling (from bottom of reaction vessel) with N_2 flow rate at 10 L/min<br>**2.5.** Start headspace flushing (from top of reaction vessel) with N_2 flow rate at 85 L/min
**3. Drain**	**3.1.** Drain all leftover reagents and byproducts.<br>*Deprotected peptide resin is left behind in the reaction vessel.*
**4. Coupling**	**4.1.** Add reagents for coupling in the following order:<br>Amino acid: 200 mL (0.5 M in DMF)<br>DIC: 50 mL (4 M in DMF)<br>Oxyma: 225 mL (0.33 M in DMF)<br>Total coupling volume in reaction vessel = 475 mL<br>**4.2.** Start coupling microwave method: 5 min/80 ^{o}C<br>**4.3.** Start stirring and bubbling (from bottom of reaction vessel) with N_2 flow rate at 10 L/min
**5. Drain**	**5.1.** Drain all leftover reagents and byproducts.<br>*Fmoc-protected peptide resin is left behind in the reaction vessel.*
Repeat steps 2-5 for all remaining Fmoc-amino acid residues in the sequence	
**6. Final deprotection**	**6.1.** Repeat step 2 To remove Fmoc protection from the final amino acid residue in the sequence

At the end of final Fmoc deprotection, the peptide resin is collected from the reaction vessel for cleavage and analysis.

Supplementary Table 5 Stepwise method details for residual pyrrolidine quantification by GC-FID.

Residual pyrrolidine experiment	
Cycle Steps	Operation
1. Resin Swelling and loading	1.1 Resin (0.1 mmol) was preswelled in DMF (8 mL) for 10 min and then added to a clean and dry reaction vessel.<br>1.2 The reaction vessel was drained, and washed with 10 mL DMF
2. First Deprotection	2.1 Add DMF: 3.5 mL<br>2.2 Add deprotection reagent: 0.75 mL (17% pyrrolidine in DMF). Total deprotection volume in the reaction vessel = 4.25 mL<br>2.3 Start deprotection microwave method: 80 sec/110 °C 2.4<br>2.4 Start bubbling (from bottom of reaction vessel) with N_2<br>2.5 Start headspace flushing (from top of reaction vessel) with N_2 flow rate at 3.5 L/min
3. Drain	Drain all reagents through frit using N_2 pressure from top of vessel. *Deprotected peptide resin is left behind in the reaction vessel.*
4. Coupling	4.1 Add reagents for coupling in the following order: Amino acid: 1 mL (0.5 M in DMF), DIC: 1 mL (0.75 M in DMF), and Oxyma: 1.5 mL (0.26 M in DMF). Total coupling volume = 3.5 mL<br>4.2 Start coupling microwave method: 30 sec/RT + 60 sec/105 °C<br>4.3 Start bubbling (from bottom of reaction vessel) with N2
5. Deprotection	5.1 Add deprotection reagent: 0.75 mL (17% pyrrolidine in DMF) to the undrained post-coupling mixture in step 4. Total deprotection volume in the reaction vessel = 4.25 mL<br>5.2 Start deprotection microwave method: 80 sec/110 °C<br>5.3 Start bubbling (from bottom of reaction vessel) with N_2<br>5.4 Start headspace flushing (from top of reaction vessel) with N_2 flow rate at 3.5 L/min
6. Drain	6.1 Drain all reagents through frit using N_2 pressure from top of vessel. *Deprotected peptide resin is left behind in the reaction vessel.*
7. Repeat steps 4–6 twice more	7.1 By the end of step 7, the method will have completed 1 first deprotection, 3 regular deprotections, and 3 couplings; leaving the reaction vessel in a drained post-deprotection state.
8. Add DMF and collect sample for GC- analysis	8.1 After drain is complete, add 3.5 mL of DMF (equivalent volume to coupling mixture) to the resin in the reaction vessel

	8.2 Remove reaction vessel from microwave cavity and allow temperature to cool from 56°C to 25°C<br>8.3 Open reaction vessel and pipette 100 µL of the solution to a vial while avoiding the uptake of resin beads.<br>8.4 The aliquot was diluted 100-fold by serial dilution with HPLC-grade IPA (1:10, repeated twice) and then inject into the GC-FID for analysis

==== Shimadzu LabSolutions Calibration Curve ====

ID# : 1
Name : Pyrrolidine
Quantitative Method : External Standard
Function : f(x)=83769.2*x-2634.55
Rr1=0.9997946 Rr2=0.9995893 RSS=4.476877e+007
MeanRF: 9.129169e+004 RFSD: 2.597406e+004 RFRSD: 28.451719
FitType : Linear
ZeroThrough : Not Through
Weighted Regression : None
Detector Name : FID1

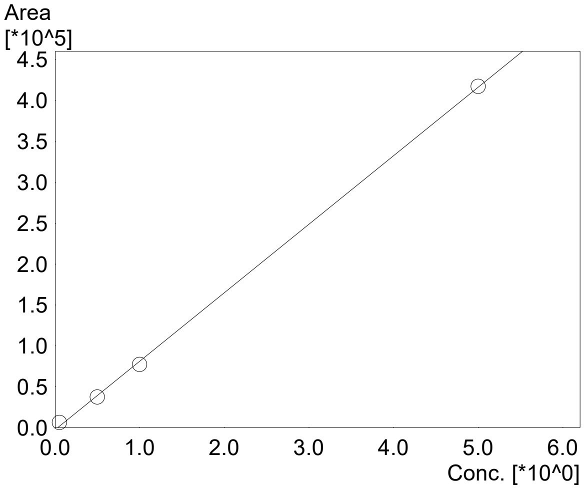

#	Conc.(Ratio)	MeanArea	Area
1	0.05	6493	6493
3	0.5	37366	37366
4	1	77141	77141
6	5	417149	417149

LabSolutions – Representative pyrrolidine standard, 0.5% pyrrolidine in DMF

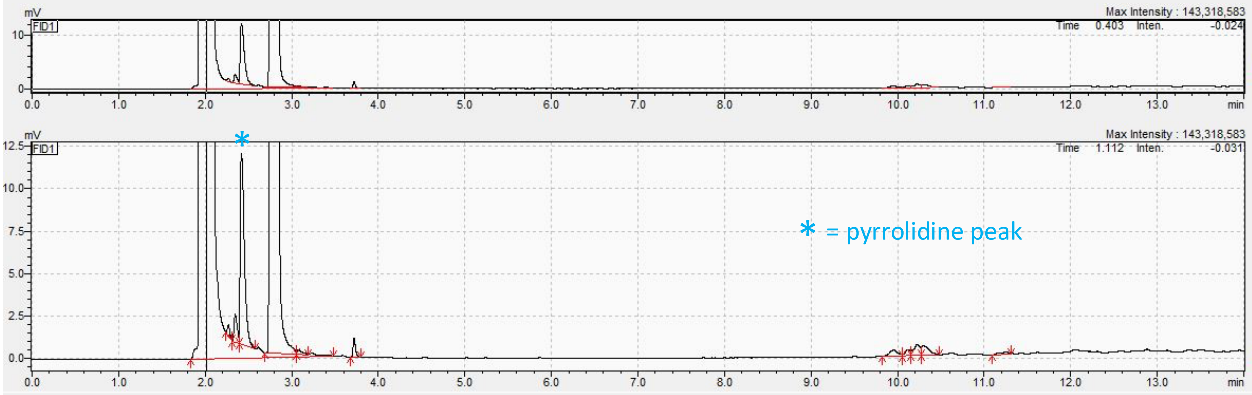

Peak#	Ret. Time	Area	Height	Conc. (ppm)	Name
1	2.018	647922548	143053652		IPA
2	2.268	1147	608		
3	2.347	4203	1625		
4	2.42	37366	11221	4775	pyrrolidine
5	2.811	4561418	1476155		DMF
6	3.079	1114	280		
7	3.72	2053	1089		
8	9.954	2227	351		
9	10.116	1392	360		
10	10.228	3326	645		
11	10.301	3397	559		
12	11.245	1019	126		

LabSolutions – Real Sample #1, optimized conditions, 80 s deprotection, 10 s drain, headspace flushing ON

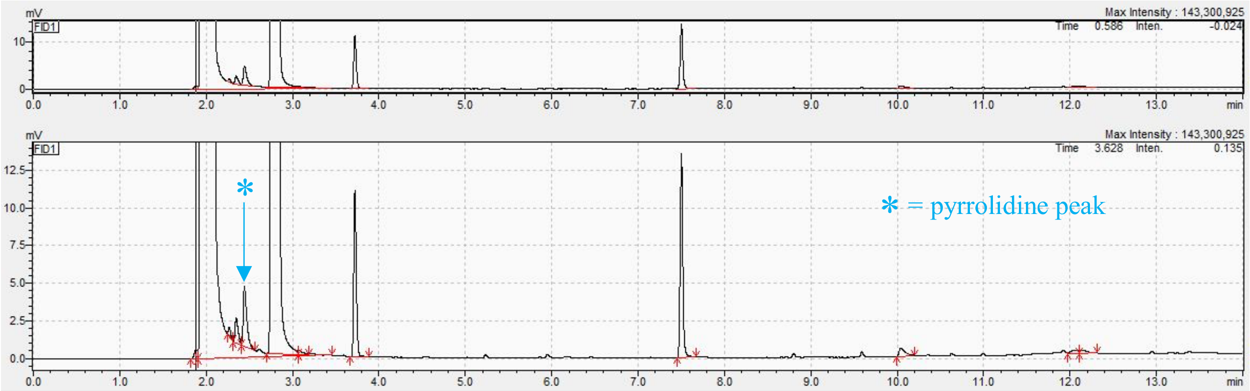

Peak#	Ret. Time	Area	Height	Conc. (ppm)	Name
1	1.885	1530	569		
2	2.019	651578198	142819863		IPA
3	2.269	1039	578		
4	2.348	4211	1647		
5	2.443	11436	3957	1680	pyrrolidine
6	2.812	4523719	1459906		DMF
7	3.08	1098	276		
8	3.722	22481	10964		
9	7.503	28507	13477		
10	10.046	2642	545		
11	12.046	1137	186		
12	12.153	1286	212		
